# A national consensus-based framework on preferred assessments and interventions in current treatment for young people with acquired brain injury in Dutch rehabilitation centers

**DOI:** 10.1177/18758894251337581

**Published:** 2025-04-23

**Authors:** Florian Allonsius, Arend de Kloet, Frederike van Markus-Doornbosch, Ingrid Rentinck, Suzanne Lambregts, Karin Huizing, Peter de Koning, Sandra te Winkel, Christine Resch, Thea Vliet Vlieland, Menno van der Holst

**Affiliations:** 1 Department of Innovation, Quality and Research, Basalt Rehabilitation Center, The Hague, The Netherlands; 2 Department of Orthopedics, Rehabilitation and Physical Therapy, Leiden University Medical Center, Leiden, The Netherlands; 3 Department of Pediatric Psychology, Sector of Neuropsychology, University Medical Center Utrecht, Utrecht, The Netherlands; 4 Department of Pediatric Rehabilitation, De Hoogstraat Rehabilitation, Utrecht, The Netherlands; 5 Department of Pediatric Rehabilitation, Revant Rehabilitation Center, Breda, The Netherlands; 6 Department of Pediatric Rehabilitation, Rehabilitation Center ‘Revalidatie Friesland’, Beetsterzwaag, The Netherlands; 7 Department of Pediatric Rehabilitation, Heliomare Rehabilitation Center, Wijk aan Zee, The Netherlands; 8 Department of Pediatric Rehabilitation, Merem Medical Rehabilitation, Hilversum, The Netherlands; 9 Department of Neuropsychology & Psychopharmacology, Maastricht University, Maastricht, The Netherlands; 10 Department of Pediatric Rehabilitation, Limburg Brain Injury Center, Maastricht, The Netherlands

**Keywords:** health care organizations and systems, pediatrics, clinical practice patterns, guidelines, resource use, evidence based practice

## Abstract

**Purpose:**

Acquired brain injury (ABI) is prevalent among young people (4–25 years). When ABI-related problems persist, treatment in a rehabilitation center (RC) may be indicated. However, there is wide variability regarding the delivery of care across Dutch RCs, including assessments, interventions, and psychoeducational (PE) materials. The aim was to create a consensus-based framework with preferred assessments, interventions, and PE-materials to be used in pediatric ABI rehabilitation. A national framework could optimize the delivery of comparable care for this population.

**Methods:**

For this three-round Delphi study, healthcare professionals (physiatrists, psychologists, social workers, physical/occupational/speech/language therapists) from RCs providing care for young people with ABI were invited to participate. In the first two (online) rounds, currently used assessments/interventions/PE-materials were collected, stepwise-prioritized, subsequently listed per discipline, and classified per International Classification of Functioning (ICF) domain. Results from rounds one/two were discussed in a consensus meeting (in person), aiming to reach agreement on assessments/interventions/PE-materials in the national framework and how to use them in current practice.

**Results:**

Seventy-four healthcare professionals from 12 RCs participated. After Delphi round one, 163 assessments, 39 interventions, and 64 PE-materials were collected. After round two, the selection was narrowed down to n = 51/n = 34/n = 28, respectively. After round three, consensus was reached on 37 assessments, 25 interventions (divided over all disciplines/classified per ICF domain), 27 PE-materials, as well as on the use of the framework by all participating RC to enhance clinical reasoning in current practice.

**Conclusion:**

A consensus-based national framework in ABI rehabilitation has been developed and is now available to optimize the delivery of care for young people with ABI across Dutch RCs.

## Introduction

Acquired brain injury (ABI) is a comprehensive term for brain damage that occurs after birth including traumatic brain injury (TBI) and non-traumatic brain injury (nTBI).^1^ ABI is prevalent in young people under the age of 25^2,3^ and can lead to significant disruptions in the development of a young person. It is known to be a leading cause of disability in this age group worldwide,^2,3^ as well as in the Netherlands.^4^ Young people with ABI constitute a heterogeneous population in terms of age, type of injury, injury severity, socioeconomic status, and impairment level, as well as in perceived limitations in activities and restrictions to participation.^5–7^ For persisting problems in daily life, young patients may require rehabilitation treatment in specialized, multidisciplinary teams.^8–11^

Several studies on the effectiveness of rehabilitation treatment for individuals with disabilities, including young patients with ABI, have reported that the ultimate goal of rehabilitation treatment is optimal participation in society. The actual focus and content of rehabilitation treatment appeared to vary across these studies despite similarities in populations.^8,9,12–14^ Variability in the provision of rehabilitation treatment for young patients with ABI is not only observed in the literature,^8,9,12–14^ but also in daily practice. Despite the existence of a Dutch standard for quality of care for children (0–18 years) with TBI in the Netherlands,^15^ exact structures or rehabilitation content is lacking. Therefore, substantial room for variation in rehabilitation treatment across rehabilitation centers (RCs) is possible.^15^ Assessments (e.g., physical and cognitive) are considered particularly important in rehabilitation treatment and are widely used to determine a patient's current functioning, in goal-setting,^16–18^ as well as to evaluate interventions.^16,17^ It is likely that the variation in assessments and interventions may, in part, be related to the scarcity of practice guidelines or recommendations on the rehabilitation treatment of young patients with ABI. Practice variations described in the literature and observed in daily practice may be signalizing suboptimal care, as was described in previous studies on rehabilitation treatment in adult stroke and arthritis populations.^19,20^

The literature regarding the content of rehabilitation treatment for children and adolescents with ABI is scarce. Several studies have given an overview of assessments and interventions for rehabilitation populations.^13,21–25^ These studies focused on specific populations, i.e., adults with stroke and ABI,^21^ children with stroke,^22^ and children with ABI in the acute phase.^23^ However, while pediatric rehabilitation care is often provided to children aged 4–18 years (and, in some cases in the Netherlands, ages 19–25), these studies did not focus on multidisciplinary rehabilitation treatment for the population of young patients (4–25 years) with ABI (both TBI and nTBI) as a whole.^13,23–25^ Furthermore, psychoeducation (PE) is considered an important element of treatment interventions in pediatric ABI rehabilitation and many materials are available.^10,26^ However, a list specific to the population of young patients with ABI in the rehabilitation setting is lacking to date.

Rehabilitation professionals (e.g., physiatrists, psychologists, physical therapists, occupational therapists, speech/language therapists, social workers) in the Netherlands show a growing interest in standardizing assessments and interventions used in pediatric rehabilitation treatment. Creating structured rehabilitation frameworks that describe assessments and interventions is also in line with the principles of value-based healthcare (VBHC) to provide the best possible care for each individual child and their family.^27^

A national framework containing assessments, interventions, and PE-materials could decrease undesired practice variation and enhance the offering of comparable care for young patients with ABI regardless of where they live in the Netherlands. Further, it could stimulate collaborations and joint research projects across RCs in terms of cost-effectiveness and efficacy, which is also in line with the principles of VBHC.^27^ Therefore, the goal of the current study was to create a national consensus-based framework on preferred assessments, interventions and PE in current outpatient rehabilitation treatment for young people aged 4–25 years with ABI in Dutch RCs.

## Methods

### Design

In the current study, a three-round Delphi method was used to collect assessments and interventions utilized in rehabilitation treatment for children with ABI and to reach consensus among physiatrists and healthcare professionals across RCs regarding these assessments and interventions. The guidelines for the Delphi survey technique by Hasson et al. were used.^28^ Two Delphi rounds addressed preferred assessments and interventions using online questionnaires (e-Delphi method^29^), followed by a consensus meeting using a nominal group technique (group brainstorming through writing down, sharing, and voting on topics).^30^ A list of PE-materials used in current practice was also collected during the Delphi rounds.

### Setting

The current study was part of the multicenter project “Participate?! Next Step” (2021–2023) in which 14 Dutch RCs providing rehabilitation treatment for young patients between 4–25 years old with ABI participated. The project was led by a group that consisted of a PhD candidate (FA) and four senior researchers (AdK, FvM, TVV, MvdH). The project also had an advisory board consisting of physiatrists, psychologists, and senior researchers (n = 8), which included six of the authors (IR, SL, KH, PdK, StW, and CR). Their task was to advise and assist in designing and conceptualizing the project as well as the outlines of this Delphi study. The project and study protocol were reviewed and approved by the medical ethical review board of the Leiden University Medical Center. The local research committees from all participating RCs approved the larger project, including the current study.

### Recruitment of participants

The physiatrists and healthcare professionals who were involved in the project “Participate?! Next Step” within the participating RCs were asked to propose up to 12 of their colleagues (physiatrists/healthcare professionals, up to two per discipline) to participate in the Delphi study. Potential participants were eligible if they were (1) a physiatrist or a healthcare professional from one of the following disciplines: psychology, physical therapy (PT), occupational therapy (OT), speech/language therapy (SLT), or social work (SW); (2) working with children and/or adolescents and/or young adults (4–25 years old) with ABI in daily practice; and (3) willing to participate in all three rounds of the Delphi study. Subsequently, the project group provided information regarding the procedure and planning of the Delphi study to potential participants by e-mail.

First Delphi round: In the first round of the Delphi study, participants received a unique link (by e-mail) to access an online questionnaire containing five questions. The first two questions were general, i.e., the RC where employed, discipline, and years of experience working with young patients with ABI (<5/≥5 years). The years of experience cutoff was chosen based on recommendations from experts in the field who held a position on the advisory board of the project. The other three questions were discipline-specific questions, concerning assessments, interventions, and PE-materials used within their discipline in current practice. Participants were asked to provide any information available on the description and/or validity of the assessments, interventions, and PE-materials.

The participating physiatrists reviewed and added the assessments, interventions, and PE-materials that were proposed by the healthcare professionals in their own RCs.

The project group combined data from all completed questionnaires. The assessments, interventions, and PE-materials in daily practice across RCs in the first round were filtered for repeated listings. The surveys were conducted using Castor EDC.

In line with the current Dutch standard of practice-based care,^15^ assessments and interventions used in two or more of the participating RCs were included in the list for the second round. Thereafter, they were categorized by discipline (where applicable) and classified by the International Classification of Functioning (ICF) domains (body functions, activities and participation, environmental factors, and body structures),^31^ through ICF linking rules.^32^

All PE-materials that were used in two or more of the participating RCs were included in the list and proposed for the second Delphi round.

Second Delphi round: The participants who filled out the questionnaire in the first round were asked to participate in the second round. For every assessment, intervention and PE-material that was selected from the first round, participants were asked whether they thought it should be included in the national framework on current practice (yes/no).

After collecting the results of the second round, the project group used a level of agreement to reach consensus.

- When ≥75% of the respondents answered ‘yes’ to a proposed assessment/intervention/PE-material, the item was included in the concept framework.
- If 75% or more (≥) of the answers were ‘no,’ the assessment/intervention/PE-material was rejected.
- If 25–75% of the answers per item were ‘yes,’ the assessment/intervention/PE-material was put on a list to be discussed in the third Delphi round.

Third Delphi round: The third Delphi round consisted of an in-person meeting of approximately four hours to discuss and reach consensus on the results of the first two rounds. Approximately two weeks prior to the meeting, all participants from the RCs received the concept framework in preparation for the meeting. One rehabilitation physiatrist and one other healthcare professional (either a psychologist, PT, OT, SLT, or SW) from each RC were allowed to be present due to national restrictions during the COVID-19 pandemic at the time. They were asked to represent their RC as a whole. The project group was present as well.

The meeting was divided into two parts.

In the first part of the meeting, the way in which the national framework should be used for individual patients with ABI and their families in rehabilitation treatment was discussed. The aim of the discussion was to reach consensus regarding the best suitable and discipline-specific techniques for selecting assessments and interventions for an individual patient with ABI in clinical practice, within the national framework.

In the second part of the consensus meeting, the concept framework was discussed. Participants voted for acceptance/rejection per assessment/intervention that had received 25–75% answers of yes. Again, ≥75% agreement among the representative physiatrists and healthcare professionals was used to include an assessment/intervention. Less than 75% agreement meant no consensus was reached and, therefore, the assessment/intervention would not be included in the national framework*.* Thereafter, the list of assessments and interventions that were already accepted in the second Delphi round (i.e., with more than 75% answering ‘yes’) were presented, and the participants had the opportunity to discuss these items prior to final acceptance.

The list of PE-materials was proposed as well, for a final check of completeness.

The concept framework for the discussion in the third round contained the items that were agreed upon during the second round (assessments/interventions/PE-material with ≥75% ‘yes’) and the items that had to be discussed were highlighted (items with 25–75% ‘yes’). After the consensus meeting, the project group made a final list of assessments and interventions per discipline as well as PE-materials (generic) that reached consensus in the Delphi process.

### Analyses

All analyses were done using SPSS (IBM SPSS Statistics for Mac, version 28, Armonk, NY: IBM Corp). Descriptive statistics were used for the characteristics of the participants. Descriptive statistics were used to present responses from the first round and were expressed as numbers (n) and percentages (%). The dichotomous (yes/no) answers in the second round and the final accepted items in the third round are presented as numbers and frequencies.

## Results

**Figure 1.**
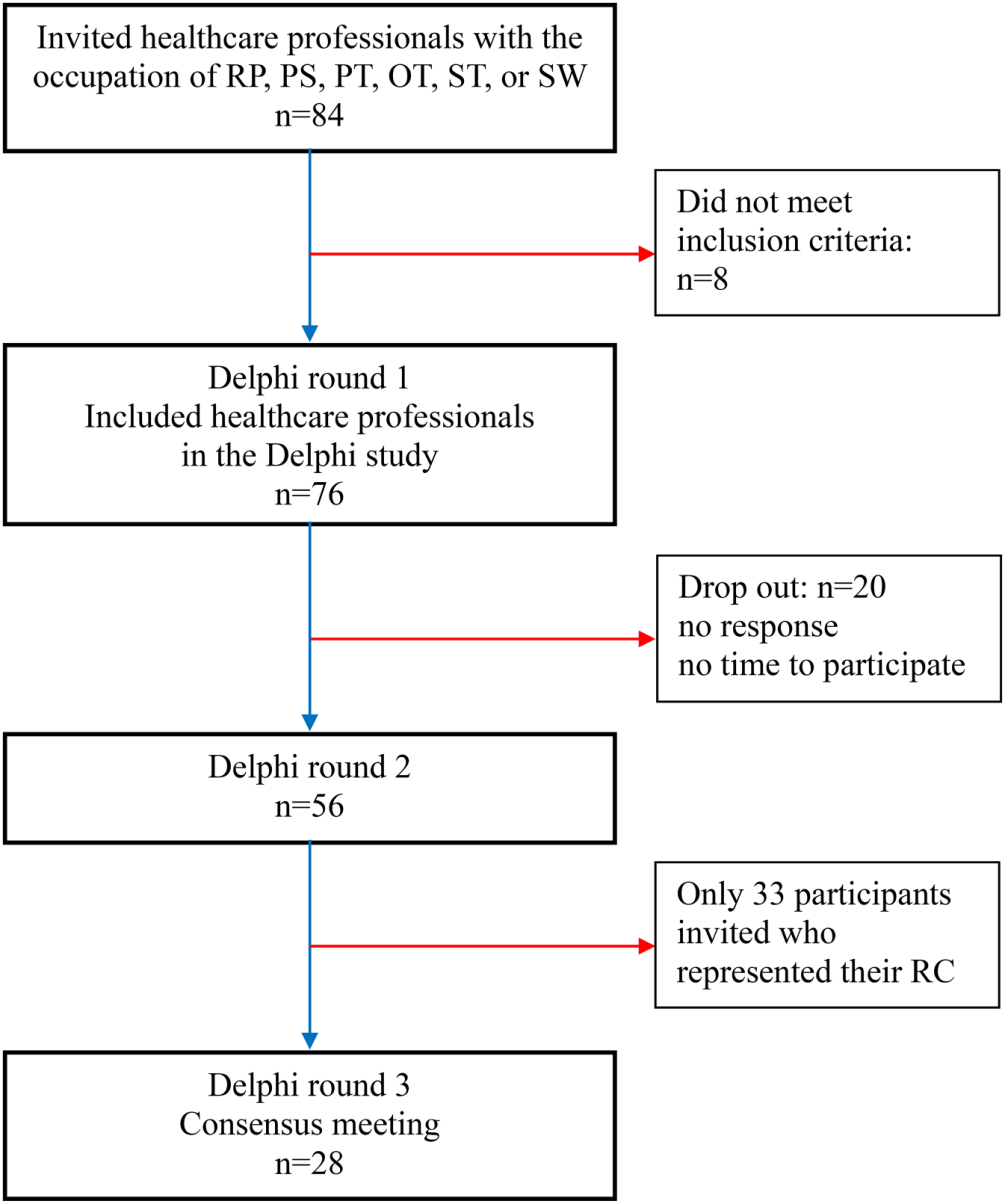
Flow diagram of participants in the Delphi study on assessments, interventions, and psychoeducation materials used in outpatient rehabilitation treatment of young patients with ABI. RP: Rehabilitation physiatrists, PS: Psychologists, PT: Physical Therapists, OT: Occupational Therapists, ST: Speech Therapists, SW: Social Workers.

From 14 RCs in the Netherlands (see Supplementary Material 1), 84 healthcare professionals (12 physiatrists, 15 psychologists, 12 PTs, 20 OTs, 13 SLTs and 12 SWs) were invited to participate. Of those, 76 (90%) responded that they were willing to participate in the study and completed the first round. The flow of included participants in this study is presented in Figure 1. Table 1 shows the characteristics of the participants. Eleven physiatrists (14.5%), 15 psychologists (19.7%), 10 PTs (13.2%), 19 OTs (25.0%), 12 SLTs (15.8%), and nine SWs (11.8%) participated. In the second round, 56 participants responded (74% of 76 responders in total). Finally, n = 33 attended the in-person consensus meeting for the third Delphi round, consisting of 11 physiatrists and 17 other healthcare professionals representing their RC (n = 28) and the project group (n = 5).

**Table 1.** Characteristics of participating health care professionals in the three-round Delphi study.

Characteristics of participants n = 76	Number (%)
Rehabilitation center, n (%)	
Adelante, Valkenburg	4 (5.3%)
Basalt, The Hague	9 (11.8%)
de Hoogstraat, Utrecht	5 (6.5%)
Heliomare, Wijk aan Zee	4 (5.3%)
Klimmendaal, Apeldoorn	8 (10.6%)
Libra, Eindhoven	4 (5.3%)
Merem, Hilversum	5 (6.5%)
Reade, Amsterdam	5 (6.6%)
Revalidatie Friesland, Beetsterzwaag	8 (10.6%)
Revant, Breda	9 (11.8%)
Roessingh, Enschede	5 (6.5%)
Vogellanden, Zwolle	8 (10.6%)
Rijndam, Rotterdam	1 (1.3%)
Universitair Medisch Centrum Groningen/Beatrixoord	1 (1.3%)
Discipline, n (%)	
Physiatrists	11 (14.5%)
Psychologists	15 (19.7%)
Physical therapists	10 (13.2%)
Occupational therapists	19 (25.0%)
Speech language therapists	12 (15.8%)
Social workers	9 (11.8%)
Years of working experience with the target group^a^, n (%)	
<5 years	23 (30.3%)
>5 years	53 (69.7%)

^a^ The cutoff point of <5/≥5 years was agreed upon by the authors and the advisory group.

### First and second online Delphi rounds

After the first Delphi round, a total of 136 unique assessments were listed. During the first Delphi round, the psychologists, representing all participating RCs, proposed a battery for neuropsychological testing, which was listed throughout the Delphi rounds as one assessment. Fifty-one assessments were considered to be related to the field of PT, 45 for OT, 38 for SLT, and two for SW (Table 2). Concerning the interventions, 39 were listed after the first round: nine for psychology, eight for PT, 13 for OT, six for SLT and five for SW (Table 3). Twenty-seven PE-materials were collected and included in the list (Table 4).

**Table 2.** Assessments per ICF domain after the three-round Delphi study among healthcare professionals from 14 Dutch rehabilitation centers.

Discipline	Delphi round 1	Delphi round 2	Result after consensus meeting		ICF (sub)domain			
			Accepted assessment		**b**	**d**	**e**	**s**
Psychology	n = 1	n = 1	n = 1	Battery for neuropsychological testing ^a^	b1	d1/d2	e3/e4	s1
Physical therapy	n = 51	n = 17	n = 9^b^	Two-point discrimination test	b256/b280			
				Six-minute walking test	b450	d420/d450		s770/s730
				Standaard lichamelijk onderzoek ^a^	b735	d420		s730/s730
				Gait analysis		d420/d450		s770/s730
				Acquired Brain Injury Challenge Assessment	b450/b7300	d420/d450		s770/s730
				Visual Analogue Scale	b280			
				Shuttle run test	b450/b740	d420/d450		s770/s730
				Hand-held Dynamometer	b7300/b740			
				Functional Strength Measurement	b450/b7300	d420/d450		s770/s730
Occupational therapy	n = 45	n = 10	n = 10	“Systematische Opsporing Schrijfproblemen” writing test ^a^	b147/b760	d440		s750
				Jamar meter / pinch meter	b7300	d440		s750
				Nine Hole Peg Test	b147/b760	d440		s750
				Assisting Hand Assessment	b147/b760	d440		s750
				“Activiteitenweger” ^a^		d2303		
				Sensory Profile		d2303/d710-d779	e310-e399	
				Daily activities observation list ^a^		d2303/d710-d779	e310-e399	
				Canadian Occupational Performance Measure		d2303/d710-d779	e310-e399	
				Perceive, Recall, Plan Perform	b147	d2303/d710-d779	e310-e399	
				The Beery-Buktenica Developmental Test of Visual-Motor Integration, 6th Edition	b147	d2303		s750
Speech therapy	n = 38	n = 15	n = 15	“Nederlandstalig Dysartrieonderzoek – Kinderen ^a^	b167/b310-b330			
				Token Test	b167/b310-b330			
				Peabody Picture Vocabulary Test	b167/b310-b330			
				Clinical Evaluation of Language Fundamentals	b167/b310-b330			
				Schlichting test ^a^	b167/b310-b330	d330		s310-s340
				Computer-Based Instrument for Low Motor Language Testing	b167/b310-b330	d330		
				Boston naming Task	b167/b310-b330	d330		
				Renfrew Expressive Vocabulary Test	b167/b310-b330	d330		
				Analysis of spontaneous language production	b167/b310-b330	d330	e310-e399	s310-s340
				90 ml swallow test		d330/d550-d560		s310-s340
				Cervical auscultation ^a^		d330/d550-d560		s310-s340
				The Radboud Dysarthria Assessment		d330/d550-d560		s310-s340
				Sunnybrook		d330/d550-d560		s310-s340
				Drooling quotient		d330/d550-d560		s310-s340
				Diagnostic instrument for apraxia ^a^	b167/b310-b330	d330		s310-s340
Social work	n = 2	n = 2	n = 2	Family Questionnaire ^a^		d710-d799	e310-e399	
				Questionnaire focused on burden of care ^a^		d710-d799	e310-e399	
TOTAL	n = 136	n = 45	n = 37					

^a^ Outcome measure only available and/or only developed in Dutch.

^b^ Additional physical therapy assessments (n = 6) that can be used as alternatives for the accepted assessments: Medical Research Council (MRC)-scale test, Functionele spierkracht test *, Steep Ramp Test, Bruce test, Movement-ABC-2 Test, and Gross motor function measure (GMFM).

Abbreviations: body functions (b); activities and participation (d); environmental factors (e); body structures (s); International Classification of Functioning, Disability and Health (ICF).

**Table 3.** Interventions per ICF domain after the three-round Delphi study among healthcare professionals from 14 Dutch rehabilitation centers.

Discipline	Delphi round 1	Delphi round 2	Result after consensus meeting		ICF (sub)domain			
			Accepted intervention		**b**	**d**	**e**	**s**
Psychology	n = 9	n = 7	n = 5	Cognitive behavior Therapy		d250		
				Eye Movement Desensitization & Reprocessing		d250		
				Family meetings			e310-e399	
				Acceptance & Commitment Therapy ^a^	b1	d160-d179	e310-e399	s110
				Strategy training ^b^	b1	d160-d179		s110
Physical therapy	n = 8	n = 8	n = 6	Graded activity / graded exposure ^a^	b740		e3/e4	
				Fitness training	b450/b740	d450		s730/s770
				Functional training	b450/b740	d420/d450		s730/s770
				Mindfulness	b735			
				Training through the “frequency, intensity, time, and type”-factors	b450/b740/b7300	d420/d450		s730/s770
				Advice regarding sports			e3/e4	
Occupational therapy	n = 13	n = 8	n = 7	Strategy training ^b^	b1/b147	d160-d179		s110
				Wheelchair training ^b^	b147/b740/b760	d440		s750
				Graded activity /graded exposure ^a^	b740		e3/e4	
				Constrained- Induced Movement Therapy	b147/b760	d2303/d440/d710-d779	e330-e399	s750
				Independence training ^b^			e330-e399	
				Niet Rennen Maar Plannen ^b^		d2303/d440/d710-d779		
				Errorless learning method		d2303/d440/d710-d779		
Speech therapy	n = 6	n = 6	n = 4	Prompts Restructuring Oral Muscular Phonetic Targets	b167/b330	d330		s310-s340
				Language therapy^b^	b167/b310/b330	d330		s310-s340
				Assistive communication training^b^	b167/b310/b330	d330	e330-e399	
				Logo Art Online	b167/b330	d330		
Social work	n = 5	n = 5	n = 3	Family meetings		d710-d799	e310-e399	
				Acceptance and Commitment Therapy^a^		d710-d799	e310-e399	
				Therapy focused on the whole social system^b^		d710-d799	e310-e399	
TOTAL	n = 39	n = 34	n = 25					

^a^ Test applicable for multiple disciplines.

^b^ Intervention only available and/or only developed in Dutch.

Abbreviations: body functions (b); activities and participation (d); environmental factors (e); body structures (s); International Classification of Functioning, Disability and Health (ICF).

**Table 4.** Psychoeducational materials after the three-round Delphi study among healthcare professionals from 14 Dutch rehabilitation centers.

Result after consensus meeting		
Accepted psychoeducation		
Total number	Specification of type	Name/title
n = 27	Book n = 13	“Ik hou nog steeds van appeltaart” ^a^
		“Brainstars” ^a^
		“Speels brein” ^a^
		“Mag ik ook ff” ^a^
		“NAH niet altijd handig” ^a^
		“Waarom heeft een krokodil zo’n platte kop” ^a^
		“Elvin het vergeetachtige olifantje” ^a^
		“Er lijkt niets met ons aan de hand maar dat is niet zo. Ons hoofd moet heel hard werken” ^a^
		“De puzzel van nah” ^a^
		“Bordje vol” ^a^
		“Omgaan met hersenletsel” ^a^
		“De Zorgzame Giraffe, autobiografisch verhaal over Niet Aangeboren Hersenletsel” ^a^
		“Volle Hoofden Boek (werkboek voor kinderen/ jongeren)” ^a^
	Folder n = 4	“Hoe verder na traumatisch hersenletsel bij kinderen en jongeren” ^a^
		“Slaaptips voor kinderen en pubers” ^a^
		“Het NAH boekje voor onderwijs” ^a^
		Brains ahead! study
	Internet site n = 7	Breinstraat.nl ^a^
		hersenletseluitleg.nl ^a^
		Kinderneurologie.eu ^a^
		Overprikkeling.com ^a^
		“Afasienet.com” ^a^
		“Brain Blocks”
		“Methode RIK (Revalidatie En Ik)” ^a^
	Movie n = 1	“Ze zeggen dat ik zo veranderd ben” ^a^
	Standard of care n = 1	Traumatisch Hersenletsel Kinderen & Jongeren ^a^
	Application n = 1	Energie/activiteitenweger ^a^

^a^ Psychoeducation only available and/or only developed in Dutch.

For the second Delphi round, the number of assessments narrowed down from 136 to 45 and interventions from 39 to 34; PE-materials remained at 27.

### Consensus meeting (third Delphi round)

In the first part of the meeting, consensus was reached on the underlining importance of working in a multidisciplinary and interdisciplinary team due to the heterogeneity and complexity of the target group in which the expertise of each discipline complements the other. For example, PTs and OTs could combine their expertise when using an intervention to enhance the best possible care for an individual. Consensus was also reached on how to select appropriate assessments and interventions from the framework to use with the individual patient. A majority of participants (>75%) agreed that clinical reasoning was important when selecting assessments and interventions for individual patients. Participants suggested adjusting a previously developed flowchart (Swinkels et al.) for facilitating the selection of the most appropriate assessments and interventions from the framework to be suitable for the individual patient with ABI.

**Figure 2.**
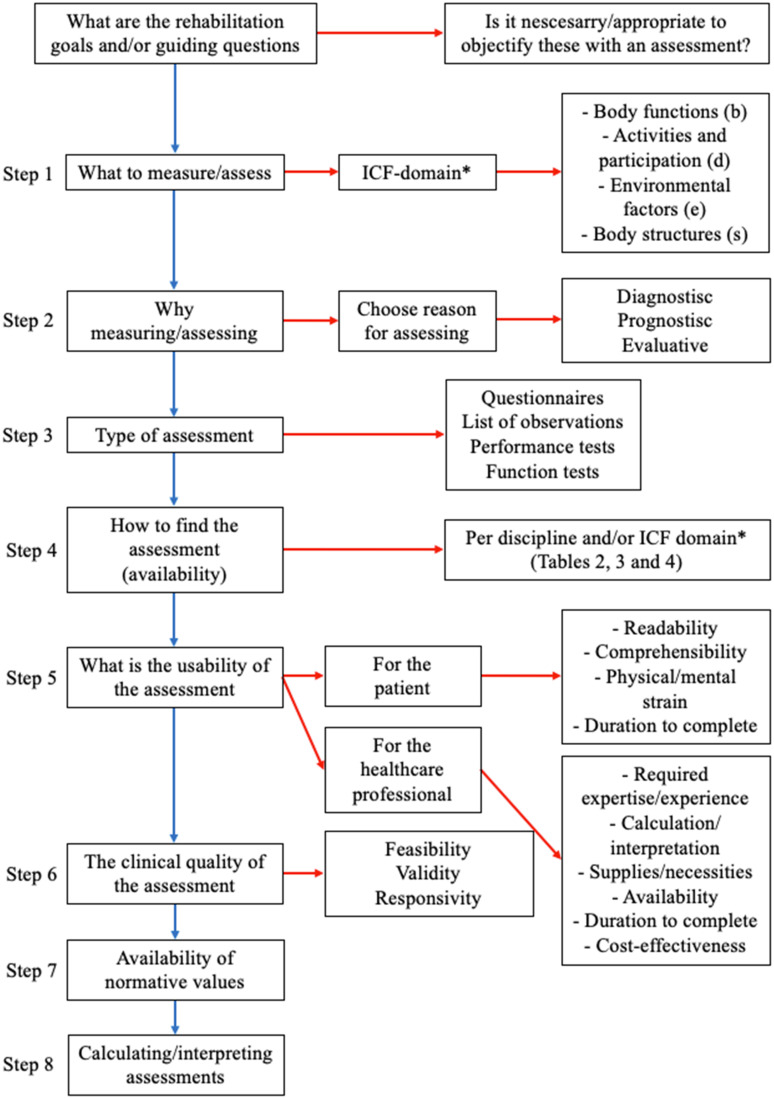
Flowchart for selecting appropriate assessments in clinical practice from the national consensus-based framework. Based on: “Raamwerk klinimetrie voor evidence-based products”, Swinkels et al. 2016.^33^ International Classification of Functioning, Disability and Health (ICF).

After the consensus meeting the project group developed this flow chart (Figure 2), which participants approved (by e-mail).

During the second part of the meeting, consensus was reached on a list of 37 assessments to be included in the national consensus-based framework across the disciplines: nine for PT, 10 for OT, 15 for SLT, and two for SW. The psychologists present during the meeting confirmed the battery for neuropsychological testing was to be listed as one assessment in the national framework.

Furthermore, consensus was reached on a total of 25 interventions: five for psychology, six for PT, seven for OT, four for SLT, and three for SW. The listed assessments and interventions corresponded with all ICF domains including body functions and structures (n = 25 assessments, n = 15 interventions), activities and participation (n = 30 assessments, n = 19 interventions), environmental factors (n = 8 assessments, n = 11 interventions), and body structures (n = 20 assessments, n = 10 interventions).

Finally, all listed PE-materials were confirmed by the group and included in the national framework.

All assessments, interventions, and PE-materials that were confirmed during the consensus meeting were added and merged by the project group to create the national consensus-based framework. Approximately two months after the meeting, the framework was sent to the participating physiatrists and healthcare professionals for a final check. This did not result in any alterations to the list.

See Tables 2–4 for the list of all accepted assessments, interventions, and PE-materials in the national consensus-based framework. The finalized framework can be found in Supplementary Material 2, which is only available in Dutch.

## Discussion

In the current study, the process of developing a national consensus-based framework on preferred assessments, interventions, and PE-materials for young patients with ABI (4–25 years old) and their families was described. This is the first known study to describe the consensus-building process on a national scale across physiatrists and healthcare professionals to optimize and standardize rehabilitation treatment for the pediatric ABI population.

Prior to the consensus meeting of this study, 136 different assessments and 39 interventions were used in the rehabilitation treatment of young patients with ABI and their families in the Netherlands, many of which were used by only a few healthcare professionals across RCs. Many of the assessments and interventions were generic and not specifically developed for the target group. To address this, assessments were needed to identify the specific ICF domains where daily life challenges occurred.^31^ Selecting the most appropriate tools to measure treatment outcomes related to these challenges can help tailor care for young patients with ABI.

In terms of assessments in the field of psychology, only the battery for neuropsychological testing (in Dutch: neuropsychologisch onderzoek [NPO]) was proposed in the Delphi rounds by the participating psychologists. A national consortium of psychologists and physiatrists had already reached consensus on the use of this testing battery which assesses cognitive and mental functioning of young patients with ABI in rehabilitation. This test battery was also the only specific assessment that was described and recommended in the Dutch standard of care.^15^

Through the Delphi study, consensus was reached on 37 assessments that covered all domains of the ICF model.^31^ It is expected that this set is suitable for measuring a broad range of possible daily life problems and patient functioning and evaluating intervention outcomes in the ABI patient population. Many of these listed assessments were psychometrically tested and used among young patients with a wide variety of diagnoses in general pediatric rehabilitation.^16,17^ However, most assessments were not psychometrically tested for the specific pediatric ABI patient population in rehabilitation. Nevertheless, a consensus-based framework of assessments can be used as a tool to potentially diminish practice variation and to help healthcare professionals with selecting the best suitable assessments for the target group. With confirmation of all participating Dutch RCs, this framework will be used in the future, providing the opportunity to gather evidence on the use of the assessments not specifically designed for ABI.

In line with the assessments, interventions focusing on ABI-related consequences that align with diagnosis- and age-specific treatment are crucial for effective rehabilitation treatment.^13,21–25^ The use of evidence-based interventions by healthcare professionals in various patient groups, including children with moderate/severe TBI and adult stroke, has been documented in the literature (e.g., cognitive behavior therapy, graded activity training, and the ABI Challenge Assessment).^13,23,25^ Prior to the current study, healthcare professionals used a wide variety of treatment interventions, and a large variation was seen across RCs in the Netherlands. The Delphi study resulted in a consensus on 25 interventions that covered the whole range of ICF domains.^31^ Consequently, future research should investigate the optimal fit of currently proposed interventions for patients with specific ABI-related problems (e.g., cognitive fatigue, participation restrictions or social/emotional problems) and in specific age groups (e.g., adolescents that are in transition from childhood to adulthood).

The benefits of PE have been emphasized in earlier research and standards of care as being important to help young patients and their families to optimize functioning in daily life by better understanding the sequelae of ABI.^15,26,31^ PE is known to be effective before and during rehabilitation treatment for patients and their parents by enhancing knowledge about brain injury.^10^ The Delphi study identified a list of PE-materials that can be used in rehabilitation treatment. Nevertheless, many of these materials were not specifically developed for the population of young people with ABI and their families. Additionally, a few of the PE-materials on the list included movies, apps, and websites, all of which are inherently transient and subject to change. It is crucial to continue developing and editing this list of materials in accordance with new insights into recovery and functioning of young people after ABI.

### Recommendations and steps to be taken

To provide consistency in rehabilitation treatment across RCs in the Netherlands, consensus was reached on the implementation process of this national consensus-based framework by all the participating RCs (with their teams of physiatrists and healthcare professionals). This is in line with the principles of VBHC.^27^

It is recommended that this framework is used as a tool during rehabilitation treatment to select appropriate assessments in clinical practice, and this was partly based on the flowchart by Swinkels et al.^33^ This clinical reasoning process, as described in Figure 2, is a crucial step for healthcare professionals to select the most suitable items for the individual patient.

Another recommendation arising from this Delphi study is that all disciplines involved during rehabilitation treatment should work together and look further than their own discipline to optimize the best possible multidisciplinary care for the young patient with ABI.

In line with VBHC principles^27^ and literature in pediatric cerebral palsy rehabilitation,^18^ a final recommendation is that the needs, wishes, and goals of individual patients with ABI, and their families, are important to consider when using this national consensus-based document.

Future research and development should focus on gathering evidence about the listed assessments, interventions, and PE-materials (in terms of psychometric properties and effectiveness) to make the consensus-based national framework more evidence-based.

After developing this framework, the next step is implementation in all participating RCs that committed to the “Participate?! Next Step” project. Evaluating and optimizing this framework together with these centers will then be the next important step.

### Limitations

This study had a number of limitations. First, not all Dutch RCs providing rehabilitation treatment for young people with ABI participated in either the “Participate?! Next Step” project or the current Delphi study (2 out of 16 in total), which may have resulted in an incomplete picture/missed assessments, interventions, and PE-materials.

Secondly, most of the results of the Delphi study were applicable to the age group of 4–18 years. Only a few RCs that participated in the current study have a separate transition outpatient clinic through to 25 years, in which the transitions from childhood and adolescence to adulthood get specific attention. Assessments, interventions, and PE-materials specifically for the age group of 18–25 years should be explored further, in line with recommendations to focus on age-appropriate care.^15,34^

Third, the care pathways, methods, and treatment offered in healthcare differs between countries, making the results of this study less generalizable to ABI populations in other countries. Nevertheless, the outlines, procedures, recommendations, and limitations from the current study could be an example for similar research in other countries.

Fourth, at centers where no physiatrist participated in the Delphi rounds, there was no physiatrist involvement in reviewing or vetting the list of assessments, interventions, or PE-materials selected by allied health professionals. Future research should ensure participation of physiatrists from all centers to support interdisciplinary consensus.

Finally, when collecting assessments, interventions, and PE-materials for this framework, only healthcare professionals participated. In line with VBHC principles,^27^ perspectives of patients and their parents on the content of rehabilitation treatment would also be important to take into account when optimizing the current national consensus-based framework.

## Conclusion

This study developed a national consensus-based framework with preferred assessments, interventions, and PE-materials in outpatient rehabilitation treatment of young patients with ABI and their families in the Netherlands. This provides a valuable contribution to optimizing the care and support for these patients and their families. The framework can be used in clinical practice as a tool to enhance selecting appropriate assessments and setting goals before, during, and after outpatient rehabilitation. The consensus-building process described in this study can be used as a blueprint by other research groups to create similar frameworks for other diagnoses. Future research should focus on substantiating and improving the current practice-based national framework into an evidence-based guideline in terms of psychometric properties and effectiveness of the listed assessments, interventions, and PE-materials for the pediatric ABI population.

## Supplemental Material

sj-docx-1-prm-10.1177_18758894251337581 - Supplemental material for A national consensus-based framework on preferred assessments and interventions in current treatment for young people with acquired brain injury in Dutch rehabilitation centers Supplemental material, sj-docx-1-prm-10.1177_18758894251337581 for A national consensus-based framework on preferred assessments and interventions in current treatment for young people with acquired brain injury in Dutch rehabilitation centers by Florian Allonsius, Arend de Kloet, Frederike van Markus-Doornbosch, Ingrid Rentinck, Suzanne Lambregts, Karin Huizing, Peter de Koning, Sandra te Winkel, Christine Resch, Thea Vliet Vlieland and Menno van der Holst in Journal of Pediatric Rehabilitation Medicine

sj-pdf-2-prm-10.1177_18758894251337581 - Supplemental material for A national consensus-based framework on preferred assessments and interventions in current treatment for young people with acquired brain injury in Dutch rehabilitation centers Supplemental material, sj-pdf-2-prm-10.1177_18758894251337581 for A national consensus-based framework on preferred assessments and interventions in current treatment for young people with acquired brain injury in Dutch rehabilitation centers by Florian Allonsius, Arend de Kloet, Frederike van Markus-Doornbosch, Ingrid Rentinck, Suzanne Lambregts, Karin Huizing, Peter de Koning, Sandra te Winkel, Christine Resch, Thea Vliet Vlieland and Menno van der Holst in Journal of Pediatric Rehabilitation Medicine
