# Supplementary material for: A national consensus-based framework on preferred assessments and interventions in current treatment for young people with acquired brain injury in Dutch rehabilitation centers
Source: J Pediatr Rehabil Med. 2025 Apr 23:18758894251337581. Online ahead of print. doi: 10.1177/18758894251337581 (PMC13292738; doi:10.1177/18758894251337581)
Supplement: Supplementary file 1 [file sj-pdf-2-prm-10.1177_18758894251337581.pdf]

MEDISCH SPECIALISTISCHE REVALIDATIE

BEHANDELPROGRAMMA

voor kinderen en jongvolwassenen met niet-aangeboren hersenletsel

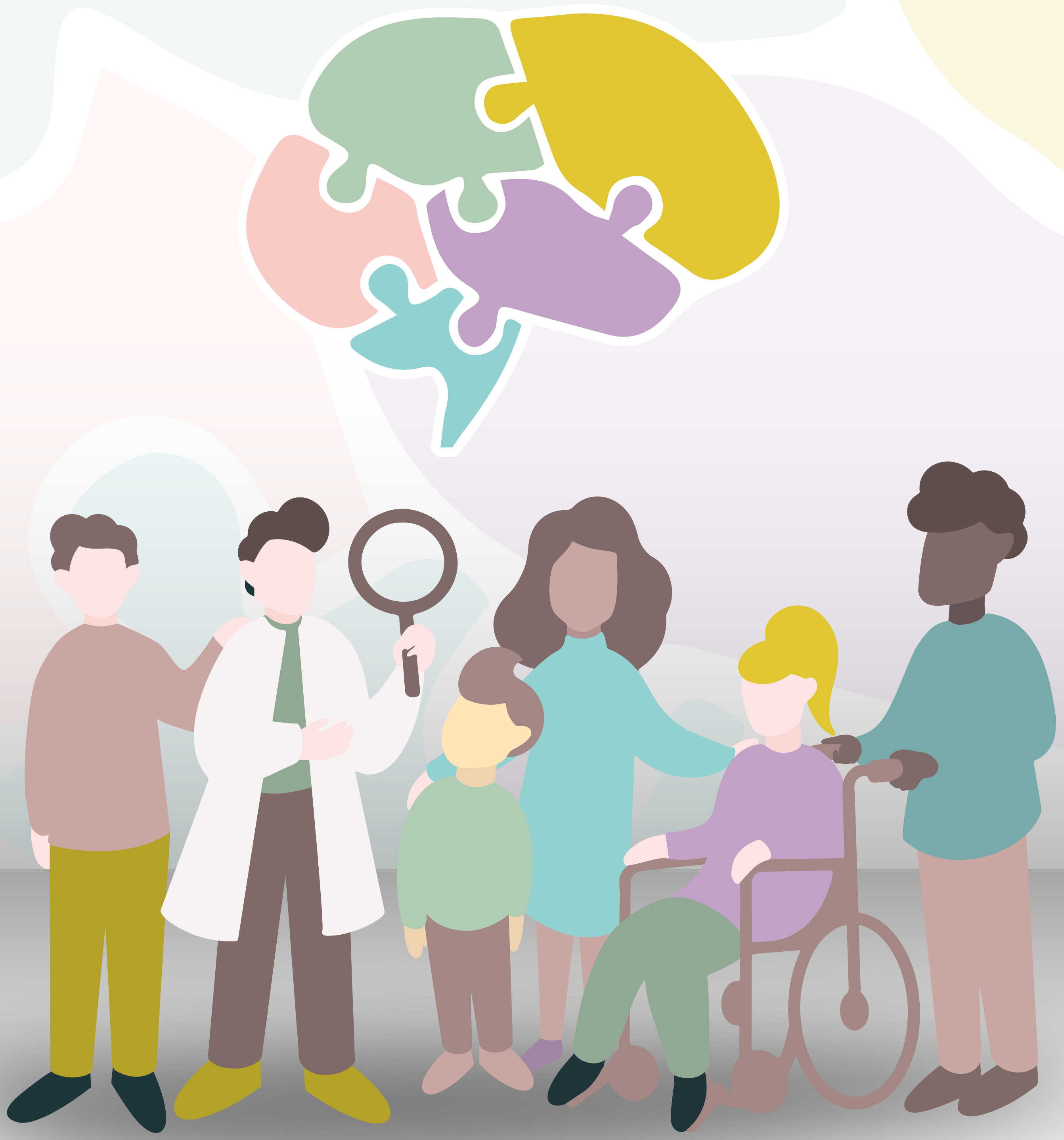

Auteurs:
Florian Allonsius (onderzoeker/PhD-student, Basalt)
dr. Arend de Kloet (projectleider, Basalt)
dr. Frederike van Markus-Doornbosch (senior onderzoeker, Basalt)
dr. Menno van der Holst (projectleider, Basalt)

In samenwerking met 14 deelnemende revalidatiecentra:
Adelante, Basalt Revalidatie, De Hoogstraat Revalidatie, Heliomare Centrum voor Specialistische Revalidatie, Klimmendaal Revalidatiespecialisten, Libra Revalidatie & Audiologie, Merem Medische Revalidatie, Reade, Revant Medisch Specialistische Revalidatie, Revalidatie Friesland, Roessingh Centrum voor Revalidatie, Rijndam Revalidatie, UMCG/Beatrixoord en Vogellanden Centrum voor revalidatie

Met speciale medewerking van:
Eline Lommerse (Adelante), Frederike van Markus-Doornbosch (Basalt), dr. Ingrid Rentinck (de Hoogstraat), Martine Besseling (Heliomare), Suzanne Franssen (Klimmendaal), Martina Quint (Libra), Meike van Ginneken (Libra), Marian Elffers (Merem), Lynnette Spits (Reade), Hans van Herwaarden (Reade), Nicole Bovens (Revant), Maud DeMeester (Revant), Marjan de Vries (Revalidatie Friesland), Marieke Tijdhof (Roessingh), Amanda van Zuijlekom (Rijndam), Nynke van der Meulen (Vogellanden), Ellen Theunissen (Vogellanden)

En met dank aan de stuurgroep:
Peter de Koning
dr. Suzanne Lambregts (Revant)
Karin Huizing (Revalidatie Friesland)
Martine Sinnema (Revalidatie Friesland)
dr. Christine Resch (Universiteit Maastricht)
dr. Ingrid Rentinck (De Hoogstraat)
Sandra te Winkel (Merem)

Gesubsidieerd door:

Hersen Stichting

Layout: www.wenz iD.nl
Versie 1.0 december 2022

INHOUDSOPGAVE

VOORWOORD 4

1. DOELGROEP 6

2. VISIE OP DE BEHANDELING VAN DE DOELGROEP 8

3. BEHANDELPROGRAMMA - PROCES VAN BEHANDELING 12
   3.1. In- en exclusiecriteria 12
   3.2. Aanmelding klinische en poliklinische revalidatie 12
   3.3. Klinische revalidatiebehandeling 13
   3.4. Poliklinische revalidatiebehandeling 14
   3.5. Klinisch redeneren 16

4. BEHANDELPROGRAMMA - DIAGNOSTIEK EN INTERVENTIES 19
   4.1. Psychologie /Orthopedagogiek 20
   4.2. (Kinder)fysiotherapie 29
   4.3. Ergotherapie 52
   4.4. Logopedie 71
   4.5. Maatschappelijk werk 95
   4.6. Andere disciplines 103
   4.7. Materialen voor psycho-educatie 104

5. BEHANDELPROGRAMMA - TRANSITIEFASE 107

6. REGIO-OVERSTIJGENDE, SPECIALISTISCHE CENTRA 109

7. REFERENTIES 112

BIJLAGE: PROCESBESCHRIJVING 118
   1. Totstandkoming van het landelijk behandelprogramma 118
   2. Relatie met Zorgstandaard en Behandelkader 119
   3. Implementatie en evaluatie 120
   4. Landelijke inbedding en borging behandelprogramma 121
   5. Regionale samenwerking 121
   6. Ontwikkeling en Onderzoek 123
   7. Referenties 125

VOORWOORD

Dit behandelprogramma is een beschrijving van het behandelaanbod in de Medisch Specialistische Revalidatie (MSR) voor de diagnosegroep kinderen en jongvolwassenen (hierna 'jongeren' genoemd) met Niet-Aangeboren Hersenletsel (NAH). Het behandelprogramma beslaat het hele revalidatieproces en beschrijft dit op hoofdlijnen. Er wordt beschreven wat de inhoud van de behandeling kan zijn voor de jongere en zijn/haar gezin (psycho-educatie, diagnostiek, interventies; geordend op basis van het ICF-model), hoe dit aangeboden kan worden (logistiek intern, samenwerking regionaal) en door welke disciplines.

Het behandelprogramma is opgesteld in lijn met de Zorgstandaard Traumatisch Hersenletsel (THL) kinderen & jongeren, in 2016 gepubliceerd en in 2022-2023 geactualiseerd,[1] en het 'Behandelkader Revalidatie van jongeren met NAH'', vastgesteld door de Nederlandse Vereniging van Revalidatieartsen (VRA) in 2013. In de zorgstandaard wordt beschreven wat er verwacht mag worden van de zorg voor kinderen en jongeren met traumatisch hersenletsel in Nederland.[1] In het behandelkader worden de minimale eisen beschreven waaraan een behandelteam voor Medisch Specialistische Revalidatie (MSR) in een revalidatiecentrum of ziekenhuis moet voldoen (zie verder blz. 122).

Dit behandelprogramma is gerealiseerd dankzij een subsidie van 'de Hersenstichting'. Bij de totstandkoming van het behandelprogramma hebben onderzoekers van de afdeling Innovation, Quality & Research (IQ+R) van Basalt Revalidatie met nagenoeg alle centra voor MSR samengewerkt. Al deze centra hebben gezamenlijk de inhoud bepaald en ingestemd met de implementatie van het behandelprogramma. Het gebruik van dit behandelprogramma is bindend, waarbij voor alle centra geldt dat er ruimte is voor centrum specifieke of regionale inkleuring waar nodig. Het landelijk behandelprogramma is een dynamisch document, dat regelmatig bijgesteld zal worden waar nodig. Hersenletsel Alliantie is eigenaar, met de werkgroep Hersenletsel en Jeugd (HeJ) als mede-eigenaar en verantwoordelijk voor de borging.In de bijlage wordt beschreven hoe het behandelprogramma tot stand gekomen is en hoe dit geïmplementeerd en geborgd wordt (procesbeschrijving).

1

1. DOELGROEP

Het behandelprogramma is gericht op alle jongeren (4-18 jaar) met NAH en hun gezin die zijn verwezen naar de MSR voor behandeling. Het doel van deze behandelingen is het bevorderen van participatie door het stimuleren van herstel en het verminderen van de beperkende gevolgen van NAH op de participatie. Dit behandelprogramma gaat niet in op de MSR voor de leeftijdsgroep 0-4 jaar. In de meeste centra voor MSR wordt deze leeftijdsgroep behandeld door teams gespecialiseerd in het jonge kind met een ontwikkelingsstoornis. Het kan wel gebruikt worden voor jongvolwassenen (19-25 jaar). In Nederland wordt deze leeftijdsgroep soms bij de volwassen revalidatie behandeld en soms bij de kinderrevalidatie. In vijf centra voor MSR (Rijndam, Basalt, Reade, Libra, de Hoogstraat) is een specifiek aanbod voor deze leeftijdsgroep (14 of 18-25 jaar) ontwikkeld (zie hoofdstuk 5).

Definitie niet-aangeboren hersenletsel (NAH)
NAH is een verzamelnaam voor letsel aan de hersenen dat ontstaan is na de geboorte wat leidt tot een duidelijke breuk in de ontwikkelingslijn (levenslijn) van het kind of de jongere. [1,2]

NAH kan ontstaan door:
a) Een externe oorzaak, dat wil zeggen buiten het lichaam, zoals een klap tegen het hoofd (bijvoorbeeld na een ongeval) of acceleratie-deceleratie (doordat het hoofd onverwacht en snel heen en weer geschud wordt). Dit wordt omschreven als traumatisch hersenletsel (THL).[2-4] Deze omschrijving is ook in lijn met de internationale richtlijnen licht THL.
b) Een hersenaandoening (bijvoorbeeld een hersenbloeding of hersentumor), heeft een interne oorzaak en wordt omschreven als niet-traumatisch hersenletsel (nTHL).[2-4]

De ernst van het traumatisch letsel, wordt ingedeeld in licht, matig of ernstig op basis van de Glasgow Coma Scale (GCS) en andere acute symptomen.[5] De ernst van het letsel heeft niet altijd een directe voorspellende relatie met de ernst van de symptomen en gevolgen voor de jongere.[1,2]
Voor het classificeren van de ernst van nTHL is er momenteel, vanwege de complexiteit en verscheidenheid aan oorzaken en het soms grillige en onvoorspelbare verloop, geen eenduidige classificatie beschikbaar.

2

2. VISIE OP DE BEHANDELING VAN DE DOELGROEP

De doelgroep jongeren met NAH is zeer divers. Door grote verschillen in het letsel (type, oorzaak en ernst), persoonlijke- (b.v. leeftijd, pre-morbide problemen) en omgevingsfactoren (b.v. gezinskenmerken), wordt het behandelprogramma zoveel mogelijk op maat aangeboden. Het aanbod in de MSR wordt toegespitst op de levensfase van de jongere en groeit mee met de wensen en behoeften van de jongere en het gezin. Voor jongeren, het gezin en de sociale omgeving is voorlichting een essentieel onderdeel gedurende de hele behandeling. Verschillende disciplines kunnen worden ingezet om zo goed mogelijk op letsel-, persoonlijke en omgevingsfactoren in te kunnen spelen.

Elke jongere in Nederland met niet-aangeboren hersenletsel (NAH) krijgt behandeling gebaseerd op de onderstaande drie pijlers:

1) De juiste zorg:

- In elke regio in Nederland wordt het landelijke behandelprogramma aangeboden, met advies voor start- en stop moment, inhoud en werkwijze, rekening houdend met individuele risico- en voorspellende factoren.
- Patient Reported Outcome Measures (PROMs) en klinimetrie geven richting aan het behandeltraject en de evaluatie hiervan. De ouder(s) en jongere beschikken zelf direct over de resultaten van de PROMs en hebben de mogelijkheid tot inzage van het eigen dossier en regelmatige afstemming met het behandelteam. ‘Eigen regie’, ‘samen beslissen’ en ‘eigenaarschap’ over de behandeling zijn hierbij belangrijke pijlers.
- Ouders zijn en blijven eindverantwoordelijk voor de gezondheid en het welbevinden van hun kind. (Zorg) Professionals hebben de taak om ouders hiertoe volledige informatie, tools en ondersteuning aan te reiken.

2) Op de juiste plek:

- In elke regio in Nederland is er een revalidatiecentrum of ziekenhuis met een multidisciplinair team dat gericht is op de behandeling van jongeren met NAH (conform beschreven in het ‘Behandelkader Revalidatie van jongeren met NAH’). De behandeling wordt dichtbij huis geboden waar mogelijk en centraal (klinisch of gespecialiseerd centrum) waar nodig.
- Snel en gericht doorverwijzen tussen aanbieders in de regionale netwerkzorg vergroot de efficiëntie, kwaliteit en tevredenheid.

3) Op het juiste moment:

- Er is regionale consensus over wie/ wanneer/ waarnaar toe verwezen kan worden. Dit geldt voor verwijzing naar, maar ook vanuit, de MSR. Bij netwerkgeneeskunde en doorverwijzing liggen problemen op de loer, zoals onduidelijke informatieoverdracht en (opnieuw) wachttijd bij verwijzing, deze problemen zijn oplosbaar en dienen voorkomen te worden. De principes van ‘stepped care’ worden toegepast waar mogelijk.
- In de MSR wordt landelijk gestreefd naar een zo kort mogelijke wachttijd tot het eerste consult bij de revalidatiearts. Indien revalidatiebehandeling geïndiceerd is wordt daarna gestreefd naar een zo kort mogelijk wachttijd tussen het eerste consult en de start van een poliklinische behandeling (zien hoofdstuk 3).[1]

- Het uiteindelijke doel is het vergroten van de participatie door het verbeteren van fysiek, cognitief, communicatief en/of sociaal-emotioneel functioneren. Met participatie wordt bedoeld meedoen en erbij horen in het gezin, op school/opleiding/werk en in het sociaal en burgerlijk leven. Hierbij wordt rekening gehouden met de wensen en mogelijkheden van jongere en omgeving.

Het behandelprogramma MSR wordt **zo dicht mogelijk bij huis en zoveel mogelijk in samenwerking met de eigen sociale omgeving** aangeboden. Ouders en andere gezinsleden worden gezien en betrokken (als partners) in het behandeltraject en krijgen zelf ook de ondersteuning die nodig is. Daar waar nodig wordt de onderwijsinstelling, werkgever en/of andere belangrijke personen (b.v. eerstelijns therapeut, vriend(en)/vriendin(en), familielid/leden) betrokken.

In de herstelfase na **matig/ernstig** NAH is veelal intensieve behandeling in de MSR geïndiceerd om het herstel en functioneren te optimaliseren. In de participatiefase is de zorg voornamelijk gericht op specifieke ontwikkelingsvragen. Iedere ontwikkelingsfase (basisschool, middelbare school, puberteit, transitie naar volwassenheid) heeft zijn eigen uitdagingen passend bij de normale ontwikkeling van kinderen. Vanuit dit perspectief kunnen jongeren en ouders ondersteund worden in de transitie naar volwassenheid. Voor **licht traumatisch hoofdletsel (LTH)** ligt in de eerste drie maanden na het letsel het accent op uitleg (psycho-educatie), geruststelling, 'watchfull waiting' en natuurlijk herstel. De adviezen (doorgaans vanuit ziekenhuis, huisarts) zijn gericht op een opbouw van activiteiten qua frequentie en intensiteit op geleide van belastbaarheid (na maximaal 48 uur rust) eventueel in combinatie met 1e-lijns therapie.[1-3,6] Verwijzing naar de MSR is geïndiceerd bij persisterende klachten die drie maanden of langer bestaan, welke mogelijk een multidisciplinaire aanpak behoeven. Daarbij moet er sprake zijn van een breuk in de ontwikkelingslijn en/ of moeten er beperkingen op activiteiten- en participatieniveau bestaan. In de MSR wordt bij LTH gestart met een goede analyse om de aanhoudende klachten te verklaren.[1-3,6-10] Vanuit de werkgroep revalidatie van HeJ is een advies geformuleerd om bij deze doelgroep een brede analyse te maken van de mogelijke factoren die van invloed zijn op het herstel zodat een overwogen keuze gemaakt kan worden voor passende diagnostiek en/of interventies. Voor meer informatie over dit advies wordt verwezen naar de website van de Hersenletsel Alliantie.

In de behandeling worden **de recente inzichten in neuroplasticiteit en herstel van functies** toegepast: algemene principes voor fysieke revalidatie op gebied van timing ('as soon as possible') en dosering (frequentie, intensiteit en duur), worden afgestemd op de belastbaarheid van de jongere.[11-13] Bewezen interventies zoals 'family centered care' en 'peer-based care' zijn tevens verwerkt in het behandelprogramma.[14,15]

Voor de uitvoering van dit behandelprogramma in de MSR is vereist

- Een deskundig, interdisciplinair werkend, multidisciplinair samengesteld behandelteam.
- Een revalidatiearts deskundig in de behandeling van/zorg voor NAH bij jongeren en jongvolwassenen, die sturing geeft aan bovengenoemd team.
- In het revalidatieteam is deskundigheid op het gebied van spasticiteit, linguïstiek, neurocognitieve revalidatie, (neuro-psychiatrische) gedragsproblemen, seksualiteit, onderwijs, dagbesteding, arbeid en participatie in sport/ vrije tijdsbesteding en systeemproblematiek bij jongeren of er is nauwe samenwerking mogelijk met externe professionals op deze gebieden.

Voor klinische revalidatiebehandeling geldt ook als vereiste dat het verpleegkundig team deskundig is en ervaring heeft op het gebied van de zorg voor een jongere met NAH en er continuïteit is in de verpleegkundige zorg. In de subacute fase: specifieke kennis en ervaring met preventie van secundaire complicaties, prognostiek en triage naar vervolgsetting. Voor het kunnen bieden van een therapeutisch klimaat op de afdeling is een op de problematiek van de jongere aangepaste huiskamer wenselijk met de mogelijkheid voor intensieve 1-op-1 begeleiding.

Minimaal beschikbare disciplines (zoals ook omschreven staat in het Behandelkader Revalidatie van jongeren met NAH van de VRA): Revalidatiearts, GZ-psycholoog, psychologisch medewerker, cognitief revalidatietherapeut, fysiotherapeut, ergotherapeut, logopedist, bewegingsagoog, maatschappelijk werkende, pedagogisch medewerker, verpleegkundige (klinische revalidatie), diëtist.

Minimaal opleidings- en kennisadvies

- Behandelaren in de MSR zijn geschoold op het gebied van motorische, cognitieve, communicatieve, emotionele en gedragsproblemen bij jongeren met NAH in verschillende ontwikkelingsfasen. Ook ten aanzien van transities en de belevingswereld en interesses van jongeren dienen zij kennis te hebben. Behandelaren beschikken over voldoende sociale en communicatieve vaardigheden om een optimale samenwerking met de jongere en zijn/haar omgeving te bewerkstelligen. Ook zijn de principes van recente inzichten in neuroplasticiteit (b.v. leerprincipes, training, trainbaarheid) bij hen bekend.[11-13]
- Kennis van richtlijnen op het gebied van NAH.[1,16]
- Kennis en vaardigheden gericht op de begeleiding van jongeren met NAH.

Voor de regionale netwerkzorg wordt deskundigheidsbevordering bij voorkeur regionaal georganiseerd, waarbij uitwisseling van producten en ervaringen tussen de regio's door een landelijk consortium wordt gecoördineerd (Hersenletsel Alliantie en Hersenletsel en Jeugd, (HeJ)).

In elke regio bestaan afspraken over de samenwerking tussen netwerkpartners. Het verschilt per regio of en hoe deze afspraken zijn vastgelegd en welke wensen voor verbetering bestaan. Een regionaal convenant tussen netwerkpartners kan helpen om regionale afspraken en ambities vast te leggen, commitment te waarborgen en het proces en de beoogde opbrengsten te faciliteren. Het uiteindelijke doel van het convenant is, dat In elke regio wordt gestreefd naar het bewerkstellingen van 'de beste zorg-op het juiste moment-op de juiste plek' voor de doelgroep.

Op bestuurlijk niveau is per centrum, en liefst ook landelijk, commitment nodig voor het implementeren en borgen van het behandelprogramma en de samenwerking met regionale netwerkpartners.

3

3. BEHANDELPROGRAMMA - PROCES VAN BEHANDELING

3.1. IN- EN EXCLUSIECRITERIA

Een jongere kan profiteren van dit behandelprogramma in de MSR als:

- De diagnose NAH gesteld is of hier een sterk vermoeden van is;
- Er een indicatie is gesteld door de revalidatiearts voor multidisciplinaire revalidatiebehandeling in de MSR;
- Er geen emotionele, gedragsmatige of psychiatrische problemen of psychosociale factoren zijn, die de behandeling te zeer zouden kunnen belemmeren.

Indien er sprake is van zeer complexe problematiek (fysiek, emotioneel, gedragsmatig, psychosociaal), is verwijzing naar het Netwerk Kind & NAH+ passend (zie hoofdstuk 6).

3.2. AANMELDING KLINISCHE EN POLIKLINISCHE REVALIDATIE

De aanmelding voor klinische revalidatie gebeurt op indicatie van de revalidatiearts. De primaire instroom betreftjongeren, die opgenomen zijn in het ziekenhuis en nog niet ontslagen kunnen worden naar huis. In uitzonderlijke situaties kan tijdens het eerste spreekuur voor poliklinische revalidatie besloten worden tot klinische revalidatie. Voor poliklinische revalidatie wordt een jongere na een verwijzing samen met zijn/haar ouders uitgenodigd voor het spreekuur met de revalidatiearts. In sommige centra wordt het spreekuur samen met de (GZ-)psycholoog gedaan. Tijdens het spreekuur wordt de voorgeschiedenis uitgevraagd, de anamnese en heteroanamnese afgenomen.

De revalidatiearts stelt een revalidatiediagnose gebaseerd op anamnese, lichamelijk onderzoek en eventueel aanvullend onderzoek. Er wordt gevraagd naar medicijnengebruik, drugs/alcohol gebruik en allergieën. De hulpvraag van de jongere en ouders wordt uitgevraagd, gebruik makend van de door ouders en jongere (vanaf 8 jaar) ingevulde meetset, die is samengesteld uit Patient Rated Outcome Measures (PROMS; zie hoofdstuk 2).

Tijdens het eerste consult bepaalt de revalidatiearts of er op dat moment een indicatie is voor MSR en onderzoekt samen met jongere en ouders wat de mogelijke vervolgstappen zijn. Als een jongere niet in behandeling komt wordt afgesproken wanneer de jongere weer op het spreekuur komt als hier een indicatie voor is.

Na het consult informeert de revalidatiearts, zoals gebruikelijk, de huisarts en/of de verwijzer. Met toestemming van jongere en/of ouders kunnen ook anderen worden geïnformeerd, zoals de jeugdarts.

3.3. KLINISCHE REVALIDATIEBEHANDELING

De doelgroep voor klinische revalidatie na matig-ernstig of ernstig hersenletsel is relatief klein. Aanvullend op de in- en exclusiecriteria, is klinische revalidatie geïndiceerd als:

- De jongere een beperkte belastbaarheid heeft waardoor hij/zij (nog) niet in staat is om vanuit een poliklinische situatie een intensief revalidatieprogramma te volgen en/of;
- De problematiek op het gebied van motoriek en/of cognitie maken dat er een indicatie is voor een intensief therapeutisch programma dat alleen in een klinische setting te realiseren is en/of;
- De sociale situatie is dusdanig, dat het volgen van een revalidatieprogramma vanuit huis (nog) niet mogelijk is (wel is er zicht op dat dit gerealiseerd kan worden);
- Er sprake is van een niet stabiele medische toestand en/of de verwachting is dat er geen herstel kan plaatsvinden, is klinische revalidatie niet passend.

De overplaatsing vanuit het ziekenhuis naar het revalidatiecentrum kan plaatsvinden als de basis medische situatie stabiel is en als men verwacht dat er herstel kan plaatsvinden op motorisch, cognitief, communicatief en gedragsmatig niveau. Dat een jongere nog in de posttraumatische amnesie (PTA)-fase zit, of nog volledig afhankelijk en/of laag belastbaar is, vormt geen belemmering voor de overgang naar het revalidatiecentrum.

De duur van klinische revalidatie is zeer variabel, mede afhankelijk van het letsel, de herstelcurve, en de gestelde doelen. Insteek van de klinische revalidatie is dat deze alleen geboden wordt indien strikt noodzakelijk. Er wordt verondersteld dat de eigen vertrouwde omgeving een positief effect heeft op herstel. Vanuit de klinische setting wordt stapsgewijs gewerkt aan terugkeer naar, en participatie in, de eigen vertrouwde context.

Het aanbod in de klinische revalidatiebehandeling is erop gericht om basale dagelijkse vaardigheden weer te normaliseren (zo nodig opnieuw te leren) en op te bouwen. Dit gebeurt binnen de context van de afdeling op momenten dat het voor de revalidant logisch is (bv aankleden tijdens de ochtendzorg, overgaan op orale voeding tijdens de eetmomenten, reguleren van een gezond slaap-waakritme).
In het begin van het herstel van ernstig hersenletsel verlopen veel vaardigheden nog niet automatisch. Het opnieuw automatiseren vraagt veel herhaling t.b.v. het inslijpen van vaardigheden.
Door middel van rooming-in van ouders ontstaat de mogelijkheid om hen te leren hoe ze het veranderde gedrag van hun kind kunnen begrijpen en het herstel kunnen faciliteren door een stimulerende benadering bij dagelijkse vaardigheden en door het creëren van extra beweegmomenten.
Klinische revalidatie biedt de mogelijkheid om goed aan te sluiten bij een zeer beperkte belastbaarheid. De jongere kan zich terugtrekken op de eigen kamer op de afdeling voor rust en/of slaap. De therapiemomenten kunnen over de dag verspreid worden.
Door de intensieve interdisciplinaire werkwijze en snelle en directe onderlinge afstemming over benaderingswijze binnen het team en met ouders/verzorgers, kan de behandeling met het vorderen van het herstel snel en direct bijgesteld worden.

3.4. POLIKLINISCHE REVALIDATIEBEHANDELING

In het onderstaande stroomdiagram wordt het proces in de poliklinische MSR stapsgewijs geschetst.

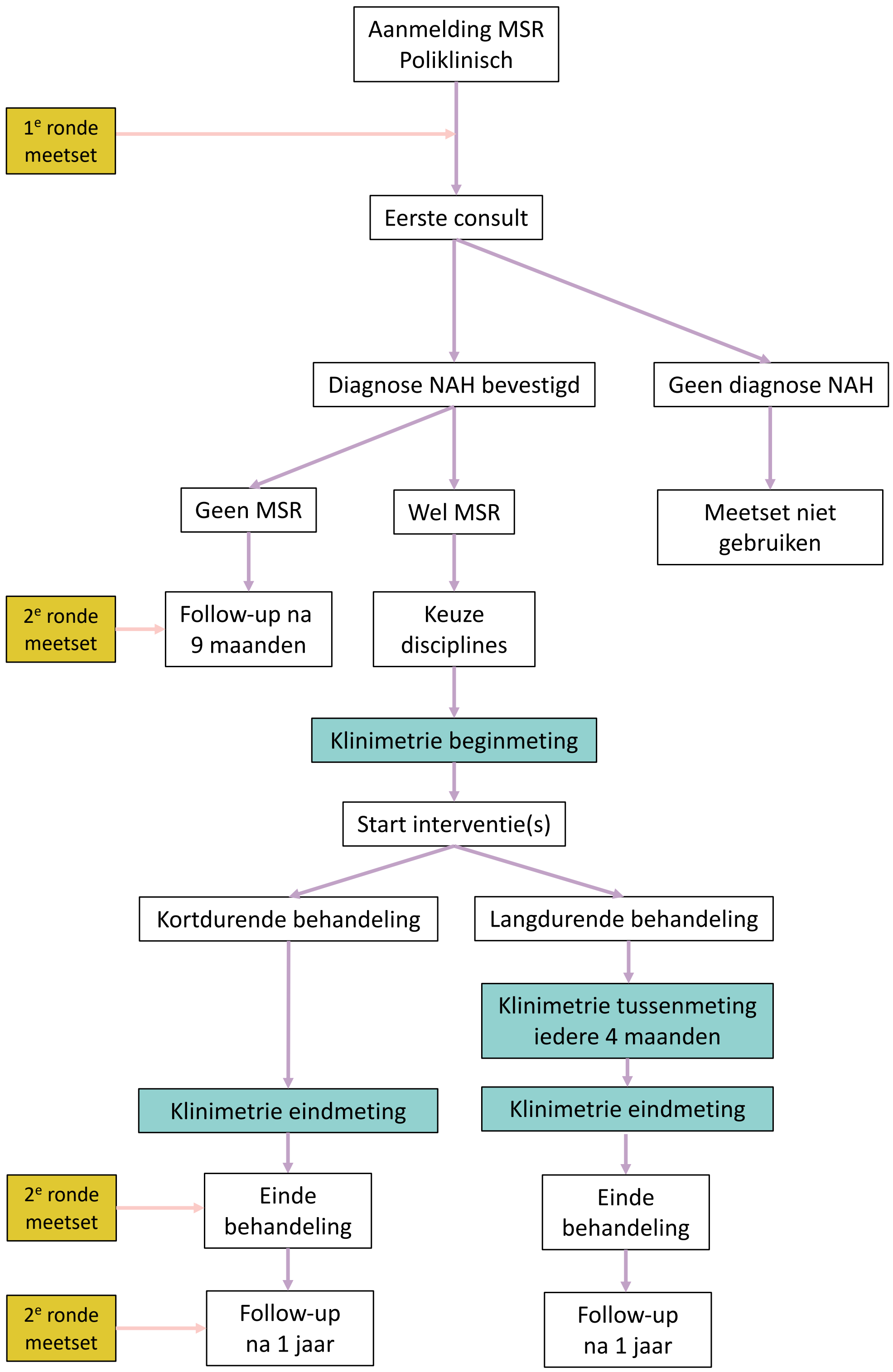

Toelichting stroomdiagram poliklinische revalidatie

Eerste poliklinische consult

Een revalidatiearts, soms met een (neuro)psycholoog of arts-assistent, onderzoekt de vragen en verwachtingen van de jongere en zijn/haar ouders in relatie tot het hersenletsel en maakt hierbij gebruik van de ingevulde vragenlijsten (meetset PROMS). Samen worden de belangrijkste hulpvragen vastgesteld en afgezet tegen een mogelijk behandelaanbod. Eventuele vervolgafspraken worden in overleg en met instemming van de jongere en/of ouders gemaakt: tot de leeftijd van 12 jaar moeten ouders hierin wettelijk toestemming/akkoord geven; met jongeren van 12-16 jaar gebeurt dit met wederzijdse instemming en moeten de ouders wettelijk toestemming geven; vanaf 16 jaar bepaalt de jongere zelf. Als er wordt besloten te starten met een behandeling binnen de MSR wordt een behandelovereenkomst besproken en gemaakt. Naar behoefte wordt al tijdens dit eerste consult psycho-educatie gegeven bv. over beloop van klachten, prognose en eventuele invloed van andere factoren.

Observatie en onderzoeksfase

Vaak wordt er gestart met een intake, observaties en testen door de disciplines die door de revalidatiearts worden ingeschakeld. In deze fase worden de resultaten van de vragenlijsten (meetset PROMS) eveneens benut en eventueel aanvullende psycho-educatie gegeven. Deze observatie- of onderzoeksfase wordt meestal afgerond met een uitslaggesprek met de jongere en ouders, waarin op basis van de resultaten een behandelplan wordt voorgelegd.

Behandelfase

In overleg met jongere en ouders zorgt de planning voor een rooster (dag en tijd, frequentie, discipline) voor zowel de jongere als de ouders, gezin of andere voor de jongere belangrijke betrokkenen. De observaties of testen worden zo nodig tussentijds herhaald. Zo nodig vindt er afstemming plaats met school en/of andere relevante betrokkenen. De behandeling is dynamisch; frequenties en doelen van disciplines kunnen tijdens de behandeling wijzigen. Dit gebeurt steeds in overleg en met instemming van jongere en/of ouders. Ook worden zij bijtijds op de hoogte gesteld wanneer de behandeling afgerond gaat worden.

Afronding

Als een behandeltraject wordt afgerond, worden opnieuw de vragenlijsten (meetset PROMS) door jongere en ouders ingevuld. Voor de evaluatie worden de relevante onderzoeken uit de observatiefase herhaald. De resultaten worden met de jongere en ouders besproken door de revalidatiearts en/of behandelaars. Het kan zijn dat er een afspraak wordt gemaakt om na verloop van tijd opnieuw te bespreken hoe het op dat moment met de jongere en het gezin gaat en of er nieuwe hulpvragen zijn voor de MSR.

Follow-up/nazorg

Bij de nazorg wordt zo nodig een nazorgplan gemaakt ten aanzien van onderwijsondersteuning, gezinsondersteuning (bijvoorbeeld opvoedingsondersteuning en zorg voor broertjes/ zusjes), persoonlijke ondersteuning (bijvoorbeeld ten aanzien van somatiek en psychologie). Met instemming van de jongere en/of ouders kan met de betreffende organisaties/instellingen die de nazorg gaan leveren contact gelegd worden en informatie worden overgedragen.

3.5. KLINISCH REDENEREN

Door middel van het multidisciplinair klinisch redeneren wordt bepaald welke diagnostiek er voor de jongere wordt ingezet om te bepalen welke interventie er uiteindelijk passend is (behandeling op maat). Het is geenszins de bedoeling om alle in dit programma beschreven diagnostiek en interventies te gebruiken bij elke patiënt. De verschillende vormen van diagnostiek en interventies zijn onderverdeeld op basis van het ICF-model en helpen, naast het klinisch redeneren bij het kiezen van de juiste instrumenten/interventies.

Voor het klinisch redeneren wordt doorgaans gebruik gemaakt van het KinderRAP (Revalidatie Activiteiten Profiel).[17] Het KinderRAP is opgebouwd uit zes stappen:

1. Beschrijving basisgegevens
2. Hulpvragen van jongere, gezin en hulpverleners
3. Niveau van functioneren op activiteiten- en participatieniveau
4. Het kernprobleem
5. De hoofddoelstelling van de behandeling
6. Gedefinieerde behandeldoelen voor een gedefinieerde periode.

Ook de HOAC II (Hypothesis-Oriented Algorithm for Clinicians II) is een manier van klinisch redeneren die veel gebruikt wordt in de MSR. Hierbij vormen hypotheses de basis van de beschrijving van de mogelijk onderliggende oorzaken van de huidige problemen/de te verwachten problemen.[18] Het opstellen van de hypothesen helpt bij het verder gestructureerd in kaart brengen van de problemen, door middel van klinimetrie, waarna het kiezen van de juiste interventie en de evaluatie daarvan volgt.

In dit model worden er zes stappen onderscheiden die helpen bij het in kaart brengen van het probleem van de patiënt en de hoe dit probleem aan te pakken.

1. Oriënteren op de situatie
2. Beschrijven klinische probleemstelling
3. Het doen van (aanvullend) onderzoek
4. Bepalen van het beleid
5. Beoordelen van het beloop
6. Evaluatie van het proces

Naast de hierboven beschreven methoden zijn er ook andere modellen voor klinisch redeneren beschikbaar. Ieder centrum kan hierin een eigen keuze maken. Het klinisch redeneren is een continu proces tijdens de begeleiding van de jongere, zodat deze optimale zorg ontvangt die bijgestuurd wordt waar en wanneer nodig om zo de patiënt maximaal te ondersteunen in het behalen van zijn/haar doelen.

RAAMWERK KLINIMETRIE

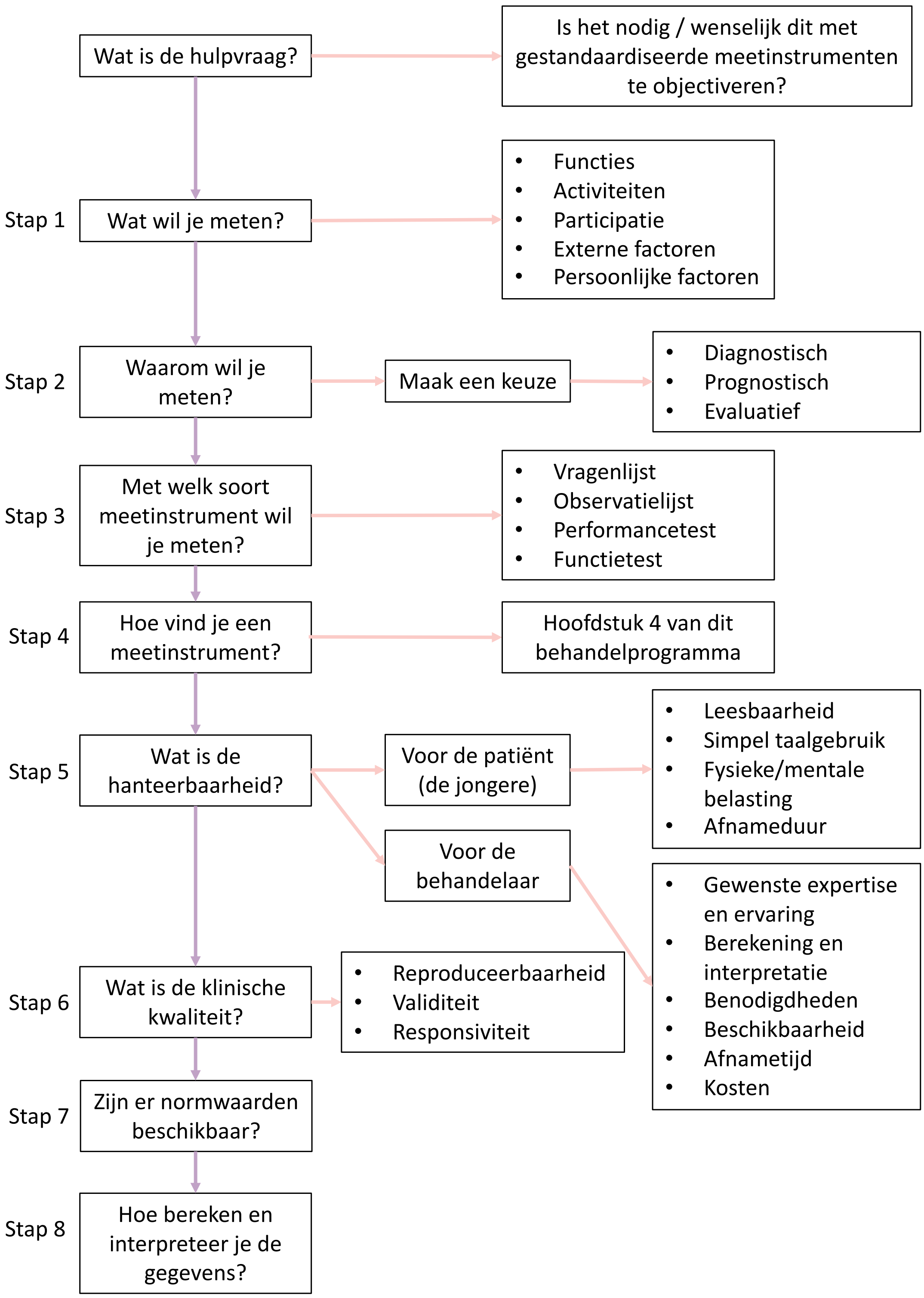

Het raamwerk klinimetrie voor 'evidence based products', voortkomend uit het stappenplan voor het selecteren en gebruiken van meetinstrumenten. Bron:Swinkels et al. 2015

4

4. BEHANDELPROGRAMMA - DIAGNOSTIEK EN INTERVENTIES

In dit hoofdstuk staat het overzicht van de diagnostiek en interventies voor jongeren met NAH en hun ouders beschreven voor de volgende disciplines:

- Psychologie/Orthopedagogiek (4.1)
- (Kinder)fysiotherapie (4.2)
- Ergotherapie (4.3)
- Logopedie (4.4)
- Maatschappelijk werk (4.5)
- Andere disciplines (4.6)

De diagnostiek en interventies worden per discipline beschreven, ingedeeld op basis van het ICF-model. Het interdisciplinaire karakter van de MSR maakt dat de afbakening niet strikt is: disciplines kunnen gebruik maken van elkaars diagnostiek en interventies.
De hieronder beschreven diagnostiek en interventies zijn niet allemaal specifiek ontwikkeld voor jongeren met NAH, maar betreffen de meest gebruikte en als best bruikbaar beoordeelde diagnostiek en interventies op basis van landelijke consensus. Waar mogelijk is er een omschrijving van wetenschappelijke onderbouwing weergegeven.
Op de volgende pagina's wordt per discipline eerst een stroomschema diagnostiek of interventies weergegeven, waarna de specifieke diagnostiek en interventies stuk voor stuk in de opvolgende tabellen beschreven worden.
Een apart overzicht is in 4.7 toegevoegd met de beste en meest gebruikte materialen voor psycho-educatie.

4.1. PSYCHOLOGIE /ORTHOPEDAGOGIEK

DIAGNOSTIEK EN INTERVENTIES

STROOMSCHEMA DIAGNOSTIEK PSYCHOLOGIE

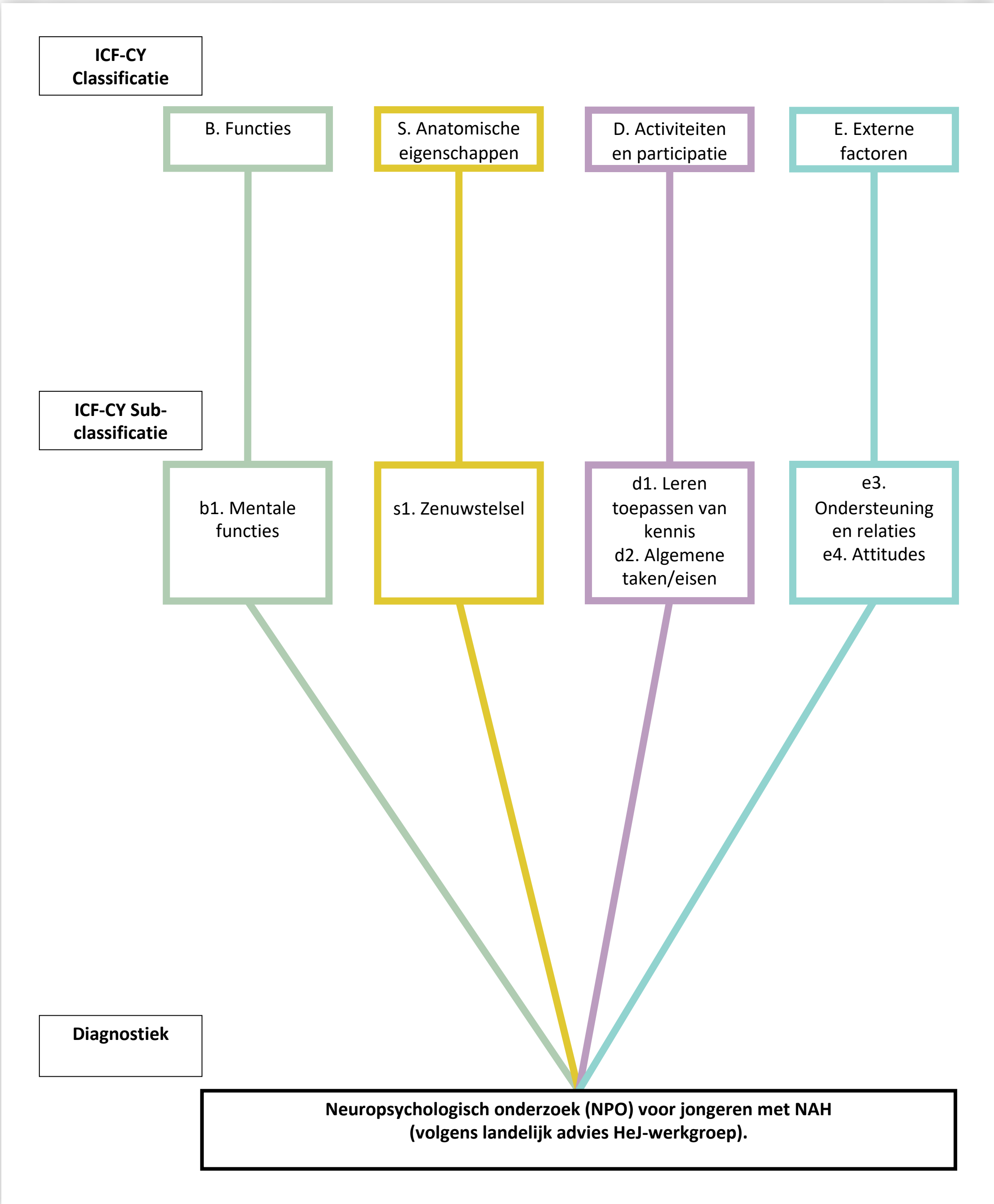

1

DIAGNOSTIEK

NEUROPSYCHOLOGISCH ONDERZOEK (NPO)

ICF-niveau

ICF overstijgend op alle domeinen

**WAT MEET DEZE TEST PRIMAIR**	Volgens landelijk advies voor jongeren met NAH: NPO door GZ-psycholoog (neuro-cognitief functioneren). Het NPO brengt de sterke en zwakke kanten van het cognitief functioneren in kaart en kijkt naar mogelijkheden van de persoon en is gericht op zo goed mogelijke participatie in onderwijs/dagelijks leven. Aan de hand van het onderzoek wordt een sterkte-zwakte profiel gemaakt en volgen er gerichte adviezen.<br>Het NPO omvat in elk geval testen gericht op het meten van intelligentie, tempo van informatieverwerking, aandacht, geheugen, executieve functies, taalvaardigheden en visueel ruimtelijke vaardigheden en sociaal emotioneel functioneren.<br>Met NPO worden de mogelijkheden en de beperkingen in kaart gebracht. De resultaten dienen om tot een gericht advies te komen waarmee de jongere zich zo optimaal mogelijk kan ontwikkelen. Hierbij kan het gaan over het stellen van een indicatie voor specifieke behandeling (zoals cognitieve revalidatie) of het formuleren van adviezen en het beschrijven van de ondersteuningsbehoefte op de verschillende domeinen in de eigen context (zoals thuis, op school, vrije tijd).
**PROTOCOL/ HANDLEIDING**	Te vinden op: Website Hersenalliantie.
**LEEFTIJDSGROEP**	Kinderen 4-12 jaar / adolescenten 13-17 jaar / jongvolwassenen 18-25 jaar.
**TYPE LETSEL**	Traumatisch: licht, matig, ernstig, niet-traumatisch.
**TIMING**	Minimaal 3 maanden na ontstaan van het letsel.
**DOEL VAN INZETTEN**	In kaart brengen sterke en zwakke kanten.<br>Volgen van de ontwikkeling.
**LITERATUUR EN PUBLICATIES**	‘In deze herziene versie is het advies uit de Zorgstandaard THL 2016 beschreven; zijn binnen de werkgroep HeJ revalidatie opnieuw de verschillende testen voor de domeinen van onderzoek besproken op meetpretentie en praktische bruikbaarheid in de klinische praktijk; is de COTAN geraadpleegd en zijn nieuwe ontwikkelingen uit internationaal onderzoek verwerkt.”<br>Algemene info over NPO bij NAH:<br>Klinische kinderneuropsychologie<br>H. Swaab, A. Bouma, J. Hendriksen & C. König (2011), (red.)<br>Amsterdam: Boom ISBN 9789085062691<br>Handboek Traumatisch hersenletsel<br>J. Spikman (2021; p 177-191).
**CONCLUSIES**	Bruikbaar voor de doelgroep jongeren (4-25 jaar) met NAH en is zowel evidence based als practice based.

STROOMSCHEMA INTERVENTIES PSYCHOLOGIE

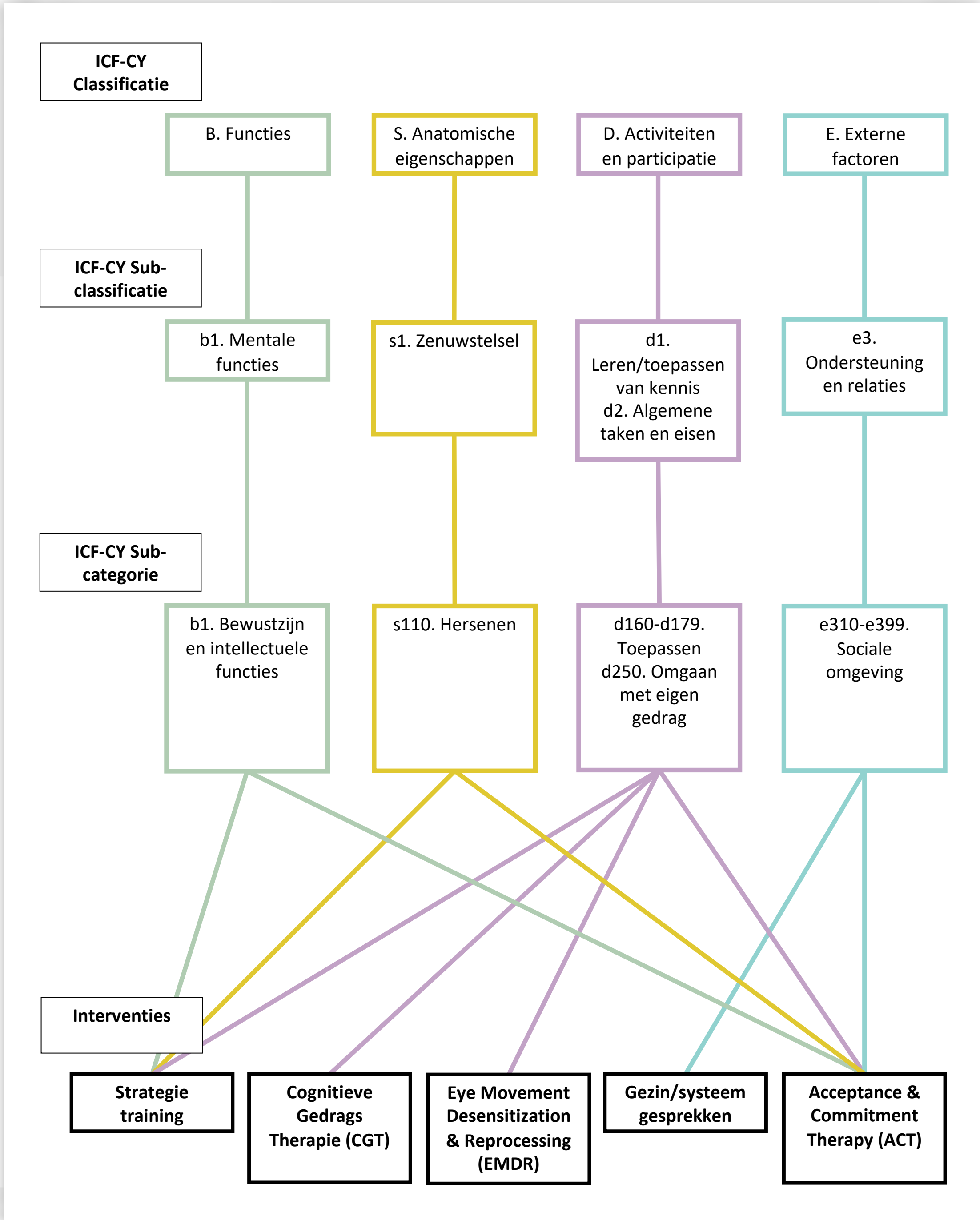

1

INTERVENTIE

STRATEGIETRAINING

ICF-niveau
Functie/anatomie cognitie

**WAAR IS HET VOOR BEDOELD**	Gehele doelgroep met problemen op het gebied van geheugen. Voorbeeld methodes: Meichenbaum (beertjesmethode) / Niet rennen maar plannen 2.0 (zie ook: ergotherapie interventies).
**TIMING**	Voor tijdens MSR.
**LEEFTIJDSGROEP**	Gehele groep van 4-25 jaar.
**TYPE LETSEL**	Alle typen hersenletsel: Traumatisch: licht, matig, ernstig, Niet-traumatisch.
**BESCHRIJVING INTERVENTIE**	Geheugenstrategietraining.
**LITERATUUR EN PUBLICATIES**	Geen onderzoek of internationale publicaties gevonden.
**CONCLUSIES**	Bruikbaar voor de doelgroep jongeren (4-25) met NAH, echter: practice-based.

2

INTERVENTIE

COGNITIEVE GEDRAGSTHERAPIE (CGT)

ICF-niveau
ICF-Overstijgend: Functie/anatomie en activiteiten/participatie

**WAAR IS HET VOOR BEDOELD**	Behandeling van angst, depressie en stress gerelateerd aan NAH.
**TIMING**	Bij start, tijdens en na MSR.
**LEEFTIJDSGROEP**	Kinderen 4-12 jaar / adolescenten 13-17 jaar / jongvolwassenen 18-25 jaar.
**TYPE LETSEL**	Traumatisch: licht, matig, ernstig, niet-traumatisch.
**BESCHRIJVING INTERVENTIE**	Interventies gericht op aanbrengen van veranderingen in het verband tussen wat iemand denkt, voelt en doet.
**PROTOCOL/ HANDLEIDING**	Cursussen beschikbaar via organisaties die cursussen voor psychologen aanbieden, zoals: https://pao.nl/blended-basiscursus-cognitieve-gedragstherapie-volwassenen-en-kinderen-jeugdigen/
**LITERATUUR EN PUBLICATIES**	An evidence-based review of cognitive and behavioral rehabilitation treatment studies in children with acquired brain injury. https://pubmed.ncbi.nlm.nih.gov/17667068/ Evidence-based systematic review of cognitive rehabilitation, emotional, and family treatment studies for children with acquired brain injury literature: From 2006 to 2017. https://pubmed.ncbi.nlm.nih.gov/31671014/
**CONCLUSIES**	Evidentie gevonden, bruikbaar voor de doelgroep jongeren 4-25 met NAH.

3

INTERVENTIE

EYE MOVEMENT DESENSITIZATION AND REPROCESSING (EMDR)

ICF-niveau

ICF-Overstijgend: Functie/anatomie en activiteiten/participatie

**WAAR IS HET VOOR BEDOELD**	Traumaverwerking en acceptatie.
**TIMING**	Tijdens en na MSR.
**LEEFTIJDSGROEP**	Kinderen 4-12 jaar / adolescenten 13-17 jaar / jongvolwassenen 18-25 jaar.
**TYPE LETSEL**	Traumatisch: licht, matig, ernstig, niet-traumatisch.
**BESCHRIJVING INTERVENTIE**	EMDR is een behandelmethode om traumatische ervaringen te verwerken. Deze behandelmethode wordt in Nederland bij volwassenen toegepast sinds 1994 en bij jongeren sinds 2000. Bij EMDR worden traumatische herinneringen eerst geactiveerd en dan gecombineerd met een afleidende taak zoals oogbewegingen. Zo wordt de informatieverwerking in de hersenen gestimuleerd.
**PROTOCOL/ HANDLEIDING**	Te vinden op: https://www.emdr.nl/wat-is-emdr/#hoe-emdr-werkt<br>Literatuur:<br>Ad de Jongh en Erik ten Broeke, Handboek EMDR, Pearson assessment&Information ISBN 109026517769 en ISBN 139789026517761)<br>Renée Beer & Carlijn de Roos (red.) Handboek EMDR bij kinderen en jongeren, 2017, Lannoocampus EAN code 9789401414906<br>Cursus beschikbaar: Er worden cursussen EMDR verzorgd (basiscursus, vervolgcursus en supervisie) door Carlijn de Roos en Renée Beer; EMDR kinderen en jeugd. (www.emdrkindenjeugd.nl)
**LITERATUUR EN PUBLICATIES**	Eye movement desensitization and reprocessing: The state of the art of efficacy in children and adolescent with post traumatic stress disorder.<br>https://pubmed.ncbi.nlm.nih.gov/33421861/
**CONCLUSIES**	Evidentie gevonden, bij kinderen, adolescenten en jongvolwassenen met post-traumatic stress disorder (PTSD).<br>Bruikbaar voor de doelgroep jongeren 4-25 met NAH, echter: practice based.

4

INTERVENTIE

GESPREKKEN MET OUDER, JONGERE EN SOCIALE OMGEVING

Gezinsgesprekken, oudergespreksgroepen, familiebijeenkomsten, school/werkgesprekken.Zie ook interventie maatschappelijk werk

ICF-niveau

Activiteiten/Participatie: tussenmenselijke interacties en relaties

**WAAR IS HET VOOR BEDOELD**	De gezinsgerichte interventies kunnen zich op alle leden van het gezin richten en worden veel ingezet bij de behandeling van jongeren met NAH. Informatie over herstel na hersenletsel, het begrijpen van veranderingen in het gedrag door hersenletsel, erkenning voor het perspectief van alle gezinsleden kan helpen om de ontregeling die hersenletsel in gezinnen met zich meebrengt weer te reguleren. De gezinsgerichte interventies geven ouders input in hoe zij vanuit de gezinscontext hun kind in zijn of haar herstel kunnen stimuleren.
**TIMING**	Bij start en tijdens MSR.
**LEEFTIJDSGROEP**	Kinderen 4-12 jaar / adolescenten 13-17 jaar / jongvolwassenen 18-25 jaar.
**TYPE LETSEL**	Traumatisch: licht, matig, ernstig, Niet-traumatisch.
**BESCHRIJVING INTERVENTIE**	Eigen weg is een praktische en volledige ondersteuningsroute voor het opvoeden van jongeren met hersenletsel. Het is gebaseerd op ‘Hooi op je Vork, het ondersteuningsmodel voor volwassenen met niet-aangeboren hersenletsel.<br>De ondersteuningsroute voor jongeren is ontwikkeld in nauwe samenwerking met ouders en deskundigen op het gebied van niet-aangeboren hersenletsel bij jongeren. De werkwijze omvat vijf stappen, langs vijf stations op de routekaart. Bij elk station sta je met elkaar stil bij bepaalde vragen. Daarna vervolg je de route weer. In 2003 is er door de vrije universiteit van Amsterdam (VU) onderzoek gedaan naar de effectiviteit van dit programma.
**PROTOCOL/ HANDLEIDING**	Te vinden via: Patty van Belle en Judith Zadoks,<br>Eigen Weg www.hooiopjevork.nl<br>Cursus: Scholing voor het gebruiken van dit programma via AXON leertrajecten.

LITERATUUR EN PUBLICATIES	Evidence-based systematic review of cognitive rehabilitation, emotional, and family treatment studies for children with acquired brain injury literature: From 2006 to 2017. https://pubmed.ncbi.nlm.nih.gov/31671014/ Putting the pieces together: Prelimanary efficacy of a family problem solving intervention for children with traumatic brain injury. https://pubmed.ncbi.nlm.nih.gov/16456392/
LITERATUUR EN PUBLICATIES	Direct clinician-delivered versus indirect family-supported rehabilitation of children with traumatic brain injury: a randomized controlled trial. https://pubmed.ncbi.nlm.nih.gov/16175842/ Richtlijn voor organisaties en professionals die betrokken zijn bij de behandeling en begeleiding van gezinnen met een kind met NAH. (Eric Hermans, Judith Zadoks, Rianne Gijzen, Vilans Utrecht 2012).
CONCLUSIES	Evidentie gevonden, bruikbaar voor de doelgroep jongeren 4-25 met NAH.

5

INTERVENTIE

ACCEPTANCE AND COMMITMENT THERAPY (ACT)

Zie ook interventie maatschappelijk werk

ICF-niveau

ICF Functie/anatomie: activiteiten/participatie

**WAAR IS HET VOOR BEDOELD**	Gedragstherapie die cliënten helpt om op een flexibele manier om te gaan met de obstakels die ze tegenkomen (Acceptance), zodat men kan blijven investeren in de dingen die ze écht belangrijk vinden (Commitment). ACT bestaat uit zes verschillende processen/ vaardigheden. Doel van ACT is niet zozeer het reduceren van klachten, maar het ontwikkelen van persoonlijke veerkracht. ACT-vaardigheden in lijn met 'positieve gezondheid'. Aanvullend met EMDR traumaverwerking/acceptatie.
**TIMING**	Tijdens MSR.
**LEEFTIJDSGROEP**	Kinderen 4-12 jaar / adolescenten 13-17 jaar / jongvolwassenen 18-25 jaar.
**TYPE LETSEL**	Traumatisch: licht, matig, ernstig, Niet-traumatisch.
**BESCHRIJVING INTERVENTIE**	Met ACT leert men jongeren gebruik te maken van zes procesgerichte vaardigheden die allemaal sterk met elkaar verbonden zijn.<br>6 stappen van de ACT:<br>- naar gedachten en gevoelens te leren kijken<br>- acceptatie<br>- zelf-als-context<br>- defusie<br>- waarden<br>- toegewijde actie
**PROTOCOL/ HANDLEIDING**	Boek: Acceptance and commitment therapy: The process and practice of mindful change, 2nd ed.<br>Cursus: www.act4kids.nl<br>basiscursus ACT: www.actinactie.nl
**LITERATUUR EN PUBLICATIES**	Acceptance and Commitment Therapy bij kinderen en jongeren. G.M.Samsen& de Heus J.L. (2017) Houten, Bohn Stafleu van Loghum.<br>Hoe ACT kinderen en jongeren kan helpen.<br>Inez Buijck, in Gedragstherapie, jaargang 2021, nr. 3.<br>ACT voor het brein. Hersenproblematiek: verworven of ontwikkelingsvariant.<br>Francis Pascal- Claes, In: Signaal digitaal 2021 nr. 2 (digitale tijdschrift van Sig.vzw).
**CONCLUSIES**	Evidentie gevonden, bij jongvolwassenen.<br>Bruikbaar voor de doelgroep 4-25 met NAH, echter: practice-based.

4.2. (KINDER)FYSIOTHERAPIE

DIAGNOSTIEK EN INTERVENTIES

STROOMSCHEMA DIAGNOSTIEK FYSIOTHERAPIE

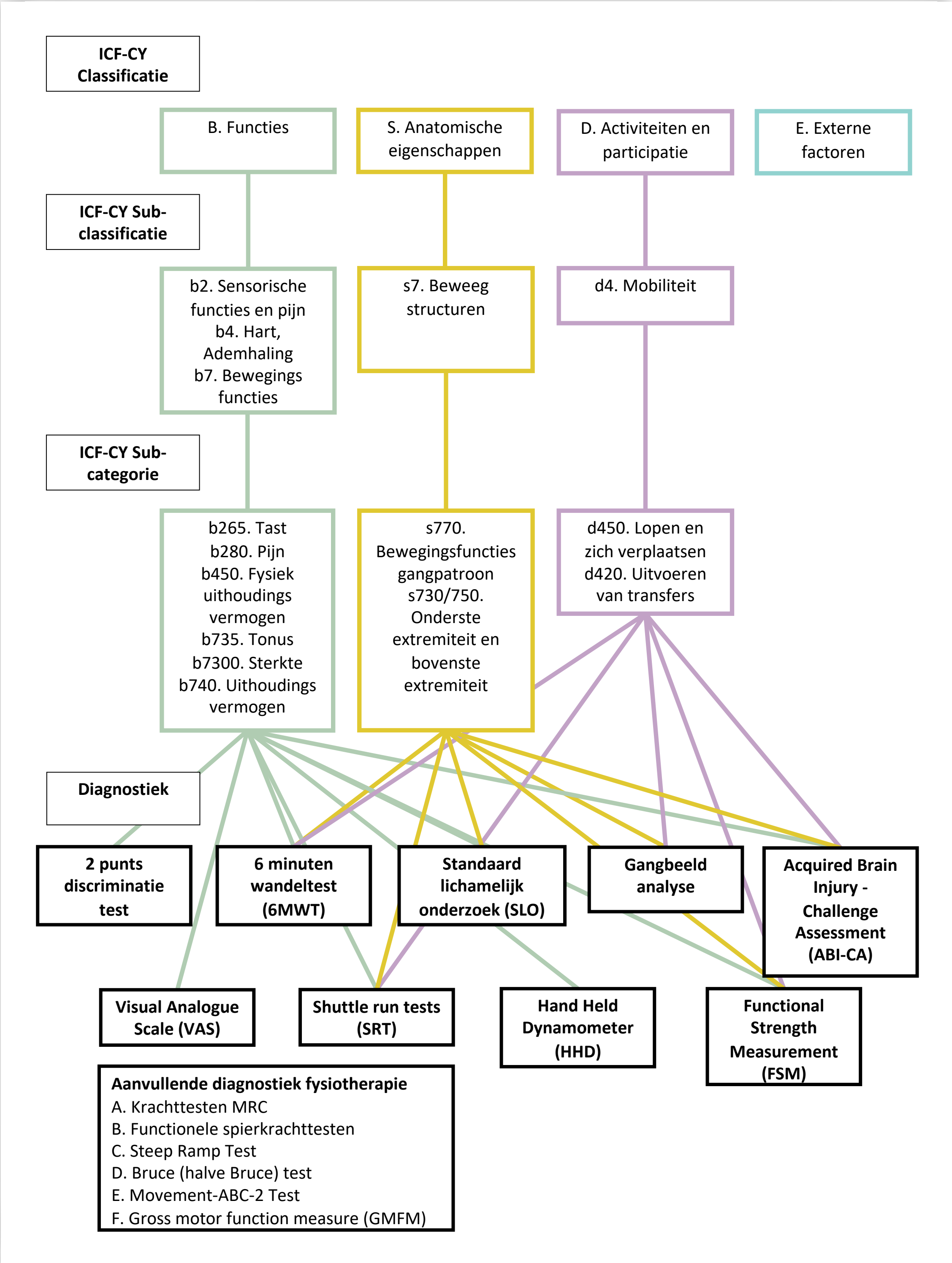

1

DIAGNOSTIEK

2- PUNTS DISCRIMINATIETEST

ICF-niveau

Functie/anatomie: sensorisch en pijn

WAT MEET DEZE TEST PRIMAIR	Sensibiliteit: Toepasbaar als er sprake is of vermoeden van verminderde sensibiliteit.
PROTOCOL/ HANDLEIDING	Test gratis te bekijken op: https://www.physio-pedia.com/Weber_Two-Point_Discrimination_Test<br>Instrumenten te koop op: https://www.sprofit.com/nl/handtherapeut/testing-en-evaluatie/discriminators/touch-test-tweepunts-discriminator<br>https://www.premed.nl/sensibiliteitsmeter-touch-test-2-point-discriminator-voor-de-fijne-en-grove-vingertast-67249/
LEEFTIJDSGROEP	Kinderen 4-12 jaar/ adolescenten 13-17 jaar / jongvolwassenen 18-25 jaar.
TYPE LETSEL	Alle typen hersenletsel.
TIMING	Bij start, tijdens en na MSR.
DOEL VAN INZETTEN	Evaluatief en inventariserend.
LITERATUUR EN PUBLICATIES	Age-dependent Reliability of Semmes-Weinstein and 2-Point Discrimination Tests in Children (2019).<br>https://pubmed.ncbi.nlm.nih.gov/27776051/<br>Two-point discrimination following traumatic brain injury (2005).<br>https://pubmed.ncbi.nlm.nih.gov/15749417/
NORMWAARDEN BESCHIKBAAR	Nee.
CONCLUSIES	Goed bruikbaar bij jongeren vanaf 4 jaar bij vermoeden van verminderde sensibiliteit. Bruikbaar voor de doelgroep jongeren (4-25 jaar) met NAH, echter: practice-based.

2

DIAGNOSTIEK

VISUAL ANALOGUE SCALE (VAS) SCALE VOOR PIJN

ICF-niveau

Functie/anatomie: sensorisch en pijn

WAT MEET DEZE TEST PRIMAIR	Pijn: Toepasbaar als er sprake is of vermoeden van pijn.<br>LET OP: De VAS-schaal wordt voor vele doeleinden gebruikt. Hierbij wordt de uitkomstmaat mede bepaald door de doelgroep, de tijdsspanne en de setting waarin de schaal wordt toegepast.
PROTOCOL/ HANDLEIDING	Te vinden op: https://meetinstrumentenzorg.nl/instrumenten/visual-analogue-scale/<br>Te koop op: https://feetcare4you.nl/product/vas-meter/ en https://www.fysiosupplies.nl/vas-score-liniaal<br>Cursus beschikbaar: nee
LEEFTIJDSGROEP	Kinderen 4-12 jaar/ adolescenten 13-17 jaar / jongvolwassenen 18-25 jaar.
TYPE LETSEL	Alle typen hersenletsel.
TIMING	Bij start, tijdens en na MSR.
DOEL VAN INZETTEN	Evaluatief en inventariserend.
LITERATUUR EN PUBLICATIES	Pain: a review of three commonly used pain rating scales.<br>https://pubmed.ncbi.nlm.nih.gov/16000093/<br>Recommendations for selection of self-report pain intensity measures in children and adolescents: a systematic review and quality assessment of measurement properties.<br>https://pubmed.ncbi.nlm.nih.gov/30180088/<br>The Faces Pain Scale for the self-assessment of the severity of pain experienced by children: development, initial validation and preliminary investigation for ratio scale properties.<br>https://pubmed.ncbi.nlm.nih.gov/2367140/<br>The creation, validation, and continuing development of the Oucher: a measure of pain intensity in children. https://pubmed.ncbi.nlm.nih.gov/1479552/
NORMWAARDEN BESCHIKBAAR	Nee.
NORMWAARDEN	N.v.t.
CONCLUSIES	Wel evidentie gevonden, wordt met name voor acute pijn gebruikt. Matige onderbouwing voor chronische pijn. Niet goed bruikbaar bij kinderen onder 6 jaar. Bruikbaar voor de doelgroep jongeren (4-25 jaar) met NAH met (vermoeden van) pijn.

3

DIAGNOSTIEK

6 MINUTEN WANDELTEST (6MWT)

ICF-niveau

Functie/anatomie: bewegingssysteem

WAT MEET DEZE TEST PRIMAIR	Loopafstand in een vast tijdsbestek. Aerobe inspanningstest voor patiënten die laag-belastbaar zijn.
PROTOCOL/ HANDLEIDING	Te vinden op: https://meetinstrumentenzorg.nl/instrumenten/6-minute-walk-test-zes-minuten-wandeltest/<br>Te koop: gratis op bovengenoemde website.<br>Cursus beschikbaar: niet van toepassing.
LEEFTIJDSGROEP	Kinderen 4-12 jaar / adolescenten 13-17 jaar / jongvolwassenen 18-25 jaar.
TYPE LETSEL	Alle typen hersenletsel.
TIMING	Bij start, tijdens en na MSR.
DOEL VAN INZETTEN	Diagnostisch, evaluatief, inventariserend.
LITERATUUR EN PUBLICATIES	The six-minute walk test in healthy children: reliability and validity.<br>https://pubmed.ncbi.nlm.nih.gov/15929962/<br>Test-re-test reproducibility of activity capacity measures for children with an acquired brain injury.<br>https://pubmed.ncbi.nlm.nih.gov/27314152/<br>Reproducibility and smallest real differences of walking and Energy Expenditure Index in children and adolescents with an acquired brain injury.<br>https://pubmed.ncbi.nlm.nih.gov/31403952/
NORMWAARDEN BESCHIKBAAR	Ja, in gezonde Nederlandse populatie.
NORMWAARDEN	Ja, te vinden in bovenstaande literatuur.
CONCLUSIES	Evidentie gevonden en bruikbaar voor de doelgroep jongeren (4-25 jaar) met NAH.

4

DIAGNOSTIEK

20 METER SHUTTLERUN TEST (SRT)

ICF-niveau

Functie/anatomie: bewegingssysteem

(bv. kracht, fysieke conditie etc.)

**WAT MEET DEZE TEST PRIMAIR**	Vaststellen van het aërobe uithoudingsvermogen. Voor hoog belastbare jongeren. Afhankelijk van, fysieke belastbaarheid keuze maken voor de 10 meter SRT (gevalideerd voor cerebrale parese) of de 20 meter SRT (normwaarden gezonde populatie).
**PROTOCOL/ HANDLEIDING**	Te vinden op: https://www.herstelsnel.nl/herstel2/index.php/testen-en-meten/shuttel-run-test<br>Te koop op: https://shuttle-run-test.nl maar ook gratis te downloaden of te beluisteren op youtube.com<br>Cursus beschikbaar: nee.
**LEEFTIJDSGROEP**	Kinderen 4-12 jaar / adolescenten 13-17 jaar / jongvolwassenen 18-25 jaar.
**TYPE LETSEL**	Licht traumatisch, en Niet-traumatisch.
**TIMING**	Bij start, tijdens en na MSR.
**DOEL VAN INZETTEN**	Diagnostisch, evaluatief, inventariserend.
**LITERATUUR EN PUBLICATIES**	Criterion-related validity of the 20-m shuttle run test for estimating cardiorespiratory fitness: A meta-analysis.<br>https://pubmed.ncbi.nlm.nih.gov/26336340/<br>International normative 20 m shuttle run values from 1 142 026 children and youth representing 50 countries.<br>https://pubmed.ncbi.nlm.nih.gov/27208067/<br>Motor fitness in children and adolescents with traumatic brain injury.<br>https://pubmed.ncbi.nlm.nih.gov/8857887/
**NORMWAARDEN BESCHIKBAAR**	Ja, in Nederlandse gezonde populatie (20m SRT) en cerebrale parese (10 meter SRT) populatie.
**NORMWAARDEN**	Ja, te vinden in bovenstaande literatuur.
**CONCLUSIES**	Evidentie gevonden, bruikbaar bij jongeren (4-25 jaar) met NAH.

5

DIAGNOSTIEK

STANDAARD LICHAMELIJK ONDERZOEK (SLO)

ICF-niveau

Functie/anatomie: bewegingssysteem

(bv. kracht, fysieke conditie etc.)

WAT MEET DEZE TEST PRIMAIR	Mobiliteit, spierlengte, ROM, spasticiteit, bij jongeren met centraal motorische parese. Toepasbaar als er sprake is of vermoeden van verminderde selectiviteit, range of motion (ROM) of van spasticiteit.
PROTOCOL/ HANDLEIDING	Te vinden op: Boek (07-03-2019), ISBN: 9789036823210<br>Te koop op: https://www.bsl.nl/shop/handleiding-standaard-lichamelijk-onderzoek-i-9789036823210<br>Cursus beschikbaar: nee
LEEFTIJDSGROEP	Kinderen 4-12 jaar / adolescenten 13-17 jaar / jongvolwassenen 18-25 jaar.
TYPE LETSEL	Traumatisch: licht, matig, ernstig, Niet-traumatisch.
TIMING	Bij start, tijdens en na MSR.
DOEL VAN INZETTEN	Evaluatief/normatief/discriminatief.
LITERATUUR EN PUBLICATIES	Handleiding Standaard Lichamelijk Onderzoek I: Boek (07-03-2019), ISBN: 9789036823210
NORMWAARDEN BESCHIKBAAR	Ja, in Nederland.
NORMWAARDEN	Ja, kinderen met centraal motorische parese, zie ook het handboek.
CONCLUSIES	Evidentie gevonden, bij kinderen met centraal motorische parese. Bruikbaar voor de doelgroep jongeren (4-25) met NAH echter: practice based.

6

DIAGNOSTIEK

KRACHTTESTEN HANDHELD DYNAMOMETER (HHD)

ICF-niveau

Functie/anatomie: bewegingssysteem

(bv. kracht, fysieke conditie etc.)

**WAT MEET DEZE TEST PRIMAIR**	Meet geïsoleerde spierkracht.<br>Heeft voorkeur boven MRC: is sensitiever en betere evidence beschikbaar, echter niet toepasbaar bij MRC < 3.
**PROTOCOL/ HANDLEIDING**	Te vinden op: https://meetinstrumentenzorg.nl/instrumenten/handknijpkrachtmeter-hand-held-dynamometer/<br>Verschillende producten te koop, onder andere via: https://www.fysiosupplies.nl/microfet-2-wireless?gclid=Cj0KCQiA8ICOBhDmARIsAEGI6o1ZanURo_jLaw5ZcebruD-tKQ_h75JUzSOUtHMuEnfkkWo_MP23nL0aAmbaEALw_wcB<br>Cursus beschikbaar: nee.
**LEEFTIJDSGROEP**	Kinderen 4-12 jaar / adolescenten 13-17 jaar / jongvolwassenen 18-25 jaar.
**TYPE LETSEL**	Traumatisch: licht, matig, ernstig, Niet-traumatisch.
**TIMING**	Bij start, tijdens en na MSR.
**DOEL VAN INZETTEN**	Evaluatief/normatief/discriminatief.
**LITERATUUR EN PUBLICATIES**	Hand-held dynamometry in children with traumatic brain injury: within-session reliability.<br>https://pubmed.ncbi.nlm.nih.gov/18703964/<br>Validity and reproducibility of hand-held dynamometry in children aged 4-11 years.<br>https://pubmed.ncbi.nlm.nih.gov/16548089/
**NORMWAARDEN BESCHIKBAAR**	Ja, in gezonde populatie volwassenen en jongeren uit Nederland.
**NORMWAARDEN**	Ja, gezonde populatie volwassenen en jongeren.
**CONCLUSIES**	Evidentie gevonden, bij gezonde populatie volwassenen en jongeren.<br>Bruikbaar voor de doelgroep jongeren (4-25 jaar) met NAH, echter: practice-based.

7

DIAGNOSTIEK

GANGBEELDANALYSE

ICF-niveau

Functie/anatomie: bewegingssysteem

(bv. kracht, fysieke conditie etc.)

**WAT MEET DEZE TEST PRIMAIR**	In kaart brengen van het looppatroon. Alleen te gebruiken als er vermoeden is van spasticiteit en/of er problemen zijn bij het lopen in het ADL met als gevolg spastische bewegingsstoornissen en cerebellaire problematiek met als gevolg coördinatieve stoornissen.
**PROTOCOL/ HANDLEIDING**	Te vinden op: https://www.pe-online.org/public/opleidingdetail.aspx?pid=67&courseID=68380&button=close<br>Cursus beschikbaar: Ja
**LEEFTIJDSGROEP**	Kinderen 4-12 jaar / adolescenten 13-17 jaar / jongvolwassenen 18-25 jaar.
**TYPE LETSEL**	Traumatisch: licht, matig, Niet-traumatisch.
**TIMING**	Bij start, tijdens en na MSR.
**DOEL VAN INZETTEN**	Evaluatief.
**LITERATUUR EN PUBLICATIES**	Observational gait analysis in traumatic brain injury: accuracy of clinical judgment.<br>https://pubmed.ncbi.nlm.nih.gov/19109020/https://pubmed.ncbi.nlm.nih.gov/19109020/
**NORMWAARDEN BESCHIKBAAR**	Nvt.
**CONCLUSIES**	Evidentie gevonden voor doelgroep jongeren (4-25 jaar) NAH.

8

DIAGNOSTIEK

FUNCTIONAL STRENGTH MEASUREMENT (FSM) KRACHTMETING

ICF-niveau

Functie/anatomie: bewegingssysteem

(bv. kracht, fysieke conditie etc.)

**WAT MEET DEZE TEST PRIMAIR**	Functionele spierkracht: beste alternatief indien beschikbaar; statisch; dynamisch; OE/BE.<br>Alternatief: indien niet aan kunnen/willen schaffen: gebruik functionele krachttesten (methode dr. Verschuuren).
**PROTOCOL/ HANDLEIDING**	Te vinden op: http://functionalstrengthmeasurement-fsm.com/<br>Te koop op: http://functionalstrengthmeasurement-fsm.com/<br>Cursus beschikbaar: ja, http://functionalstrengthmeasurement-fsm.com/
**LEEFTIJDSGROEP**	Kinderen 4-12 jaar. Leeftijdsband tot en met 16 jaar komt binnenkort beschikbaar.
**TYPE LETSEL**	Traumatisch: licht, matig, ernstig, Niet-traumatisch.
**TIMING**	Bij start en na MSR.
**DOEL VAN INZETTEN**	Evaluatief/ normatief. Normawaarde alleen bekend voor ‘gezonde’ kinderen van 4 -10 jaar, er wordt momenteel gewerkt aan de FSM-II voor de leeftijdsgroep 11-16 jaar. Normawaarden voor deze leeftijdsband volgen.
**LITERATUUR EN PUBLICATIES**	Reliability and Structural and Construct Validity of the Functional Strength Measurement in Children Aged 4 to 10.<br>https://pubmed.ncbi.nlm.nih.gov/26586864/
**NORMWAARDEN BESCHIKBAAR**	Ja, in gezonde populatie (Nederland).
**NORMWAARDEN**	Ja: gezonde populatie kinderen van 4-12 jaar.
**CONCLUSIES**	Evidentie gevonden, bij gezonde kinderen 4-12 jaar.<br>Bruikbaar voor de doelgroep jongeren (4-25 jaar) met NAH, echter practice based.

9

DIAGNOSTIEK

ABI-CA (ACQUIRED BRAIN INJURY - CHALLENGE ASSESSMENT)

ICF-niveau

Functie/anatomie: bewegingssysteem

(bv. kracht, fysieke conditie etc.)

**WAT MEET DEZE TEST PRIMAIR**	Testbatterij (gebaseerd op GMFM) voor jongeren met licht traumatisch hersenletsel op het gebied van: snelheid, balans, coördinatie. Vereisten: GMFCS-niveau 1 of 2 (in staat tot los te lopen zonder loophulpmiddel).
**PROTOCOL/ HANDLEIDING**	Te vinden op: https://hollandbloorview.ca/research-education/bloorview-research-institute/outcome-measures/abi-challenge-assessment<br>Te koop op: Wanneer cursus behaald is, worden test formulieren gedeeld.<br>Cursus beschikbaar: ja, deze wordt aangeboden in de Hoogstraat, deze cursus is te volgen op verzoek en beschikbaarheid.
**LEEFTIJDSGROEP**	Kinderen 7-12 jaar / adolescenten 13-17 jaar.
**TYPE LETSEL**	Traumatisch: licht/matig.
**TIMING**	Bij start, tijdens en na MSR.
**DOEL VAN INZETTEN**	Evaluatief.
**LITERATUUR EN PUBLICATIES**	Development of a challenge assessment tool for high-functioning children with an acquired brain injury.<br>https://pubmed.ncbi.nlm.nih.gov/20699774/<br>Further development of the response scales of the Acquired Brain Injury Challenge Assessment (ABI-CA).<br>https://pubmed.ncbi.nlm.nih.gov/24020440/<br>Reliability and validity of the acquired brain injury challenge assessment (ABI-CA) in children.<br>https://pubmed.ncbi.nlm.nih.gov/25188447/
**NORMWAARDEN BESCHIKBAAR**	Geen normwaarden beschikbaar.
**NORMWAARDEN**	Ja: voor kinderen en jongeren met NAH.
**CONCLUSIES**	Evidentie gevonden, bij Canadese jongeren met NAH<br>Bruikbaar voor de doelgroep (4-25 jaar) met NAH echter, practice based.

A

AANVULLENDE DIAGNOSTIEK

KRACHTTEST VOLGENS MRC SCHAAL

ICF-niveau

Functie/anatomie: bewegingssysteem

(bv. kracht, fysieke conditie etc.)

WAT MEET DEZE TEST PRIMAIR	Maximaal kracht voor geïsoleerde spiergroepen<br>LET OP: gebruik bij voorkeur en indien beschikbaar een HHD.
PROTOCOL/ HANDLEIDING	Gratis beschikbaar op diverse sites (waaronder kinderneurologie.eu): https://www.kinderneurologie.eu/onderwijsplein/spieronderzoek.php
LEEFTIJDSGROEP	Kinderen 7-12 jaar / adolescenten 13-17 jaar.
TYPE LETSEL	Traumatisch: licht/matig en niet-traumatisch.
TIMING	Bij start, tijdens en na MSR.
DOEL VAN INZETTEN	Evaluatief.
LITERATUUR EN PUBLICATIES	Physical examination--measurement of muscle strength.<br>https://pubmed.ncbi.nlm.nih.gov/11198960/
NORMWAARDEN BESCHIKBAAR	Nvt.
NORMWAARDEN	Nvt.
CONCLUSIES	Betrouwbaar wanneer men de geïsoleerde spiergroep selectief kan aanspannen echter is MRC 4/5 niet goed te onderscheiden. Gebruik HHD bij MRC4 en hoger. Bruikbaar voor de doelgroep jongeren (4-25 jaar) met NAH echter, practice based.

B

AANVULLENDE DIAGNOSTIEK

FUNCTIONELE SPIERKRACHT METINGEN (METHODE DR. VERSCHUUREN)

ICF-NIVEAU

Functie/anatomie: bewegingssysteem

(kracht)

WAT MEET DEZE TEST PRIMAIR	Meet spieruithoudingsvermogen. Wel specifiek voor neurologische aandoeningen. Evidence bij CP, ook toepasbaar voor NAH (indien vergelijkbaar beeld met CP: GMFCS-niveau 1/2: in staat tot los lopen zonder loophulpmiddel).<br>Let op: Gebruik indien beschikbaar de FSM.
PROTOCOL/ HANDLEIDING	Gratis beschikbaar: https://www.kcrutrecht.nl/producten/functionele-krachttest-voor-cp/<br>http://www.netchild.nl/ Olaf Verschuuren.
LEEFTIJDSGROEP	Kinderen 4-12 jaar / adolescenten 13-17 jaar.
TYPE LETSEL	Traumatisch: licht, en niet-traumatisch.
TIMING	Bij start, tijdens en na MSR.
DOEL VAN INZETTEN	Evaluatief.
LITERATUUR EN PUBLICATIES	Reliability of hand-held dynamometry and functional strength tests for the lower extremity in children with Cerebral Palsy<br>https://pubmed.ncbi.nlm.nih.gov/18850351/
NORMWAARDEN BESCHIKBAAR	Nvt.
NORMWAARDEN	Nvt.
CONCLUSIES	Betrouwbare test voor spierkracht/uithoudingsvermogen van de onderste extremiteit bij kinderen met cerebrale parese.<br>Bruikbaar voor de doelgroep jongeren (4-25 jaar) met NAH, echter, practice based.

C

AANVULLENDE DIAGNOSTIEK

STEEP RAMP TEST

ICF-niveau

ICF Functie/anatomie: bewegingssysteem

(bv. kracht, fysieke conditie etc.)

**WAT MEET DEZE TEST PRIMAIR**	Aeroob inspanningsvermogen: maximaaltest (op de fiets). Geschikt voor hoog belastbare jongeren.<br>Indien voorhanden: shuttle run. Als de ruimte dit niet toe laat en indien er een loopband beschikbaar is.
**PROTOCOL/ HANDLEIDING**	Uitleg en protocol te vinden op:<br>https://meetinstrumentenzorg.nl/instrumenten/steep-ramp-test/
**LEEFTIJDSGROEP**	Kinderen 4-12 jaar / adolescenten 13-17 jaar.
**TYPE LETSEL**	Traumatisch: licht, en niet-traumatisch.
**TIMING**	Bij start, tijdens en na MSR.
**DOEL VAN INZETTEN**	Evaluatief.
**LITERATUUR EN PUBLICATIES**	The steep ramp test in healthy children and adolescents: reliability and validity. https://pubmed.ncbi.nlm.nih.gov/22903141/<br>Application of the steep ramp test for aerobic fitness testing in children with cancer.<br>https://pubmed.ncbi.nlm.nih.gov/25426542/
**NORMWAARDEN BESCHIKBAAR**	Ja, zie bovenstaande literatuur.
**NORMWAARDEN**	Gezonde kinderen.
**CONCLUSIES**	Betrouwbaar instrument voor gezonde kinderen en adolescenten en kinderen met oncologische problematiek. Practice based bij jongeren (4-25 jaar) met NAH.

D

AANVULLENDE DIAGNOSTIEK

BRUCE TEST OF HALVE BRUCE TEST

ICF-niveau

ICF Functie/anatomie: bewegingssysteem

(bv. kracht, fysieke conditie etc.)

**WAT MEET DEZE TEST PRIMAIR**	Aerobe inspanningstest: maximaaltest. Indien een jongere lager belastbaar is kan de keuze gemaakt worden om een halve Bruce test uit te voeren.<br>Indien voorhanden: shuttle run. Als de ruimte dit niet toe laat en indien er een loopband beschikbaar is.
**PROTOCOL/ HANDLEIDING**	Beschikbaar op:<br>https://meetinstrumentenzorg.nl/instrumenten/bruce-treadmill-test-protocol/<br>Informatie Bruce.<br>https://meetinstrumentenzorg.nl/wp-content/uploads/instrumenten/Bruce-test-form.pdf
**LEEFTIJDSGROEP**	Kinderen 4-12 jaar / adolescenten 13-17 jaar.
**TYPE LETSEL**	Traumatisch: licht, en niet-traumatisch.
**TIMING**	Bij start, tijdens en na MSR.
**DOEL VAN INZETTEN**	Evaluatief.
**LITERATUUR EN PUBLICATIES**	Maximal oxygen intake and nomographic assessment of functional aerobic impairment in cardiovascular disease.<br>https://pubmed.ncbi.nlm.nih.gov/4632004/
**NORMWAARDEN BESCHIKBAAR**	Ja.
**NORMWAARDEN**	Gezonde kinderen.
**CONCLUSIES**	Bruikbaar voor jongeren (4-25 jaar) met NAH echter, practice based.

E

AANVULLENDE DIAGNOSTIEK

MOVEMENT ABC-2 TEST

ICF-niveau

ICF Functie/anatomie: bewegingssysteem

(bv. kracht, fysieke conditie etc.)

**WAT MEET DEZE TEST PRIMAIR**	Grove en fijne motoriek test. Fijne motoriek-onderdeel: uit te voeren door ergotherapie.<br>Wordt alleen aanbevolen bij jongeren met niet-neurologische aandoeningen. Beste alternatief: ABI-CA.
**PROTOCOL/ HANDLEIDING**	Te koop op: https://www.pearsonclinical.nl/movement-abc-2-nl-movement-assessment-battery-children
**LEEFTIJDSGROEP**	Kinderen 4-12 jaar / adolescenten 13-17 jaar.
**TYPE LETSEL**	Traumatisch: licht, en niet-traumatisch.
**TIMING**	Bij start, tijdens en na MSR.
**DOEL VAN INZETTEN**	Evaluatief.
**LITERATUUR EN PUBLICATIES**	Structural validity of the Movement ABC-2 test: factor structure comparisons across three age groups.<br>https://pubmed.ncbi.nlm.nih.gov/21330102/
**NORMWAARDEN BESCHIKBAAR**	Ja.
**NORMWAARDEN**	Gezonde kinderen.
**CONCLUSIES**	Test is valide, betrouwbaar en responsief in populatie zonder neurologische schade en in bijv DCD. Niet geschikt voor jongeren met neurologische schade. Niet geschikt voor NAH doelgroep.<br>Kan gebruikt worden om jongeren met zichzelf te vergelijken.<br>Normwaarden niet (altijd) geschikt.

F

AANVULLENDE DIAGNOSTIEK

GROSS MOTOR FUNCTION MEASURE (GMFM)

ICF-niveau

ICF Functie/anatomie: bewegingssysteem

(bv. kracht, fysieke conditie etc.)

WAT MEET DEZE TEST PRIMAIR	Motorisch functioneren.<br>ABI-CA heeft de voorkeur: specifieker voor de doelgroep en betere evidentie voor de doelgroep (ABI-CA is op basis van de GMFM).
PROTOCOL/ HANDLEIDING	Uitleg en protocol te vinden op: https://meetinstrumentenzorg.nl/instrumenten/gross-motor-function-measure/
LEEFTIJDSGROEP	Kinderen 4-12 jaar / adolescenten 13-17 jaar.
TYPE LETSEL	Traumatisch: ernstig, en niet-traumatisch.
TIMING	Bij start, tijdens en na MSR.
DOEL VAN INZETTEN	Evaluatief.
LITERATUUR EN PUBLICATIES	Reliability and responsiveness of the gross motor function measure-88 in children with cerebral palsy.<br>https://pubmed.ncbi.nlm.nih.gov/23139425/
NORMWAARDEN BESCHIKBAAR	Ja, zie bovenstaande literatuur.
NORMWAARDEN	Kinderen met cerebrale parese en syndroom van Down.
CONCLUSIES	Bruikbaar voor doelgroep jongeren (4-25 jaar) met NAH echter, practice based. Evidence based bij kinderen met CP en Down, (nog) niet specifiek voor jongeren 4-25 met NAH.

STROOMSCHEMA INTERVENTIES FYSIOTHERAPIE

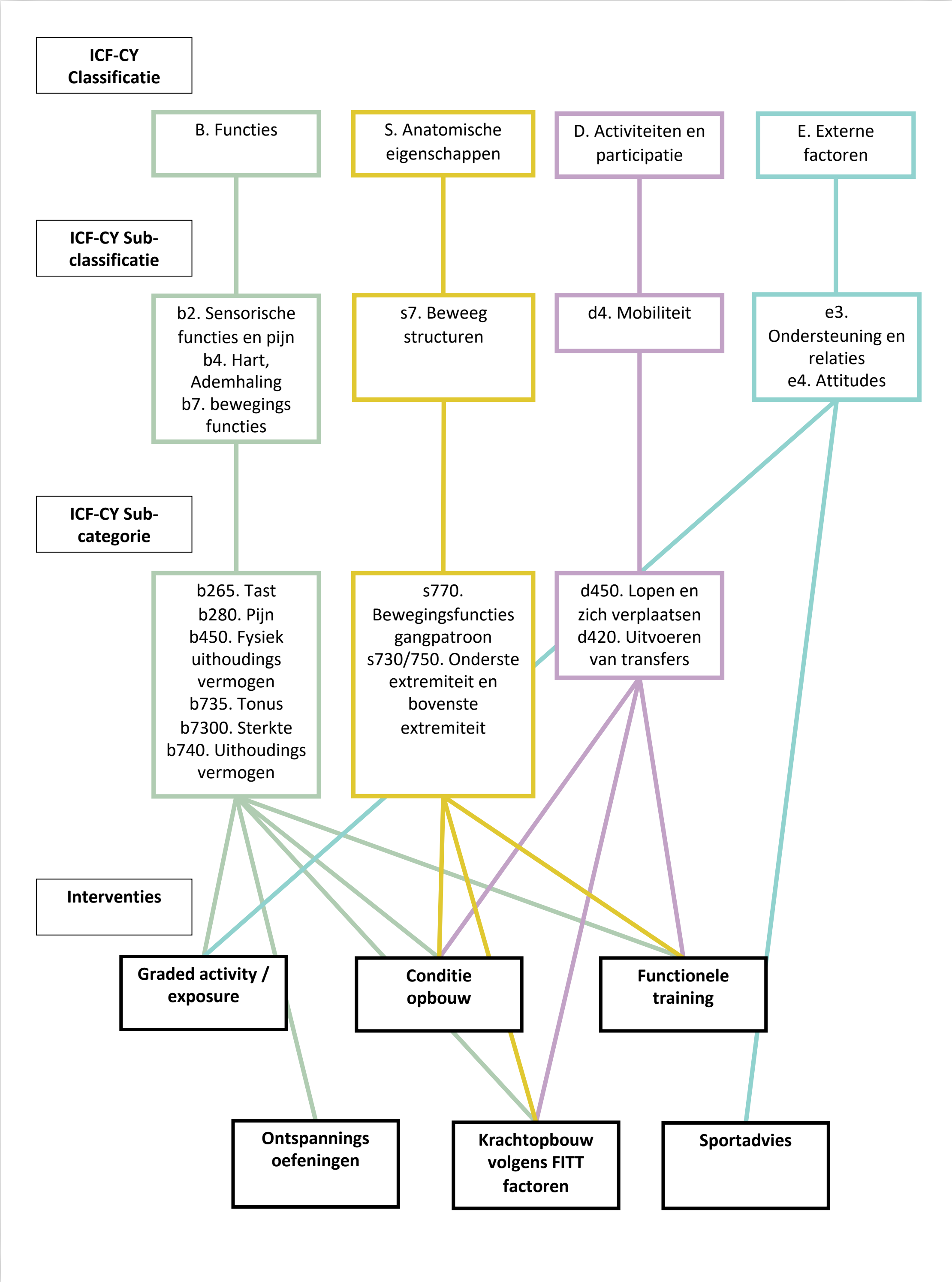

1

INTERVENTIE

GRADED ACTIVITY / GRADED EXPOSURE (ZIE OOK: BIJ DISCIPLINE ERGOTHERAPIE)

ICF-niveau

Functie/anatomie: mentaal en zenuwstelsel

(b.v. cognitie, stemming, slaap)

**WAAR IS HET VOOR BEDOELD**	Effectief voor adolescenten en volwassenen met rugpijn, chronische pijn en vermoeidheid.<br>Graded activity is een behandelmethode waarbij je leert fysieke activiteiten stapsgewijs op te bouwen. Door de geleidelijke opbouw ervaar je dat meer bewegen niet automatisch meer pijn betekent.<br>Graded exposure is helpend wanneer activiteiten vermeden worden uit angst om te bewegen. Deze aanpak laat je ervaren dat bewegen niet altijd leidt tot negatieve gevolgen (bijvoorbeeld weefselschade of extreme pijntoename).
**TIMING**	Tijdens en na MSR.
**LEEFTIJDSGROEP**	Adolescenten 13-17 jaar / jongvolwassenen 18-25 jaar.
**TYPE LETSEL**	Traumatisch: licht, matig.
**BESCHRIJVING INTERVENTIE**	Graded Activity is een integratieve, gestructureerde behandelvorm, gebaseerd op cognitieve en gedragsmatige leertheorieën gericht op het gradueel opbouwen van activiteiten volgens een tijdcontingent schema, waarbij de jongere leert zelfstandig zijn activiteitenniveau op te bouwen en te handhaven.<br>Graded Exposure is het geleidelijk aan blootstellen aan activiteiten die je vermijdt uit angst. Dit kan angst zijn voor toename van de pijn of angst voor het beschadigen van je lichaam. Graded exposure kan helpen activiteiten weer op te bouwen en angst te verminderen.
**PROTOCOL/ HANDLEIDING**	Te vinden op: Boek: Understanding and Treating Fear of Pain, geredigeerd door G.J.G. Asmundson, J.W. S. Vlaeyen, G. Crombez. Physiother Can. 2008 Spring; 60(2): 196–197<br>Cursus beschikbaar: verschillende, met name gericht op chronische pijnklachten.
**LITERATUUR EN PUBLICATIES**	The clinical application of pain neuroscience, graded motor imagery, and graded activity with complex regional pain syndrome—A case report.<br>https://pubmed.ncbi.nlm.nih.gov/30499359/
**CONCLUSIES**	Evidentie gevonden, nl bij adolescenten en volwassenen met rugpijn, chronische pijn en vermoeidheid.<br>Bruikbaar voor de doelgroep jongeren (4-25 jaar) met NAH echter, practice based.

2

INTERVENTIE

ONTSPANNINGSOEFENINGEN

ICF-niveau

Functies/anatomie: sensorisch

**WAAR IS HET VOOR BEDOELD**	Ontspanning teweegbrengen in het lichaam (ondersteunende interventie).
**TIMING**	Tijdens en na MSR.
**LEEFTIJDSGROEP**	Kinderen 4-12 jaar / adolescenten 13-17 jaar / jongvolwassenen 18-25 jaar.
**TYPE LETSEL**	Traumatisch: licht, matig, ernstig, Niet-traumatisch.
**BESCHRIJVING INTERVENTIE**	Het geven van ontspanningsoefeningen tijdens therapie of als huiswerkoefeningen. Ondersteunend aan interventies.
**PROTOCOL/ HANDLEIDING**	Te vinden op:<br>Verschillende apps, zoals ‘kindermeditaties’ en de app van VGZ.<br>Ontspanningsoefening – spieren.<br>Kindermeditatie – het strand.
**LITERATUUR EN PUBLICATIES**	Nvt.
**CONCLUSIES**	Geen evidentie (onderbouwing) gevonden.<br>Wel bruikbaar voor de doelgroep jongeren (4-25 jaar) met NAH: practice based.

3

INTERVENTIE

CONDITIE-OPBOUW

ICF-niveau

Functies/anatomie: bewegingssysteem

(bv. kracht, fysieke fitheid)

**WAAR IS HET VOOR BEDOELD**	Opbouwen van conditie inden deze door welke oorzaak dan ook verminderd is. Conditieopbouw is een belangrijke voorspeller voor goed functioneren in ADL, ook onder jongeren met NAH.
**TIMING**	Tijdens en na MSR.
**LEEFTIJDSGROEP**	Kinderen 4-12 jaar / adolescenten 13-17 jaar / jongvolwassenen 18-25 jaar.
**TYPE LETSEL**	Traumatisch: licht, matig, ernstig.
**BESCHRIJVING INTERVENTIE**	Opbouwen van conditie.
**PROTOCOL/ HANDLEIDING**	Te vinden op: Inspanningsfysiologie.
**LITERATUUR EN PUBLICATIES**	Boek: inspanningsfysiologie<br>Fitness training for cardiorespiratory conditioning after traumatic brain injury.<br>https://pubmed.ncbi.nlm.nih.gov/29286534/<br>Endurance training and cardiorespiratory conditioning after traumatic brain injury.<br>https://pubmed.ncbi.nlm.nih.gov/20473091/
**CONCLUSIES**	Evidentie gevonden, bij jongeren met traumatisch hersenletsel. Bruikbaar voor de doelgroep jongeren (4-25 jaar) met NAH.

4

INTERVENTIE

KRACHT OPBOUW VOLGENS FITT FACTOREN

ICF-niveau

Functies/anatomie: bewegingssysteem

(bv. kracht, fysieke fitheid)

**WAAR IS HET VOOR BEDOELD**	Opbouwen van spierkracht als deze om wat voor reden dan ook verminderd is na letsel.
**TIMING**	Bij start, tijdens en na MSR.
**LEEFTIJDSGROEP**	Kinderen 4-12 jaar / adolescenten 13-17 jaar / jongvolwassenen 18-25 jaar.
**TYPE LETSEL**	Traumatisch: licht, matig, ernstig, Niet-traumatisch.
**BESCHRIJVING INTERVENTIE**	Opbouwen van spierkracht.
**PROTOCOL/ HANDLEIDING**	Te vinden op: inspanningsfysiologie/kinderfysiotherapie (boek).
**LITERATUUR EN PUBLICATIES**	FITT criteria ACSM.
**CONCLUSIES**	Bruikbaar voor de doelgroep jongeren (4-25 jaar) met NAH echter, practice based.

5

INTERVENTIE

FUNCTIONELE TRAINING

ICF-niveau

ICF Functies/anatomie: bewegingssysteem

(bv. kracht, fysieke fitheid)

**WAAR IS HET VOOR BEDOELD**	Toepassen van functionele training indien dit geïndiceerd is.
**TIMING**	Bij start, tijdens en na MSR.
**LEEFTIJDSGROEP**	Kinderen 4-12 jaar / adolescenten 13-17 jaar / jongvolwassenen 18-25 jaar.
**TYPE LETSEL**	Traumatisch: licht, matig, ernstig, Niet-traumatisch.
**BESCHRIJVING INTERVENTIE**	Functionele training maakt gebruik van bewegingspatronen met meerdere gewrichten. Het gaat om oefeningen die totale lichaamsbewegingen en -patronen nabootsen die je ziet in sport en dagelijkse activiteiten in het algemeen.
**PROTOCOL/ HANDLEIDING**	Functionele training kan op verschillende manieren worden aangeboden. Deze behoeven niet altijd protocol of handleiding. Functionele training in de vorm van: Mega Power training, fietstraining, oefenen in de context, Balans training: met allerlei dynamische/statische evenwichtstaken, hulpvraaggericht te werk gaan. Ook de principes van motorisch leren bij CNA (centraal neurologische aandoeningen) horen hierbij.<br>Strategietraining en stappenplannen worden hier vaak additioneel voor ingezet (zie interventies psychologie en ergotherapie).
**LITERATUUR EN PUBLICATIES**	Rehabilitation after traumatic brain injury.<br>https://pubmed.ncbi.nlm.nih.gov/25702231/<br>Physiotherapy after traumatic brain injury: a systematic review of the literature.<br>https://pubmed.ncbi.nlm.nih.gov/18415716/
**CONCLUSIES**	Evidentie gevonden, bij de doelgroep jongeren (4-25 jaar) met NAH.

6

INTERVENTIE

SPORTADVIES

ICF-niveau

Activiteiten/participatie: maatschappelijk leven

(bv. vereniging, sportclub)

**WAAR IS HET VOOR BEDOELD**	Jongeren die vragen hebben ontremd een sport, dit kan zijn vragen over de huidige sport of het zoeken van een nieuwe geschikte sport. Indien deze hulpvraag er is: begeleiden naar sportactiviteiten op participatieniveau.<br>Indien aanwezig: sportloket inzetten.
**TIMING**	Na MSR.
**LEEFTIJDSGROEP**	Kinderen 4-12 jaar / adolescenten 13-17 jaar / jongvolwassenen 18-25 jaar.
**TYPE LETSEL**	Traumatisch: licht, matig, ernstig, Niet-traumatisch.
**BESCHRIJVING INTERVENTIE**	Adviesgesprek op het gebied van sport.
**PROTOCOL/ HANDLEIDING**	Hiervoor is geen protocol beschikbaar.
**LITERATUUR EN PUBLICATIES**	Nvt.
**CONCLUSIES**	Geen evidentie (onderbouwing) gevonden voor jongeren (4-25 jaar) met NAH, practice based.

4.3. ERGOTHERAPIE

DIAGNOSTIEK EN INTERVENTIES

STROOMSCHEMA DIAGNOSTIEK ERGOTHERAPIE

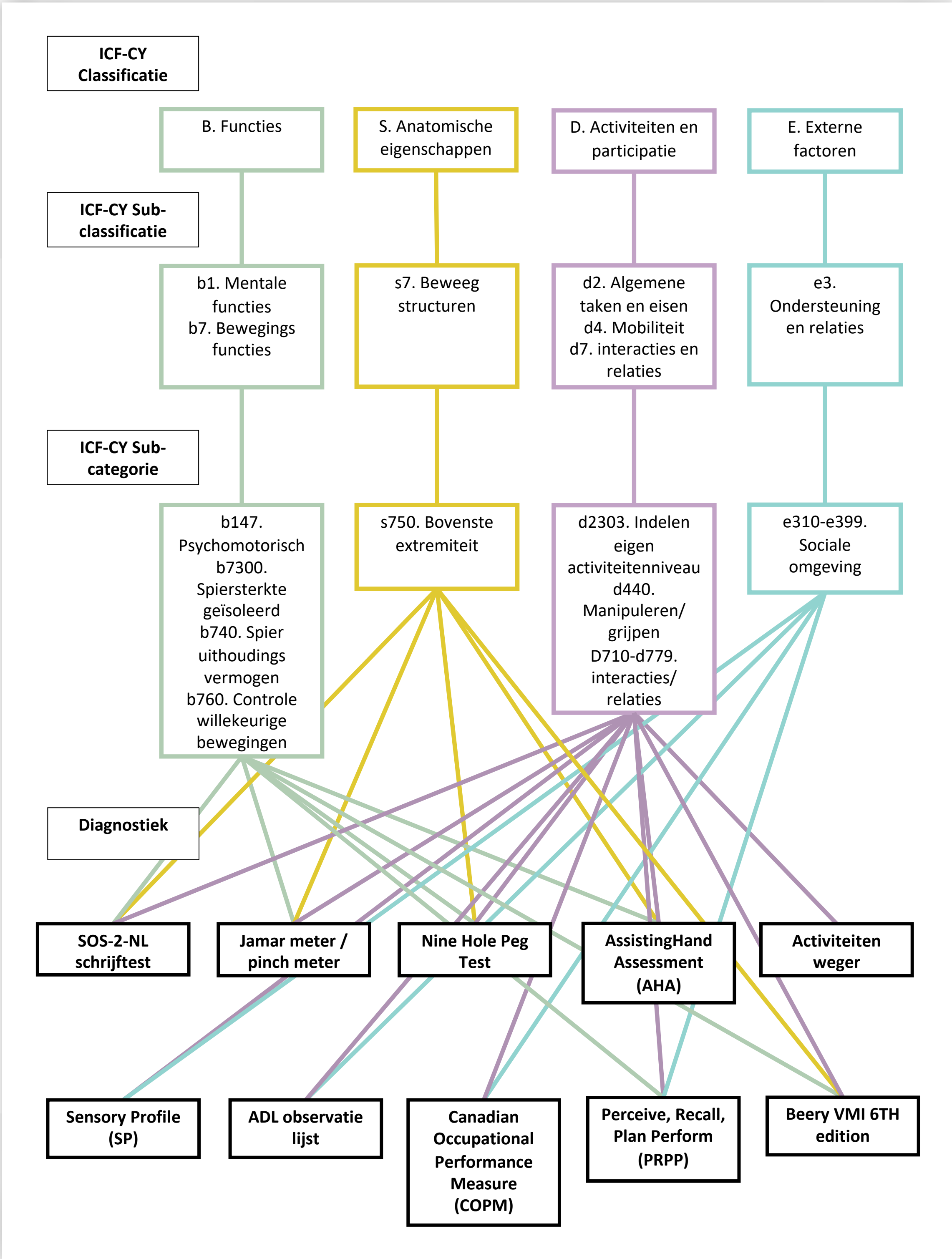

1

DIAGNOSTIEK

SYSTEMATISCHE OPSPORING SCHRIJFPROBLEMEN-2 NL (SOS-2-NL) SCHRIJFTEST

ICF-nivea

Activiteiten/participatie: mobiliteit

**WAT MEET DEZE TEST PRIMAIR**	Schrijfobservatie. Vooral jongere kinderen. In te zetten bij vermoeden van problemen bij schrijven.
**PROTOCOL/ HANDLEIDING**	Te vinden op: https://meetinstrumentenzorg.nl/instrumenten/systematische-opsporing-schrijfproblemen-gereviseerde-versie/<br>Te koop op: https://www.pearsonclinical.nl/sos-2-workshop<br>Cursus beschikbaar: Ja.
**LEEFTIJDSGROEP**	Kinderen 4-12 jaar / adolescenten 13-17 jaar.
**TYPE LETSEL**	Traumatisch: licht, matig, Niet-traumatisch.
**TIMING**	Bij start en na MSR.
**DOEL VAN INZETTEN**	Evaluatief/normatief/discriminatief/inventariserend.
**LITERATUUR EN PUBLICATIES**	Treatment outcome in children with developmental coordination disorder; responsiveness of six outcome measures.<br>https://pubmed.ncbi.nlm.nih.gov/32615810/<br>SOS: a screening instrument to identify children with handwriting impairments.<br>https://pubmed.ncbi.nlm.nih.gov/22515913/
**NORMWAARDEN BESCHIKBAAR**	Ja, in Nederland.
**NORMWAARDEN**	Ja: kinderen < 18 met DCD, zie bovenstaande literatuur.
**CONCLUSIES**	Evidentie gevonden, bij kinderen < 18 met DCD.<br>Bruikbaar voor de doelgroep jongeren 4-18 met NAH echter, practice based.

2

DIAGNOSTIEK

JAMAR METER / PINCH METER

ICF-niveau

Functie/anatomie: bewegingssysteem

(bijvoorbeeld kracht en fysieke conditie)

**WAT MEET DEZE TEST PRIMAIR**	Brengt (maximale) knijpkracht in kaart. Additioneel met HHD indien knijpkracht in kaart gebracht wordt.
**PROTOCOL/ HANDLEIDING**	Te vinden op: https://meetinstrumentenzorg.nl/instrumenten/handknijpkrachtmeter-hand-held-dynamometer/ Te koop op: https://www.fysiosupplies.nl/microfet-2-wireless?gclid=Cj0KCQiA8ICOBhDmARIsAEGI6o1ZanURo_jLaw5
**LEEFTIJDSGROEP**	Kinderen 4-12 jaar / adolescenten 13-17 jaar / jongvolwassenen 18-25 jaar.
**TYPE LETSEL**	Traumatisch: licht, matig, ernstig, Niet-traumatisch.
**TIMING**	Bij start, tijdens en na MSR.
**DOEL VAN INZETTEN**	Evaluatief.
**LITERATUUR EN PUBLICATIES**	Grip and pinch strength: normative data for adults. https://pubmed.ncbi.nlm.nih.gov/3970660/
**NORMWAARDEN BESCHIKBAAR**	Nee.
**NORMWAARDEN**	Nee.
**CONCLUSIES**	Evidentie gevonden, bij volwassenen. Bij kinderen wisselend en vaak discutabel. Bruikbaar voor de doelgroep jongeren (4-25 jaar) met NAH echter, practice based.

3

DIAGNOSTIEK

NINE HOLE PEG TEST

ICF-niveau

Activiteiten/participatie: mobiliteit

WAT MEET DEZE TEST PRIMAIR	Coördinatie/fijne motoriek (kan het fijne motoriek deel van de Movement abc vervangen).
PROTOCOL/HANDLEIDING	Te vinden op: https://meetinstrumentenzorg.nl/instrumenten/nine-hole-peg-test/<br>Te koop op: https://www.thuiszorgwebshop.nl/peg-test-9-hole.html<br>https://www.sprofit.com/nl/handtherapeut/testing-en-evaluatie/pennenborden/jamar-9-hole-peg-test<br>https://www.stockxmedical.com/en/peg-test-9-hole.html<br>https://www.youtube.com/watch?v=kkyfI5OvfJo<br>Cursus beschikbaar: nee.
LEEFTIJDSGROEP	Volwassenen 18-25 waarbij de handfunctie is aangedaan.
TYPE LETSEL	Alle typen letsel waarbij de handfunctie is aangedaan.
TIMING	Kan op elk moment in diagnostiek of tijdens de interventie worden afgenomen.
DOEL VAN INZETTEN	Inventariserend / Evaluatief.
LITERATUUR EN PUBLICATIES	Adult norms for a commercially available Nine Hole Peg Test for finger dexterity.<br>https://pubmed.ncbi.nlm.nih.gov/14527120/<br>Normative and Validation Studies of the Nine-hole Peg Test with children.<br>https://pubmed.ncbi.nlm.nih.gov/10883762/
NORMWAARDEN BESCHIKBAAR	Ja.
NORMWAARDEN	Beschikbaar Amerikaans onderzoek: zie bovenstaande literatuur.
CONCLUSIES	Evidentie gevonden, bij gezonde kinderen en volwassenen met CVA<br>Bruikbaar voor de doelgroep jongeren (18-25 jaar) met NAH echter, practice based.

4

DIAGNOSTIEK

ASSISTING HAND ASSESSMENT (AHA)

ICF-niveau

Activiteiten/participatie: mobiliteit

WAT MEET DEZE TEST PRIMAIR	In kaart brengen gebruik en functie van de aangedane arm/hand. Alleen toe te passen bij patiënten met (vermoeden van) hemiparetisch beeld in de BE.
PROTOCOL/ HANDLEIDING	Te vinden op: https://www.ahanetwork.se/#:~:tekst=The%20<br>Te koop op: https://handfast-webshop.myshopify.com/products/aha-test-kit<br>Cursus beschikbaar: Ja.
LEEFTIJDSGROEP	Kinderen 4-12 jaar / adolescenten 13-17 jaar / jongvolwassenen 18-25 jaar.
TYPE LETSEL	Traumatisch: licht, matig, ernstig, Niet-traumatisch.
TIMING	Tijdens MSR.
DOEL VAN INZETTEN	Evaluatief/discriminatief.
LITERATUUR EN PUBLICATIES	The Assisting Hand Assessment: current evidence of validity, reliability, and responsiveness to change.<br>https://pubmed.ncbi.nlm.nih.gov/17376135/<br>Psychometric properties of a revised version of the Assisting Hand Assessment (Kids-AHA 5.0).<br>https://pubmed.ncbi.nlm.nih.gov/26507383/<br>Reliability of the Assisting Hand Assessment in adolescents.<br>https://pubmed.ncbi.nlm.nih.gov/28555755/<br>Development of the Assisting Hand Assessment for adolescents (Ad-AHA) and validation of the AHA from 18 months to 18 years.<br>https://pubmed.ncbi.nlm.nih.gov/27291981/
NORMWAARDEN BESCHIKBAAR	Ja, Zweden, Nederland.
NORMWAARDEN	Ja, gezonde kinderen (Nederland).
CONCLUSIES	Evidentie gevonden, bij kinderen en jongvolwassenen, (nog) niet NAH specifiek, maar onderzoek naar AHA-ABI loopt.<br>Bruikbaar voor de doelgroep jongeren (4-25 jaar) met NAH met een hemiparetisch beeld echter, practice based.

5

DIAGNOSTIEK

ACTIVITEITENWEGER

ICF-niveau

ICF-Overstijgend: Functie/anatomie en activiteiten/participatie

WAT MEET DEZE TEST PRIMAIR	De Activiteitenweger is een hulpmiddel voor het plannen van dagelijkse activiteiten. Het is bedoeld als richtlijn. Hoeveel activiteiten kan de patiënt uitvoeren waarbij de patiënt binnen zijn grenzen blijft. Met deze duidelijke richtlijn wordt een balans bereikt waar de patiënt zich prettig bij voelt, en meer ontspanning ervaart. Daarnaast wordt onderzocht of het mogelijk is om weer energie weer op te bouwen.
PROTOCOL/ HANDLEIDING	Te downloaden via https://www.verdermethersenletsel.nl/a/activiteitenweger.<br>https://activity-matters.com/
LEEFTIJDSGROEP	Kinderen 4-12 jaar / adolescenten 13-17 jaar / jongvolwassenen 18-25 jaar.
TYPE LETSEL	Traumatisch: licht, matig, ernstig, Niet-traumatisch.
TIMING	Bij start en tijdens MSR.
DOEL VAN INZETTEN	Discriminatief.
LITERATUUR EN PUBLICATIES	Geen publicaties gevonden, echter wel:<br>Wetenschappelijk onderzoek – Activity-Matters
NORMWAARDEN BESCHIKBAAR	Nee.
CONCLUSIES	Geen evidentie gevonden, maar wel bruikbaar bij jongeren (4-25 jaar) met NAH echter, practice based.

6

DIAGNOSTIEK

SENSORY PROFILE (SP) NL

ICF-niveau

ICF-Overstijgend: Functie/anatomie en activiteiten/participatie

WAT MEET DEZE TEST PRIMAIR	Sensorische informatieverwerking: meet via een vragenlijst hoe goed jongeren sensorische informatie kunnen verwerken in alledaagse situaties.
PROTOCOL/ HANDLEIDING	Te vinden op: https://www.pearsonassessments.com/content/dam/school/global/clinical/us/assets/sensoyprofile2/sensory-profile-2-technical-summary.pdf<br>Te koop op: https://www.pearsonclinical.nl/sensory-profile-nl
LEEFTIJDSGROEP	Kinderen 4-12 jaar, adolescenten tot 18 jaar<br>Oudervragenlijst 4 – 12<br>Zelfbeoordelingslijst 11 - 65
TYPE LETSEL	Traumatisch: licht, matig, ernstig, Niet-traumatisch.
TIMING	Na MSR.
DOEL VAN INZETTEN	Discriminatief.
LITERATUUR EN PUBLICATIES	The Sensory Profile: A discriminant analysis of children with and without disabilities.<br>https://pubmed.ncbi.nlm.nih.gov/9544354/
NORMWAARDEN BESCHIKBAAR	Nee.
NORMWAARDEN	Nvt.
CONCLUSIES	Evidentie gevonden bij gezonde jongeren en bij jongeren met ASS / ADD / ADHD. Bruikbaar voor de doelgroep jongeren (4-25 jaar) met NAH echter, practice based.

7

DIAGNOSTIEK

ALGEMEEN DAGELIJKS LEVEN (ADL) OBSERVATIELIJST

ICF-niveau

Activiteiten/participatie: zelfverzorging

WAT MEET DEZE TEST PRIMAIR	ADL observatie. Alternatief: COPM/PRPP, ook samen te gebruiken.
PROTOCOL/ HANDLEIDING	Te vinden op: https://info.ergotherapie.nl/file/download/default/1B0F3FBD40404B9EC28E85BA5E5D42A1/ADL%20Zelfverzorgingsobservatie.pdf
LEEFTIJDSGROEP	Kinderen vanaf 8 jaar / adolescenten 13-17 jaar / jongvolwassenen 18-25 jaar.
TYPE LETSEL	Traumatisch: licht, matig, ernstig, Niet-traumatisch.
TIMING	Bij start en tijdens MSR.
DOEL VAN INZETTEN	Evaluatief.
LITERATUUR EN PUBLICATIES	Zie literatuurlijst in de handleiding.
NORMWAARDEN BESCHIKBAAR	Nee.
NORMWAARDEN	Nee.
CONCLUSIES	Geen evidentie (onderbouwing) gevonden.<br>Bruikbaar voor de doelgroep jongeren (4-25) met NAH echter, practice based.

8

DIAGNOSTIEK

CANADIAN OCCUPATIONAL PERFORMANCE MEASURE (COPM)

ICF-niveau

ICF-Overstijgend: Functie/anatomie en activiteiten/participatie

**WAT MEET DEZE TEST PRIMAIR**	De COPM wordt ingezet om (ergotherapeutische) behandeldoelen te stellen. Deze doelen zijn gebaseerd op de door de cliënt ervaren problemen in het dagelijks handelen. De COPM identificeert de belangrijkste problemen die de cliënt ervaart en meet de veranderingen in het beeld dat de cliënt heeft van zijn handelen gedurende het behandelproces. De COPM richt zich op drie gebieden: zelfredzaamheid, productiviteit en ontspanning.
**PROTOCOL/ HANDLEIDING**	Te vinden op: https://meetinstrumentenzorg.nl/instrumenten/canadian-occupational-performance-measure/<br>https://meetinstrumentenzorg.nl/wp-content/uploads/instrumenten/COPM-form-1.pdf<br>Aanvulling: Aanvullende handleiding voor afname van de COPM bij kinderen zelf - Amsterdam UMC locatie AMC - definitief 11-3-2019.pdf (ergotherapie.nl)<br>Te koop op: https://www.thecopm.ca/buy/translations/<br>Cursus beschikbaar: Ja.
**LEEFTIJDSGROEP**	Kinderen 4-12 jaar / adolescenten 13-17 jaar / jongvolwassenen 18-25 jaar.
**TYPE LETSEL**	Traumatisch: licht, matig, ernstig, Niet-traumatisch.
**TIMING**	Bij start en na MSR.
**DOEL VAN INZETTEN**	Evaluatief/effectiviteit/inventariserend/diagnostisch.
**LITERATUUR EN PUBLICATIES**	The Canadian Occupational Performance Measure: a research and clinical literature review. Canadian journal of occupational therapy.<br>https://pubmed.ncbi.nlm.nih.gov/15586853/<br>Measurement Properties of the Canadian Occupational Performance Measure: A Systematic Review.<br>https://pubmed.ncbi.nlm.nih.gov/34817593/
**NORMWAARDEN BESCHIKBAAR**	Nee.
**NORMWAARDEN**	Nvt.
**CONCLUSIES**	Evidentie gevonden bij jongeren maar niet betrouwbaar.<br>Bruikbaar voor de doelgroep jongeren (4-25 jaar) met NAH echter, practice based.

9

DIAGNOSTIEK

PERCEIVE, RECALL, PLAN AND PERFORM (PRPP)

ICF-niveau

ICF Activiteiten/participatie: Leren en toepassen van kennis

**WAT MEET DEZE TEST PRIMAIR**	Taakanalyse. De uitkomsten van een PRPP zegt iets over hoe de processen van informatieverwerking invloed hebben op het uitvoeren van betekenisvolle, dagelijkse activiteiten. De procesanalyse doet uitspraak over sterke en zwakke kanten van het functioneren van een jongere met NAH en biedt ingang voor therapie.
**PROTOCOL/ HANDLEIDING**	Cursus beschikbaar: https://www.prpp.nl/
**LEEFTIJDSGROEP**	Kinderen 4-18 jaar.
**TYPE LETSEL**	Traumatisch: licht, matig, ernstig, Niet-traumatisch.
**TIMING**	Bij start, tijdens en na MSR.
**DOEL VAN INZETTEN**	Het is een 'criterion-referenced' instrument; 'de lat' wordt individueel en/of per observatie bepaald. Bij een PRPP-observatie gaat het bepalen van de criteria waar de cliënt aan moet voldoen bij het uitvoeren van een bepaalde taak, in overleg met cliënt(systeem) en de ergotherapeut.
**LITERATUUR EN PUBLICATIES**	Cognitive strategy use in adults with acquired brain injury.<br>https://doi.org/10.1080/02699052.2020.1725837<br>Effectiveness of Information Processing Strategy Training on Academic Task Performance in Children with Learning Disabilities: A Pilot Study.<br>https://doi.org/10.1155/2017/6237689<br>Impaired awareness of deficits and cognitive strategy use in occupational performance of persons with acquired brain injury (ABI).<br>https://www.emerald.com/insight/content/doi/10.1108/IJOT-10-2019-0012/full/pdf
**NORMWAARDEN BESCHIKBAAR**	Nee.
**CONCLUSIES**	Geen evidentie gevonden. Bruikbaar voor de doelgroep jongeren (4-25 jaar) met NAH echter, practice based.

10

DIAGNOSTIEK

THE BEERY-BUKTENICA DEVELOPMENTAL TEST OF VISUAL – MOTOR INTEGRATION

6TH Edition (Beery VMI 6TH edition)

ICF-niveau

ICF Functie/Anatomie: mentaal en zenuwstelsel

(b.v. cognitie, stemming, slaap).

WAT MEET DEZE TEST PRIMAIR	Meet de oog-hand-coördinatie gerelateerd aan de leeftijd, waarbij ook onderscheidt gemaakt kan worden tussen visuele perceptie en motorische coördinatie.
PROTOCOL/ HANDLEIDING	Te koop op: https://www.pearsonclinical.nl/beery-vmi
LEEFTIJDSGROEP	Hele leeftijdsrange.
TYPE LETSEL	Traumatisch: licht, matig, ernstig, Niet-traumatisch.
TIMING	Bij start en na MSR.
DOEL VAN INZETTEN	Evaluatief.
LITERATUUR EN PUBLICATIES	Nvt.
NORMWAARDEN BESCHIKBAAR	Nvt.
NORMWAARDEN	Nvt.
CONCLUSIES	Betrouwbaar instrument voor kinderen met ontwikkelingsachterstand. Bruikbaar voor doelgroep jongeren (4-25 jaar) met NAH echter, practice based.

STROOMSCHEMA INTERVENTIES ERGOTHERAPIE

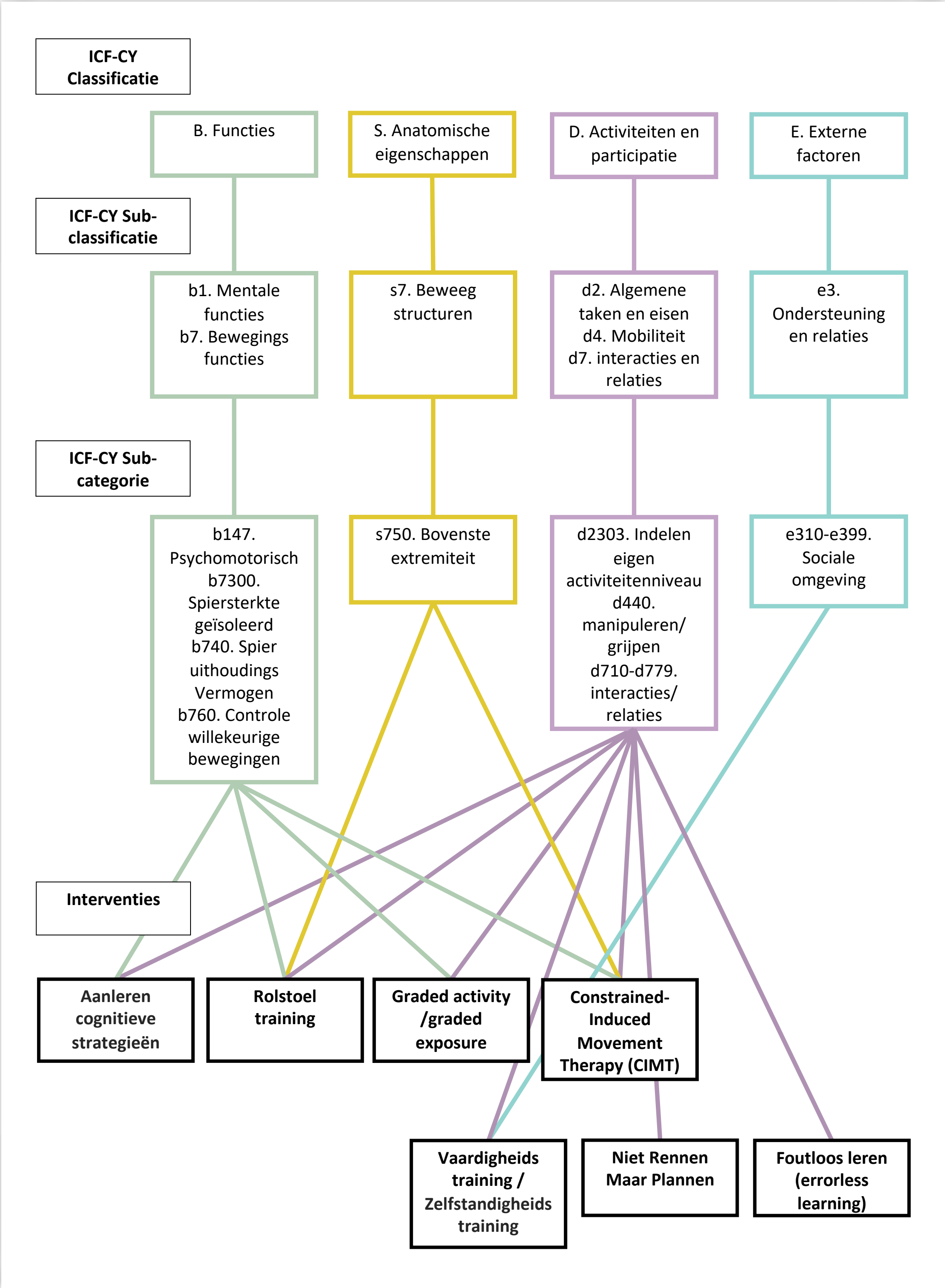

1

INTERVENTIE

AANLEREN VAN COGNITIEVE STRATEGIEËN CO-OP / ZELFINSTRUCTIEMETHODE (MEICHENBAUM)

ICF-niveau

ICF Functie/anatomie: mentaal en zenuwstelsel

(b.v. cognitie, stemming, slaap)

**WAAR IS HET VOOR BEDOELD**	Het doel van de Meichenbaum methode is de jongere te leren zijn handelingen bewust te sturen door een externe structuur te bieden. De methode ordent het denkproces en voorkomt impulsief, ongestructureerd en “trial-and-error”-gedrag. Aanleren van cognitieve strategieën.
**TIMING**	Tijdens MSR.
**LEEFTIJDSGROEP**	Kinderen 4-12 jaar / adolescenten 13-17 jaar.
**TYPE LETSEL**	Traumatisch: licht, matig, ernstig, Niet-traumatisch.
**BESCHRIJVING INTERVENTIE**	Tijdens het aanleren van deze cognitieve strategie leert de jongeren een taakaanpak in vijf fasen; probleemanalyse, oplossingsstrategie, uitvoering, reflectie, evaluatie.
**PROTOCOL/ HANDLEIDING**	Te vinden in (werk)boek: Timmerman, K. (2003). *Kinderen met aandachts- en werkhoudingsproblemen.*<br>Cursus beschikbaar: https://www.stibco.nl/product/zelfinstructiemethode-van-meichenbaum/
**LITERATUUR EN PUBLICATIES**	Evidence-based systematic review of cognitive rehabilitation, emotional, and family treatment studies for children with acquired brain injury literature: From 2006 to 2017.<br>https://www.tandfonline.com/doi/abs/10.1080/09602011.2019.1678490<br>Searching for effective components of cognitive rehabilitation for children and adolescents with acquired brain injury: a systematic review.<br>https://www.tandfonline.com/doi/full/10.1080/02699052.2018.1458335
**CONCLUSIES**	Evidentie gevonden, bij kinderen met DCD.<br>Bruikbaar voor de doelgroep jongeren 4-25 met NAH echter, practice based.

2

INTERVENTIE

ROLSTOELTRAINING

ICF-niveau

Functies/anatomie en activiteiten/participatie: mobiliteit

**WAAR IS HET VOOR BEDOELD**	Aanleren van rolstoelvaardigheden.
**TIMING**	Bij start, tijdens en na MSR.
**LEEFTIJDSGROEP**	Kinderen 4-12 jaar / adolescenten 13-17 jaar / jongvolwassenen 18-25 jaar.
**TYPE LETSEL**	Traumatisch: licht, matig, ernstig, Niet-traumatisch.
**BESCHRIJVING INTERVENTIE**	Aanleren van rolstoelvaardigheden.
**PROTOCOL/ HANDLEIDING**	Te vinden via onderstaande literatuur (protocol).
**LITERATUUR EN PUBLICATIES**	The effects of wheelchair mobility skills and exercise training on physical activity, fitness, skills and confidence in youth using a manual wheelchair. https://pubmed.ncbi.nlm.nih.gov/33874820/
**CONCLUSIES**	Evidentie gevonden, bij jongeren die rolstoelgebonden zijn. Bruikbaar voor de doelgroep jongeren (4-25 jaar) met NAH die rolstoel gebonden zijn.

3

INTERVENTIE

GRADED ACTIVITY / GRADED EXPOSURE. ZIE OOK DISCIPLINE FYSIOTHERAPIE

ICF-niveau

Functie/anatomie: mentaal en zenuwstelsel

(b.v. cognitie, stemming, slaap)

**WAAR IS HET VOOR BEDOELD**	Effectief voor adolescenten en volwassenen met rugpijn, chronische pijn en vermoeidheid.<br>Graded activity is een behandelmethode waarbij je leert fysieke activiteiten stapsgewijs op te bouwen. Door de geleidelijke opbouw ervaar je dat meer bewegen niet automatisch meer pijn betekent.<br>Graded exposure is helpend wanneer activiteiten vermeden worden uit angst om te bewegen. Deze aanpak laat je ervaren dat bewegen niet altijd leidt tot negatieve gevolgen (bijvoorbeeld weefselschade of extreme pijntoename).
**TIMING:**	Tijdens en na MSR.
**LEEFTIJDSGROEP**	Adolescenten 13-17 jaar / jongvolwassenen 18-25 jaar.
**TYPE LETSEL**	Traumatisch: licht, matig.
**BESCHRIJVING INTERVENTIE**	Graded Activity is een integratieve, gestructureerde behandelvorm, gebaseerd op cognitieve en gedragsmatige leertheorieën gericht op het gradueel opbouwen van activiteiten volgens een tijdcontingent schema, waarbij de cliënt leert zelfstandig zijn activiteitenniveau op te bouwen en te handhaven.<br>Graded Exposure is het geleidelijk aan blootstellen aan activiteiten die je vermijdt uit angst. Dit kan angst zijn voor toename van de pijn of angst voor het beschadigen van je lichaam. Graded exposure kan helpen activiteiten weer op te bouwen en angst te verminderen.
**PROTOCOL/ HANDLEIDING**	Te vinden op: Boek: Understanding and Treating Fear of Pain, geredigeerd door G.J.G. Asmundson, J.W. S. Vlaeyen, G. Crombez. Physiother Can. 2008 Spring; 60(2): 196–197<br>Cursus beschikbaar: verschillende, met name gericht op chronische pijnklachten.
**LITERATUUR EN PUBLICATIES**	The clinical application of pain neuroscience, graded motor imagery, and graded activity with complex regional pain syndrome—A case report.<br>https://pubmed.ncbi.nlm.nih.gov/30499359/
**CONCLUSIES**	Evidentie gevonden, bij adolescenten en volwassenen met rugpijn, chronische pijn en vermoeidheid.<br>Bruikbaar voor de doelgroep jongeren (4-25 jaare) met NAH echter, practice based.

4

INTERVENTIE

CONSTRAINT-INDUCED MOVEMENT THERAPY (CIMT)

ICF-niveau

Functies/anatomie: bewegingssysteem

(bv. kracht, fysieke fitheid)

**WAAR IS HET VOOR BEDOELD**	Verbeteren arm-handfunctie en de bi-manuele samenwerking.
**TIMING**	Bij start, tijdens en na MSR.
**LEEFTIJDSGROEP**	Kinderen 4-12 jaar / adolescenten 13-17 jaar / jongvolwassenen 18-25 jaar.
**TYPE LETSEL**	Traumatisch: licht, matig, ernstig, Niet-traumatisch.
**BESCHRIJVING INTERVENTIE**	Verbeteren arm-handfunctie middels gestructureerde interventie.
**PROTOCOL/ HANDLEIDING**	Te vinden op: https://www.physio-pedia.com/Constraint-Induced_Movement_Therapy_(CIMT)
**LITERATUUR EN PUBLICATIES**	Constraint-induced movement therapy after stroke. https://pubmed.ncbi.nlm.nih.gov/25772900/
**CONCLUSIES**	Evidentie gevonden, bij volwassenen met CVA en kinderen met cerebrale parese (CP).<br>Bruikbaar voor de doelgroep jongeren (4-25 jaar) met NAH echter, practice based.

5

INTERVENTIE

VAARDIGHEIDSTRAINING / ZELFSTANDIGHEIDSTRAINING

ICF-niveau

ICF Activiteiten/participatie: leren en toepassen van kennis en zelfverzorging

WAAR IS HET VOOR BEDOELD	Het aanleren of verbeteren van vaardigheden, met name agendagebruik, gebruik dag- en weekplanning, oefenen schoolse vaardigheden. De interventie is op basis van: behandelen / begeleiden / coachen/ adviseren (voorzieningen, aanpassen in en aanpassen van de omgeving (sensorisch, belasting, educatie).
TIMING	Tijdens MSR.
LEEFTIJDSGROEP	Kinderen 4-12 jaar / adolescenten 13-17 jaar / jongvolwassenen 18-25 jaar.
TYPE LETSEL	Traumatisch: licht, matig, ernstig, Niet-traumatisch.
BESCHRIJVING INTERVENTIE	aanleren of verbeteren van vaardigheden.
PROTOCOL/ HANDLEIDING	Nvt.
LITERATUUR EN PUBLICATIES	Geen evidentie (onderbouwing) gevonden.
CONCLUSIES	Bruikbaar voor de doelgroep jongeren (4-25 jaar) met NAH echter, practice based.

6

INTERVENTIE

NIET RENNEN MAAR PLANNEN 2.0

ICF-niveau

ICF Activiteiten/participatie: leren en toepassen van kennis en zelfverzorging

**WAAR IS HET VOOR BEDOELD**	Niet Rennen Maar Plannen is ontwikkeld voor jongeren met verschillende vormen van hersenletsel zoals CVA, trauma, MS, of tumoren. Het programma is geschikt voor jongeren met weinig tot geen motorische uitval, maar wel lichte cognitieve stoornissen ervaren. Het programma is gemaakt voor behandelaars. De behandelaar kijkt, samen met de jongere, bij welke onderdelen de jongere het meest baat heeft.
**TIMING**	Tijdens MSR.
**LEEFTIJDSGROEP**	Kinderen 4-12 jaar / adolescenten 13-17 jaar / jongvolwassenen 18-25 jaar.
**TYPE LETSEL**	Traumatisch: licht, matig, ernstig, Niet-traumatisch.
**BESCHRIJVING INTERVENTIE**	Vanuit Richtlijn “NeuroPsychologische revalidatie 2017” “Niveau 1; Het is aangetoond dat compensatietraining, d.w.z. het aanleren van het gebruik van zowel externe als interne strategieën, effectief is bij patiënten met lichte geheugenstoornissen na hersenletsel. Het is aangetoond dat compensatietraining, d.w.z. het aanleren van het gebruik van externe strategieën, effectief is bij patiënten met matige tot ernstige geheugenstoornissen na hersenletsel. Het is aangetoond dat compensatietraining het meest effectief is wanneer hersenletsel patiënten enigszins onafhankelijk functioneren in het dagelijks leven, zelf inzicht hebben in hun geheugenproblemen en in staat en gemotiveerd zijn om continu actief en zelfstandig geheugenstrategieën te gebruiken.”
**PROTOCOL/ HANDLEIDING**	Nvt.
**LITERATUUR EN PUBLICATIES**	Niet Rennen maar Plannen. (kcrutrecht.nl)
**CONCLUSIES**	Bruikbaar voor de doelgroep jongeren 4-25 met NAH echter, practice based.

7

INTERVENTIE

FOUTLOOS LEREN (ERRORLESS LEARNING)

ICF-niveau

ICF Activiteiten/participatie: leren en toepassen van kennis

WAAR IS HET VOOR BEDOELD	Motorisch leren vs doelgericht leren (met stappen): doelgericht leren werkt beter. Foutloos leren vs trial & error, bij mensen met geheugenproblemen: geleerde taak verbeterde bij foutloos leren meer. Foutloos leren gecombineerd met doelgericht leren blijkt tot betere taakuitvoering.
TIMING	Tijdens MSR.
LEEFTIJDSGROEP	Kinderen 4-12 jaar / adolescenten 13-17 jaar / jongvolwassenen 18-25 jaar.
TYPE LETSEL	Traumatisch: licht, matig, ernstig, Niet-traumatisch
BESCHRIJVING INTERVENTIE	Gestructureerd motorisch leren.
PROTOCOL/ HANDLEIDING	Nvt.
LITERATUUR EN PUBLICATIES	Errorless learning improves memory performance in children with acquired brain injury: A controlled comparison of standard and self-generation Techniques. http://dx.doi.org/10.1080/09602011.2012.686820
CONCLUSIES	Bruikbaar voor de doelgroep jongeren (4-25 jaar) met NAH echter, practice based.

4.4. LOGOPEDIE

DIAGNOSTIEK EN INTERVENTIES

STROOMSCHEMA DIAGNOSTIEK LOGOPEDIE // LEEFTIJD T/M 18 JAAR

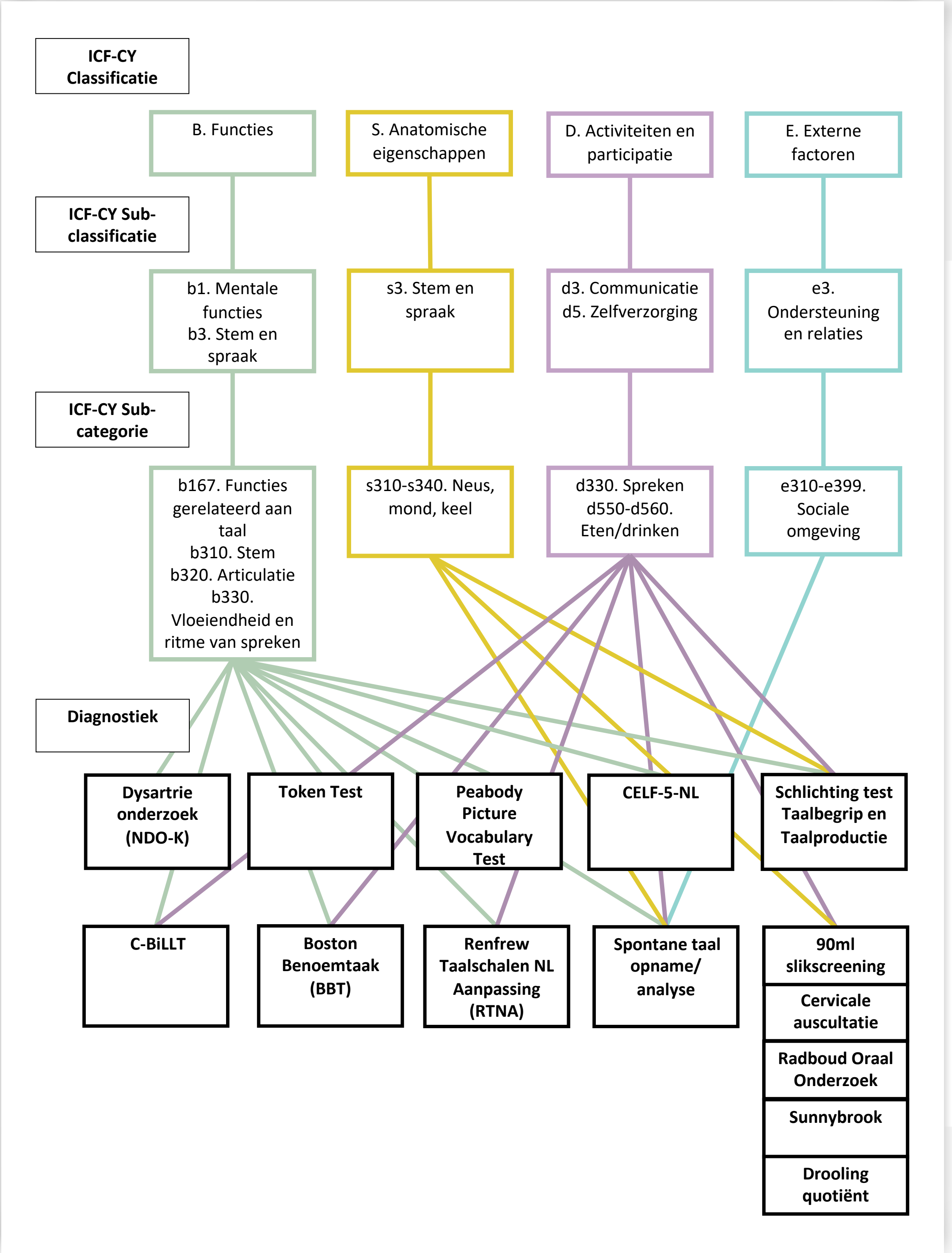

1

DIAGNOSTIEK

DYSARTRIE ONDERZOEK (NDO-K)

ICF-niveau

Functie/anatomie: stem en spraak

WAT MEET DEZE TEST PRIMAIR	Met de NDO kan de diagnose dysartrie, het type dysartrie en de ernst van de stoornis worden vastgesteld. Daarnaast onderscheidt de NDO-dysartrie van afasie en spraakapraxie.<br>Het instrument bevat gestandaardiseerde spreekopdrachten, waarbij alle aspecten van de spraak (adem, fonatie, articulatie, nasale resonantie en prosodie) in kaart worden gebracht. Er zijn 6 spreektaken: spontane spraak, lezen van een standaard tekst, diadochokinese, glijtonen, roepen en maximale aanhoudingsduur.<br>De NDO is geschikt om af te nemen in de acute, de revalidatie en de chronische fase.
PROTOCOL/ HANDLEIDING	Te vinden op: Nederlands Dysartrie Onderzoek - Volwassenen/ Kinderen – Meetinstrumenten in de zorg (meetinstrumentenzorg.nl)<br>Te koop op: BSL Shop | Nederlandstalig Dysartrieonderzoek - Kinderen (NDO-K) complete set
LEEFTIJDSGROEP	Kinderen 4-12 jaar / adolescenten 13-17 jaar.
TYPE LETSEL	Traumatisch: licht, matig, ernstig, Niet-traumatisch.
TIMING	Bij start, tijdens en na MSR.
DOEL VAN INZETTEN	De NDO is geschikt om af te nemen in de acute, de revalidatie en de chronische fase, maar niet diagnose specifiek. Het is een diagnostisch instrument en om te evalueren.
LITERATUUR EN PUBLICATIES	The Radboud Dysarthria Assessment:<br>Development and Clinimetric Evaluation.<br>https://pubmed.ncbi.nlm.nih.gov/29393211/
NORMWAARDEN BESCHIKBAAR	Ja, voor NDO-K zijn er Nederlandse normwaarden voor de taken: diadochokinese, maximale fonatieduur, maximale luidheid, range melodisch bereik.
NORMWAARDEN	Ja, voor NDO-k Nederlandse kinderen van 4-18 jaar.
CONCLUSIES	Evidentie gevonden, bij kinderen met dysartie<br>Bruikbaar voor de doelgroep jongeren (4-25 jaar) met NAH echter, practice based.

2

DIAGNOSTIEK

TOKEN TEST

ICF-niveau

Functie/anatomie: mentaal en zenuwstelsel

**WAT MEET DEZE TEST PRIMAIR**	Met de Token Test kan gemeten worden of een jongere moeite heeft met het begrijpen van lange zinnen en/of syntactisch complexe zinnen. Tevens kan de test gebruikt worden voor differentiaaldiagnostiek (wel/geen afasie).
**PROTOCOL/ HANDLEIDING**	Het scoreformulier wordt niet meer uitgegeven en zal binnenkort beschikbaar worden gesteld op de pagina van de landelijke werkgroep kinderafasie: https://www.afasienet.com/professionals/diagnostiek-en-therapie/kinderafasie/literatuur-verworven-kinderafasie/
**LEEFTIJDSGROEP**	Kinderen 6-12 jaar / adolescenten 13-16 jaar.
**TYPE LETSEL**	Traumatisch: licht, matig, ernstig, Niet-traumatisch.
**TIMING**	Bij start, tijdens en na MSR.
**DOEL VAN INZETTEN**	Diagnostisch en evaluatief.
**LITERATUUR EN PUBLICATIES**	Normative data of 300 Dutch-speaking children on the Token Test. https://doi.org/10.1080/02687030701722251
**NORMWAARDEN BESCHIKBAAR**	Ja, zie literatuur.
**NORMWAARDEN**	Ja, voor kinderen van 6-16 jaar.
**CONCLUSIES**	Bruikbaar voor de doelgroep jongeren (4-25 jaar) met NAH echter, practice based.

3

DIAGNOSTIEK

PEABODY PICTURE VOCABULARY TEST

ICF-niveau

Functie/anatomie: mentaal en zenuwstelsel

**WAT MEET DEZE TEST PRIMAIR**	Het testen van receptieve kennis van de woordenschat.
**PROTOCOL/ HANDLEIDING**	Te vinden en te koop op: PPVT-III-NL | Peabody Picture Vocabulary Test-III-NL - Pearson Clinical & Talent Assessment
**LEEFTIJDSGROEP**	Hele leeftijdsrange.
**TYPE LETSEL**	Traumatisch: licht, matig, ernstig, Niet-traumatisch.
**TIMING**	Bij start, tijdens en na MSR.
**DOEL VAN INZETTEN**	Diagnostisch/Evaluatief/Normatief.
**LITERATUUR EN PUBLICATIES**	The Peabody picture vocabulary test as a pre-screening tool for global cognitive functioning in childhood brain tumor survivors. https://pubmed.ncbi.nlm.nih.gov/21225316/
**NORMWAARDEN BESCHIKBAAR**	Ja, in Nederland.
**NORMWAARDEN**	Ja, zie bovenstaande literatuur.
**CONCLUSIES**	Evidentie gevonden, bij oncologische patiënten en patiënten met lage IQ's.<br>Bruikbaar voor de doelgroep jongeren (4-25 jaar) met NAH echter, practice based.

4

DIAGNOSTIEK

CLINICAL EVALUATION OF LANGUAGE-FUNDAMENTALS VIERDE/VIJFDE EDITIE- NEDERLANDSTALIGE VERSIE - CELF PRESCHOOL-2-NL, CELF-4-NL, CELF-5-NL

ICF-niveau

Functie/anatomie: mentaal en zenuwstelsel

Activiteiten/participatie: communicatie

(pragmatiekprofiel/observatieschaal)

WAT MEET DEZE TEST PRIMAIR	Het identificeren, diagnosticeren en opvolgen van taal- en communicatiestoornissen bij jongeren.<br>De CELF meet mondelinge expressieve (productieve) en receptieve (passieve) taalvaardigheid binnen verschillende domeinen: syntaxis, morfologie, semantiek en pragmatiek.
PROTOCOL/ HANDLEIDING	Te vinden op: https://www.pearsonclinical.nl<br>Te koop op: Celf-5-NL - Pearson Clinical & Talent Assessment<br>Cursus beschikbaar: CELF-5-NL training - Pearson Clinical & Talent Assessment
LEEFTIJDSGROEP	CELF Preschool-2-NL : 3 t/m 6 jaar<br>CELF-4-NL/CELF-5-NL: 5 t/m 18 jaar
TYPE LETSEL	Traumatisch: licht, matig, ernstig, Niet-traumatisch.
TIMING	Bij start, tijdens en na MSR.
DOEL VAN INZETTEN	Diagnostisch/Evaluatief/normatief.
LITERATUUR EN PUBLICATIES	Internet-based telehealth assessment of language using the CELF-4.<br>https://pubmed.ncbi.nlm.nih.gov/20421616/
NORMWAARDEN BESCHIKBAAR	Ja, Nederland.
NORMWAARDEN	Ja, zie bovenstaande literatuur.
CONCLUSIES	Evidentie gevonden, bij schoolgaande jongeren met NAH.<br>Bruikbaar voor de doelgroep jongeren (4-25 jaar) jaar met NAH echter, practice based.

5

DIAGNOSTIEK

SCHLICHTING TEST VOOR TAALBEGRIP EN TAALPRODUCTIE

ICF-niveau

Functie/anatomie: mentaal en zenuwstelsel

WAT MEET DEZE TEST PRIMAIR	De Schlichting test voor taalproductie meet de ontwikkeling van de actieve woordenschat, de grammaticale productie, de fonologische verwerkingsvaardigheid, de narratieve vaardigheden en het fonologisch werkgeheugen.
PROTOCOL/ HANDLEIDING	BSL Shop | Schlichting Test voor Taalbegrip<br>BSL Shop | Schlichting Test voor Taalproductie
LEEFTIJDSGROEP	Kinderen 4-12 jaar.
TYPE LETSEL	Traumatisch: licht, matig, ernstig, Niet-traumatisch.
TIMING	Bij start, tijdens en na MSR.
DOEL VAN INZETTEN	Diagnostisch/Evaluatief/normatief.
LITERATUUR EN PUBLICATIES	Schlichting Test voor Taalbegrip: handleiding. Houten: Bohn Stafleu van Loghum; 2010.
NORMWAARDEN BESCHIKBAAR	Ja.
NORMWAARDEN	Ja, zie bovenstaande literatuur.
CONCLUSIES	Evidentie gevonden bij jonge kinderen.<br>Bruikbaar voor de doelgroep jongeren (4-25 jaar) met NAH echter, practice based.

6

DIAGNOSTIEK

COMPUTER BASED INSTRUMENT FOR LOW MOTOR LANGUAGE TESTING (C-BILLT)

ICF-niveau

Functie/anatomie: mentaal en zenuwstelsel

WAT MEET DEZE TEST PRIMAIR	Een computer gebaseerde taalbegripstest voor niet of nauwelijks sprekende kinderen met (ernstige) motorische beperkingen.
PROTOCOL/ HANDLEIDING	Cursus – C-BiLLT Nederland<br>Info: About the C-BiLLT – C-BiLLT (c-billt.com)
LEEFTIJDSGROEP	1-7 jaar en ook voor ouderen met meervoudige beperking.
TYPE LETSEL	Traumatisch: licht, matig, ernstig, Niet-traumatisch.
TIMING	Bij start, tijdens en na MSR.
DOEL VAN INZETTEN	Observatief.
LITERATUUR EN PUBLICATIES	Reliability and validity of the C-BiLLT: a new instrument to assess comprehension of spoken language in young children with cerebral palsy and complex communication needs.<br>https://pubmed.ncbi.nlm.nih.gov/24948533/
NORMWAARDEN BESCHIKBAAR	Ja.
NORMWAARDEN	Ja.
CONCLUSIES	Voor kinderen tussen de 1.5 en 8 jaar met ernstige beperkingen en cerebrale parese. Bruikbaar voor doelgroep jongeren (4-25 jaar) met NAH echter, practice based.

7

DIAGNOSTIEK

BOSTON BENOEMTAAK (BBT)

ICF-niveau

Functie/anatomie: mentaal en zenuwstelsel

**WAT MEET DEZE TEST PRIMAIR**	Meetinstrument voor het vaststellen van woordvindstoornissen.
**PROTOCOL/ HANDLEIDING**	Nieuw: de Nederlandse Benoem Test (NBT) Diagnostiek- en behandelmateriaal, Nieuws voor professionals - AfasieNet<br>Te koop op: BSL Shop | Nederlandse Benoem Test NBT - complete set<br>Cursus beschikbaar: nee.
**LEEFTIJDSGROEP**	Adolescenten 13-17 jaar, jongvolwassenen 18-25 jaar.
**TYPE LETSEL**	Traumatisch: licht, matig, ernstig, Niet-traumatisch.
**TIMING**	Bij start en na MSR.
**DOEL VAN INZETTEN**	Diagnostisch/Evaluatief/normatief/discriminatief.
**LITERATUUR EN PUBLICATIES**	Effects of age, education, and living environment on Boston Naming Test performance.<br>https://pubmed.ncbi.nlm.nih.gov/8558882/<br>Development of a short form of the Boston naming test for individuals with aphasia.<br>https://pubmed.ncbi.nlm.nih.gov/21173387/
**NORMWAARDEN BESCHIKBAAR**	Ja.
**NORMWAARDEN**	Ja, zie bovenstaande literatuur.
**CONCLUSIES**	Evidentie gevonden, bij volwassenen met NAH.<br>Bruikbaar voor de doelgroep jongeren (4-25 jaar) met NAH echter, practice based.

8

DIAGNOSTIEK

RENFREW TAALSCHALEN NEDERLANDSE AANPASSING (RTNA)

ICF-niveau

Functie/anatomie: mentaal en zenuwstelsel

WAT MEET DEZE TEST PRIMAIR	Het doel is het onderzoeken van de kwaliteit van informatieoverdracht. Er zijn 3 taalschalen namelijk woordvinding woordenschat test, actieplaten test, busverhaal test. De taalmodaliteiten die onderzocht worden zijn fonologie, semantiek, morfologie, pragmatiek.
PROTOCOL/ HANDLEIDING	Te koop en info te vinden op: Renfrew Taalschalen Nederlandse Aanpassing (RTNA) (k2-publisher.nl)
LEEFTIJDSGROEP	Kinderen 4-12 jaar.
TYPE LETSEL	Traumatisch: licht, matig, ernstig, Niet-traumatisch.
TIMING	Bij start en na MSR.
DOEL VAN INZETTEN	Observatief.
LITERATUUR EN PUBLICATIES	The diagnostic and predictive validity of the Renfrew Bus Story. https://pubmed.ncbi.nlm.nih.gov/17890518/
NORMWAARDEN BESCHIKBAAR	Ja.
NORMWAARDEN	Ja, zie bovenstaande literatuur.
CONCLUSIES	Niet betrouwbaar voor doelgroep jongeren (4-25 jaar) met NAH, practice based.

9

DIAGNOSTIEK

SPONTANE TAAL OPNAME/ANALYSE

ICF-niveau

Activiteiten/participatie: communicatie

WAT MEET DEZE TEST PRIMAIR	Indruk van taalgebruiksaspecten in de spontane communicatie.
PROTOCOL/ HANDLEIDING	Document gestandaardiseerde beschrijving spontane taal bij VKA is beschikbaar: Verworven kinderafasie (VKA) - AfasieNet
LEEFTIJDSGROEP	Kinderen 4-12 jaar / adolescenten 13-17 jaar / jongvolwassenen 18-25 jaar.
TYPE LETSEL	Traumatisch: licht, matig, ernstig, Niet-traumatisch.
TIMING	Bij start, tijdens en na MSR.
DOEL VAN INZETTEN	Observatief/Evaluatief.
LITERATUUR EN PUBLICATIES	Nee.
NORMWAARDEN BESCHIKBAAR	Nee.
NORMWAARDEN	Nee.
CONCLUSIES	Geen evidentie (onderbouwing) gevonden. Brukkbaar voor de doelgroep jongeren (4-25 jaar) met NAH echter, practice based.

10

DIAGNOSTIEK

SLIKSCREENING 90ML (WATERSLIKTEST)

ICF-niveau

Functie/anatomie: spijsvertering, metabool, hormonen

WAT MEET DEZE TEST PRIMAIR	Het in kaart brengen van aanwezig- of afwezigheid van slikproblematiek (dysfagie).
PROTOCOL/ HANDLEIDING	Te vinden op: Richtlijn Orofaryngeale dysfagie - PDF Gratis download (docplayer.nl)<br>Screeningsmethode voor dysfagie - Richtlijn - Richtlijnendatabase
LEEFTIJDSGROEP	Kinderen 4-12 jaar / adolescenten 13-17 jaar / jongvolwassenen 18-25 jaar.
TYPE LETSEL	Traumatisch: licht, matig, ernstig, Niet-traumatisch.
TIMING	Bij start, tijdens en na MSR.
DOEL VAN INZETTEN	Screenend.
LITERATUUR EN PUBLICATIES	Clinical utility of the 3-ounce water swallow test.<br>https://pubmed.ncbi.nlm.nih.gov/18058175/
NORMWAARDEN BESCHIKBAAR	Ja, normwaarden zijn: 'negatief' d.w.z. geen verslikken, 'positief' is verslikken.
NORMWAARDEN	Ja, vanaf 4 jaar.
CONCLUSIES	Evidentie gevonden, bij volwassenen en kinderen met slikproblematiek.<br>Bruikbaar voor de doelgroep jongeren (4-25 jaar) met NAH echter, practice based.

11

DIAGNOSTIEK

CERVICALE AUSCULTATIE

ICF-niveau

Functie/anatomie: spijsvertering, metabool, hormonen

WAT MEET DEZE TEST PRIMAIR	Het luisteren met de stethoscoop naar de slik is waardevol binnen de logopedische diagnostiek en heeft toegevoegde waarde bij het uitvoeren van de therapie. Het auscultеren observeert de coördinatie van het slikken en ademhalen.
PROTOCOL/ HANDLEIDING	Cervicale auscultatie: Workshop Cervicale Auscultatie - Radboudumc
LEEFTIJDSGROEP	Kinderen 4-12 jaar / adolescenten 13-17 jaar / jongvolwassenen 18-25 jaar.
TYPE LETSEL	Traumatisch: licht, matig, ernstig, Niet-traumatisch.
TIMING	Bij start, tijdens en na MSR.
DOEL VAN INZETTEN	Observatief/Evaluatief/ Diagnostiek.
LITERATUUR EN PUBLICATIES	Nee.
NORMWAARDEN BESCHIKBAAR	Nee.
NORMWAARDEN	Nee.
CONCLUSIES	Bruikbaar voor de doelgroep jongeren (4-25 jaar) met NAH echter, practice based.

12

DIAGNOSTIEK

RADBOUD ORAAL ONDERZOEK

ICF-niveau

Functie/anatomie: bewegingssysteem

WAT MEET DEZE TEST PRIMAIR	Het Oraal Onderzoek is een observatie-instrument om systematisch stoornissen in de orofaciale en orofaryngeale sensomotoriek te documenteren.
PROTOCOL/ HANDLEIDING	Te vinden via: Oraal onderzoek - hersenzenuwen - motoriek – Radboudumc.
LEEFTIJDSGROEP	Kinderen 4-12 jaar / adolescenten 13-17 jaar / jongvolwassenen 18-25 jaar.
TYPE LETSEL	Traumatisch: licht, matig, ernstig, Niet-traumatisch.
TIMING	Bij start, tijdens en na MSR.
DOEL VAN INZETTEN	Observatief.
LITERATUUR EN PUBLICATIES	Nee.
NORMWAARDEN BESCHIKBAAR	Nee.
NORMWAARDEN	Nee.
CONCLUSIES	Bruikbaar voor de doelgroep jongeren (4-25 jaar) met NAH echter, practice based.

13

DIAGNOSTIEK

SUNNYBROOK

ICF-niveau

Functie/anatomie: bewegingssysteem

WAT MEET DEZE TEST PRIMAIR	Sunnybrook scale is een reeks van gelaatsbewegingen waarbij zowel in rust als bij beweging de symmetrie (en mate van beweeglijkheid) van het gelaat wordt geëvalueerd door een onderzoeker.
PROTOCOL/ HANDLEIDING	Sunnybrook: Sunnybrook facial grading system: reliability and criteria for grading - PubMed (nih.gov)
LEEFTIJDSGROEP	Hele leeftijdsrange.
TYPE LETSEL	Traumatisch: licht, matig, ernstig, Niet-traumatisch.
TIMING	Bij start, tijdens en na MSR.
DOEL VAN INZETTEN	Observatief/ Evaluatief/Diagnostiek.
LITERATUUR EN PUBLICATIES	Sunnybrook facial grading system: reliability and criteria for grading. https://pubmed.ncbi.nlm.nih.gov/20422701/
NORMWAARDEN BESCHIKBAAR	Nee.
CONCLUSIES	Geen evidentie (onderbouwing) gevonden voor NAH doelgroep. Bruikbaar voor de doelgroep jongeren (4-25jaar) met NAH als er sprake is van mondfunctiestoornissen echter, practice based.

14

DIAGNOSTIEK

DROOLING QUOTIËNT

ICF-niveau

Functie/anatomie: spijsvertering, metabool, hormonen

WAT MEET DEZE TEST PRIMAIR	Nauwkeurige beoordeling van de ernst van het kwijlen met het kwijlquotiënt van vijf minuten bij kinderen met ontwikkelingsstoornissen.
PROTOCOL/ HANDLEIDING	Droolingquotient: karen_van_hulst_-_complete_thesis_lores.pdf (revalidatiegeneeskunde.nl)
LEEFTIJDSGROEP	Kinderen 4-12 jaar.
TYPE LETSEL	Mogelijk bij Traumatisch: licht, matig, ernstig, Niet-traumatisch.
TIMING	Bij start, tijdens en na MSR.
DOEL VAN INZETTEN	Observatief.
LITERATUUR EN PUBLICATIES	Nee.
NORMWAARDEN BESCHIKBAAR	Nee.
NORMWAARDEN	Nee.
CONCLUSIES	Bruikbaar voor de doelgroep jongeren (4-25 jaar) met NAH echter, practice based.

AANVULLINGEN

Onderstaande diagnostiek t/m 18 jaar is mogelijk ook geschikt voor NAH.

- Fonologisch onderzoek (bijv. Hodson en Paden, Metaphon)
- Dyslexie screening test
- 1-minuut-leestest

STROOMSCHEMA DIAGNOSTIEK LOGOPEDIE // LEEFTIJD VANAF 18 JAAR

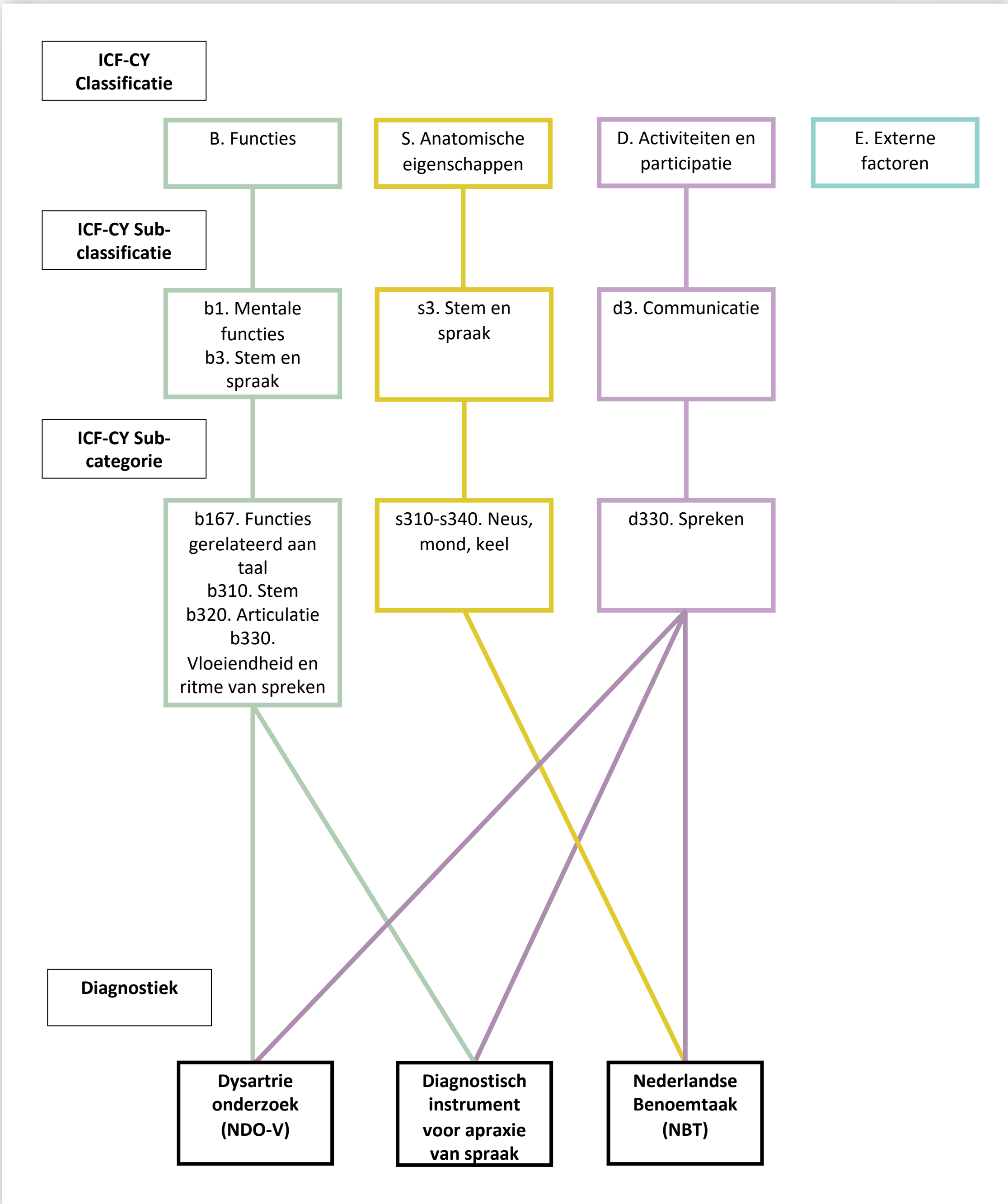

1

DIAGNOSTIEK

DYSARTRIE ONDERZOEK (NDO-V)

ICF-niveau

Functie/anatomie: stem en spraak

**WAT MEET DEZE TEST PRIMAIR**	Met de NDO kan de diagnose dysartrie, het type dysartrie en de ernst van de stoornis worden vastgesteld. Daarnaast onderscheidt de NDO-dysartrie van afasie en spraakapraxie.<br>Het instrument bevat gestandaardiseerde spreekopdrachten, waarbij alle aspecten van de spraak (adem, fonatie, articulatie, nasale resonantie en prosodie) in kaart worden gebracht. Er zijn 6 spreektaken: spontane spraak, lezen van een standaard tekst, diadochokinese, glijtonen, roepen en maximale aanhoudingsduur.<br>De NDO is geschikt om af te nemen in de acute, de revalidatie en de chronische fase.
**PROTOCOL/ HANDLEIDING**	Te vinden op: Nederlands Dysartrie Onderzoek - Volwassenen/ Kinderen – Meetinstrumenten in de zorg (meetinstrumentenzorg.nl)<br>Te koop op: BSL Shop | Nederlandstalig Dysartrieonderzoek - Kinderen (NDO-K) complete set
**LEEFTIJDSGROEP**	Jongvolwassenen 18-25 jaar.
**TYPE LETSEL**	Traumatisch: licht, matig, ernstig, Niet-traumatisch.
**TIMING**	Bij start, tijdens en na MSR.
**DOEL VAN INZETTEN**	Diagnostisch / evaluatief.
**LITERATUUR EN PUBLICATIES**	The Radboud Dysarthria Assessment: Development and Clinimetric Evaluation.<br>https://pubmed.ncbi.nlm.nih.gov/29393211/
**NORMWAARDEN BESCHIKBAAR**	Ja, voor NDO-K zijn er Nederlandse normwaarden voor de taken: diadochokinese, maximale fonatieduur, maximale luidheid, range melodisch bereik.
**NORMWAARDEN**	Ja, voor NDO-k Nederlandse kinderen van 4-18 jaar.
**CONCLUSIES**	Evidentie gevonden, bij kinderen met dysartie.<br>Bruikbaar voor de doelgroep jongeren (4-25 jaar) met NAH echter, practice based.

2

DIAGNOSTIEK

DIAGNOSTISCH INSTRUMENT VOOR APRAXIE VAN DE SPRAAK (DIAS)

ICF-niveau

Functie/anatomie: stem en spraak

WAT MEET DEZE TEST PRIMAIR	Apraxie van de spraak.
PROTOCOL/ HANDLEIDING	Te vinden op: Diagnostisch Instrument voor Apraxie van de Spraak (DIAS) - AfasieNet<br>Te koop op: BSL Shop | DIAS Diagnostisch instrument voor apraxie van de spraak<br>Cursus beschikbaar: www.onderwijs.umcg.nl
LEEFTIJDSGROEP	Jongvolwassenen 18-25 jaar.
TYPE LETSEL	Traumatisch: licht, matig, ernstig, Niet-traumatisch.
TIMING	Bij start, tijdens en na MSR.
DOEL VAN INZETTEN	Diagnostisch/Evaluatief.
LITERATUUR EN PUBLICATIES	Feiken & Jonkers, 2012 in Groningen.
NORMWAARDEN BESCHIKBAAR	Ja.
NORMWAARDEN	Ja.
CONCLUSIES	Evidentie gevonden, bij volwassenen met CVA.<br>Sommige taken wel bruikbaar voor de doelgroep (4-25 jaar) met NAH echter, practice based.

3

DIAGNOSTIEK

NEDERLANDSE BENOEMTAAK (NBT)

ICF-niveau

Functie/anatomie: mentaal en zenuwstelsel

WAT MEET DEZE TEST PRIMAIR	Meetinstrument voor het vaststellen van woordvindstoornissen en de ernst van de woordvindstoornissen.
PROTOCOL/ HANDLEIDING	Nieuw: de Nederlandse Benoem Test (NBT) Diagnostiek- en behandelmateriaal, Nieuws voor professionals - AfasieNet<br>Te koop op: BSL Shop | Nederlandse Benoem Test NBT - complete set<br>Cursus beschikbaar: nee.
LEEFTIJDSGROEP	Alleen normdata voor volwassenen (18+). Niet bedoeld voor of genormeerd voor kinderen en jongeren.
TYPE LETSEL	Alle typen letsel.
TIMING	Bij start, tijdens en na MSR.
DOEL VAN INZETTEN	Diagnostisch/Evaluatief/ bij volwassenen.
LITERATUUR EN PUBLICATIES	Handleiding (zie link boven) en diverse whitepapers met aanvullende gegevens: https://testweb.bsl.nl/whitepapers-nederlandse-benoem-test-nbt/
NORMWAARDEN BESCHIKBAAR	Ja.
NORMWAARDEN	Ja, zie bovenstaande literatuur.
CONCLUSIES	Geen evidentie voor kinderen en jongeren met NAH. Alleen voor volwassenen met een CVA. Bruikbaar voor de doelgroep jongeren (4-25 jaar) met NAH echter, practice based.

AANVULLINGEN

Onderstaande diagnostiek vanaf 18 jaar is mogelijk ook geschikt voor NAH.

- CAT (Comprehensive Aphasia Test)
- SAT (Semantische Associatie Test)
- Varianten van de Token Test (Akense Afasie Test versie 50 items), 61 itemsversie en Verkorte Token Test
- Psycholinguistic Assessments of Language Processing in Aphasia

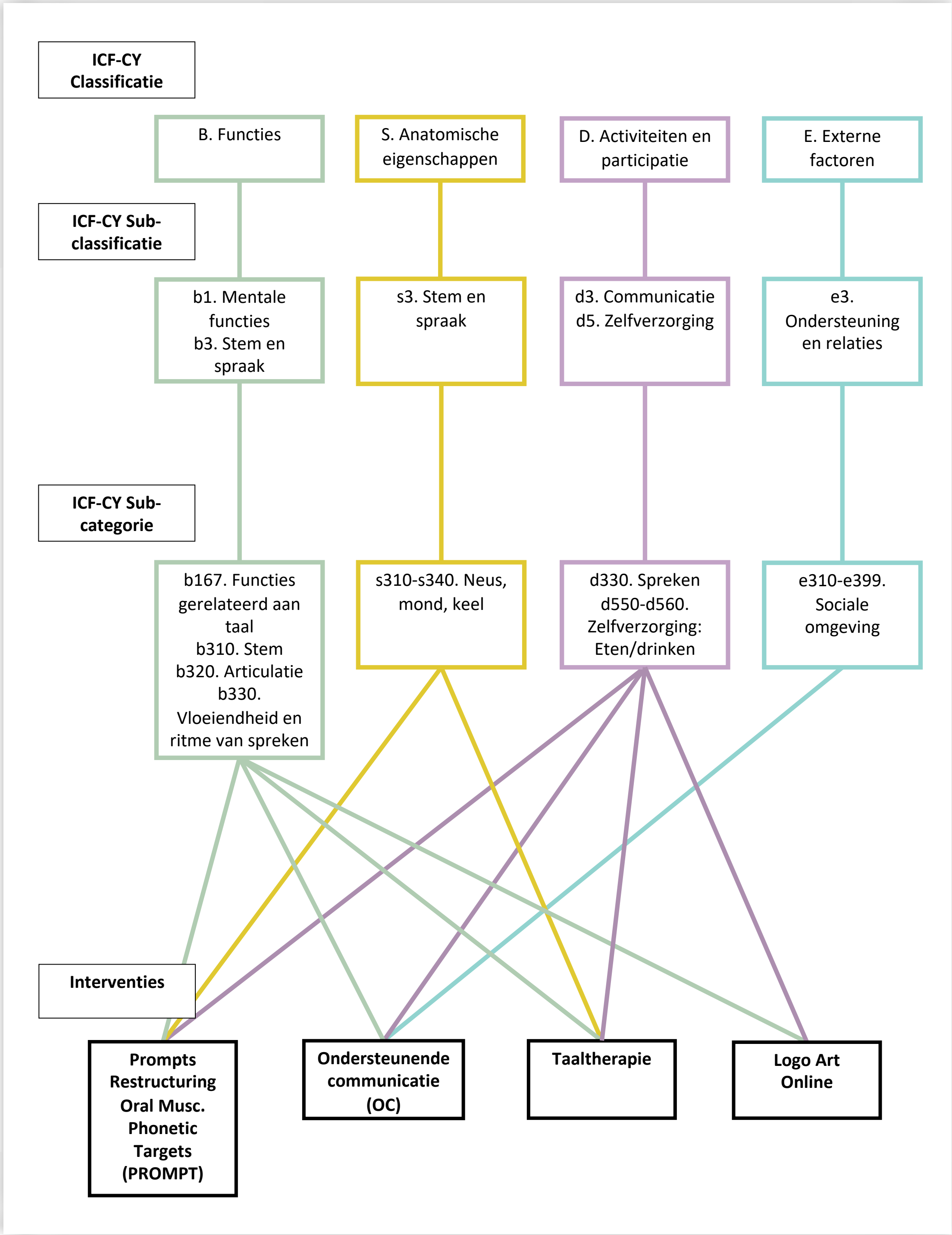
ICF-CY Classificatie
B. Functies
S. Anatomische eigenschappen
D. Activiteiten en participatie
E. Externe factoren
ICF-CY Sub-classificatie
b1. Mentale functies
b3. Stem en spraak
s3. Stem en spraak
d3. Communicatie
d5. Zelfverzorging
e3. Ondersteuning en relaties
ICF-CY Sub-categorie
b167. Functies gerelateerd aan taal
b310. Stem
b320. Articulatie
b330. Vloeiendheid en ritme van spreken
s310-s340. Neus, mond, keel
d330. Spreken
d550-d560. Zelfverzorging: Eten/drinken
e310-e399. Sociale omgeving
Interventies
Prompts Restructuring Oral Musc. Phonetic Targets (PROMPT)
Ondersteunende communicatie (OC)
Taaltherapie
Logo Art Online

1

INTERVENTIE

TAALTHERAPIE

ICF-niveau

ICF Activiteiten/participatie: communicatie

WAAR IS HET VOOR BEDOELD	Verbeteren taalvaardigheid en woordvinding.
TIMING	Bij start, tijdens en na MSR.
LEEFTIJDSGROEP	Kinderen 4-12 jaar / adolescenten 13-17 jaar / jongvolwassenen 18-25 jaar.
TYPE LETSEL	Traumatisch: licht, matig, ernstig, Niet-traumatisch.
BESCHRIJVING INTERVENTIE	Verbeteren van de taalvaardigheid.
PROTOCOL/ HANDLEIDING	Nvt.
LITERATUUR EN PUBLICATIES	Nvt.
CONCLUSIES	Geen evidentie (onderbouwing) gevonden.<br>Bruikbaar voor de doelgroep jongeren (4-25 jaar) met NAH echter, practice based.

2

INTERVENTIE

ONDERSTEUNENDE COMMUNICATIE (OC)

ICF-niveau

ICF Activiteiten/participatie: communicatie

**WAAR IS HET VOOR BEDOELD**	Ondersteunde Communicatie is het inzetten van alle mogelijke communicatievormen en- hulpmiddelen om communicatie toch mogelijk te maken bij patiënten die- vanwege(ernstige)stoornissen of beperkingen- belemmeringen ervaren in het waarnemen, verwerken, begrijpen en uiten van spraak, gebarentaal en schrift. Bij OC spelen de communicatiepartners een belangrijke rol: ook zij moeten de ondersteunende communicatievormen kunnen gebruiken.
**TIMING**	Bij start en tijdens MSR.
**LEEFTIJDSGROEP**	Kinderen 4-12 jaar / adolescenten 13-17 jaar / jongvolwassenen 18-25 jaar.
**TYPE LETSEL**	Traumatisch: licht, matig, ernstig, Niet-traumatisch.
**BESCHRIJVING INTERVENTIE**	inzetten van alle mogelijke communicatievormen en- hulpmiddelen om communicatie toch mogelijk te maken.
**PROTOCOL/ HANDLEIDING**	Nvt.
**LITERATUUR**	Nvt.
**CONCLUSIES**	Geen evidentie (onderbouwing) gevonden.<br>Bruikbaar voor de doelgroep jongeren (4-25 jaar) met NAH echter, practice based.

3

INTERVENTIE

PROMPTS FOR RESTRUCTURING ORAL MUSCULAR PHONETIC TARGETS (PROMPT)

ICF-niveau

ICF Functies/anatomie: stem en spraak

**WAAR IS HET VOOR BEDOELD**	Verbeteren van spraakachterstand bij kinderen en jongeren. Een tactiel-kinesthetische behandeltechniek voor de therapie van spraakmotorische problemen.
**TIMING**	Bij start, tijdens en na MSR.
**LEEFTIJDSGROEP**	Kinderen 4-12 jaar / adolescenten 13-17 jaar / jongvolwassenen 18-25 jaar.
**TYPE LETSEL**	Traumatisch: licht, matig, ernstig, Niet-traumatisch.
**BESCHRIJVING INTERVENTIE**	Verbeteren van spraakachterstand.
**PROTOCOL/ HANDLEIDING**	Te vinden op: https://promptinstitute.com/page/WIPforClincian
**LITERATUUR EN PUBLICATIES**	PROMPT intervention for children with severe speech motor delay: a randomized control trial.<br>https://pubmed.ncbi.nlm.nih.gov/32357364/<br>The assessment of fidelity in a motor speech-treatment approach. Speech, language and hearing.<br>https://pubmed.ncbi.nlm.nih.gov/26213623/
**CONCLUSIES**	Evidentie gevonden, bij jongeren met spraakachterstand en voor kinderen met CP.<br>Bruikbaar voor de doelgroep jongeren (4-18 jaar met NAH echter, practice based.

4

INTERVENTIE

LOGO ART ONLINE

ICF-niveau

ICF Activiteiten/participatie: communicatie

**WAAR IS HET VOOR BEDOELD**	Verbeteren communicatie en taalvaardigheid (online module).
**TIMING**	Bij start, tijdens en na MSR.
**LEEFTIJDSGROEP**	Kinderen 4-12 jaar / adolescenten 13-17 jaar / jongvolwassenen 18-25 jaar.
**TYPE LETSEL**	Traumatisch: licht, matig, ernstig, Niet-traumatisch.
**BESCHRIJVING INTERVENTIE**	Verbeteren communicatie en taalvaardigheid (online module).
**PROTOCOL/ HANDLEIDING**	Te vinden op: https://www.logo-art.com/
**LITERATUUR EN PUBLICATIES**	Nvt.
**CONCLUSIES**	Geen evidentie (onderbouwing) gevonden.<br>Bruikbaar voor de doelgroep jongeren (4-25 jaar) met NAH echter, practice based.

4.5. MAATSCHAPPELIJK WERK

DIAGNOSTIEK EN INTERVENTIES

STROOMSCHEMA DIAGNOSTIEK MAATSCHAPPELIJK WERK

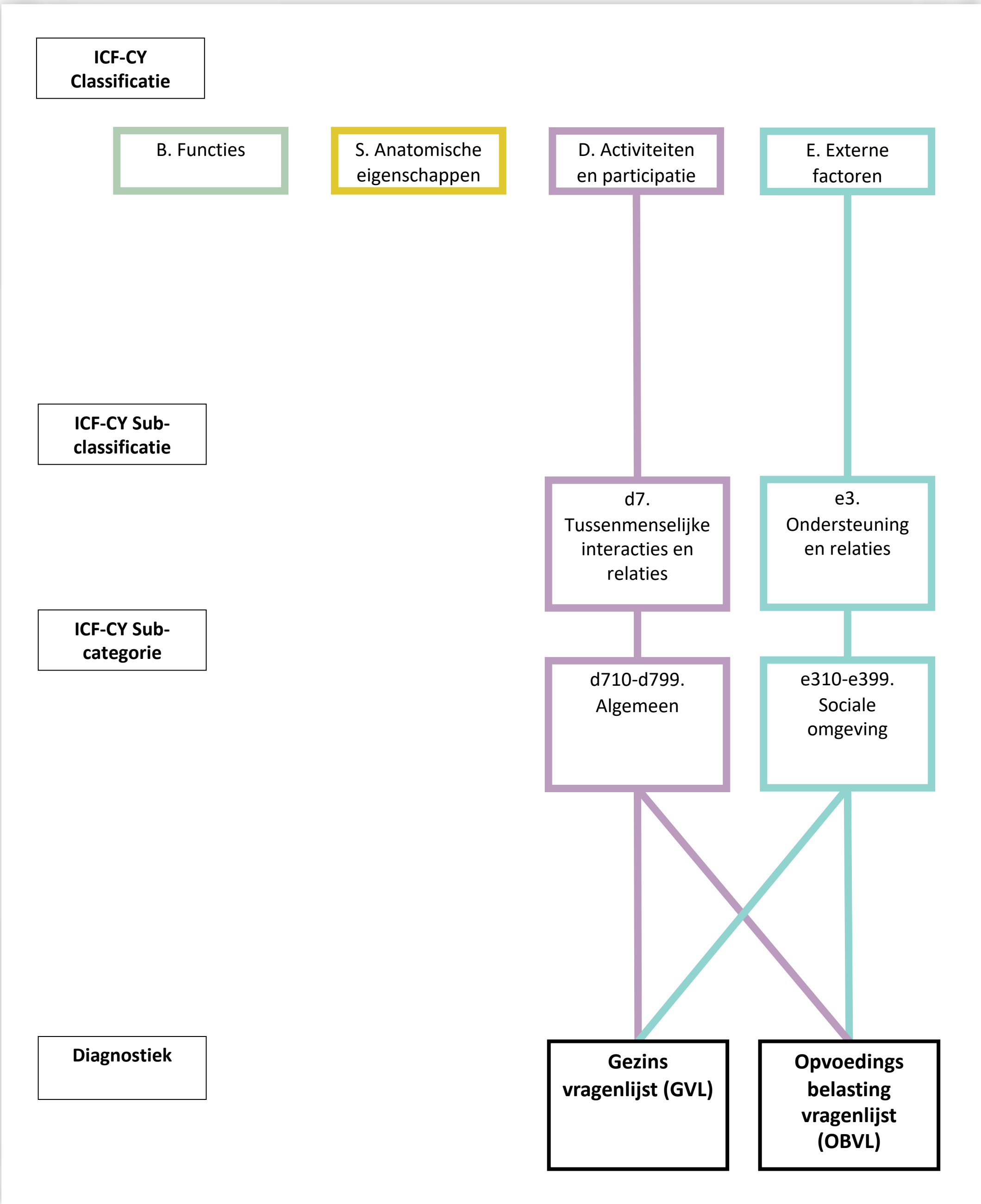

1

DIAGNOSTIEK

GEZINSVRAGENLIJST (GVL)

ICF-niveau

Menselijk functioneren' (ICF) Persoonlijke factoren, Communicatie, Tussenmenselijke interacties en relaties

**WAT MEET DEZE TEST PRIMAIR**	De kwaliteit van gezins- en opvoedingsomstandigheden. Interpretatie van de uitslagen mag alleen gedaan worden door psychodiagnostisch geschoolde personen.
**PROTOCOL/ HANDLEIDING**	Te vinden op: GVL-Vignet-2019.pdf (bergop.info) Te koop op: BSL Shop | GVL Gezinsvragenlijst Handleiding Beschikbaar via: http://www.bsl.nl/shop/gvl-gezinsvragenlijst-formulieren9789031353286.html. COTAN Documentatie: Gezinsvragenlijst, GVL. 2008. Beschikbaar via: http://www.cotandocumentatie.nl/test_details.php?id=647
**LEEFTIJDSGROEP**	Gezinnen met kinderen in de leeftijdscategorie van 4 tot en met 18 jaar.
**TYPE LETSEL**	Alle typen.
**TIMING**	Bij start en na MSR.
**DOEL VAN INZETTEN**	Diagnostisch.
**LITERATUUR EN PUBLICATIES**	Nvt.
**NORMWAARDEN BESCHIKBAAR**	Nvt.
**NORMWAARDEN**	Nvt.
**CONCLUSIES**	Bruikbaar voor de doelgroep jongeren (4-25 jaar) met NAH echter, practice based.

2

DIAGNOSTIEK

OPVOEDINGSBELASTING VRAGENLIJST (OBVL)

ICF-niveau

Externe factoren

(b.v. gezin, houding en ondersteuning)

**WAT MEET DEZE TEST PRIMAIR**	Screenen van opvoedingsbelasting.
**PROTOCOL/ HANDLEIDING**	Te vinden op: Opvoedingsbelasting Vragenlijst (OBVL) | Nederlands Jeugdinstituut (nji.nl)<br>Te koop op: Handleiding OBVL | Ad Vermulst, Gert Kroes, Ronald De Meyer, Linda Nguyen, Jan Willem Veerman (eburon.nl)
**LEEFTIJDSGROEP**	Ouders van jongeren van 4 tot en met 18 jaar.
**TYPE LETSEL**	Alle typen.
**TIMING**	Bij start, tijdens en na MSR.
**DOEL VAN INZETTEN**	Signalering en screening.
**LITERATUUR EN PUBLICATIES**	Nvt.
**NORMWAARDEN BESCHIKBAAR**	Nvt.
**NORMWAARDEN**	Nvt.
**CONCLUSIES**	Geen evidentie gevonden.<br>Wel bruikbaar voor de doelgroep jongeren (4-25 jaar) met NAH echter, practice based.

STROOMSCHEMA INTERVENTIES MAATSCHAPPELIJK WERK

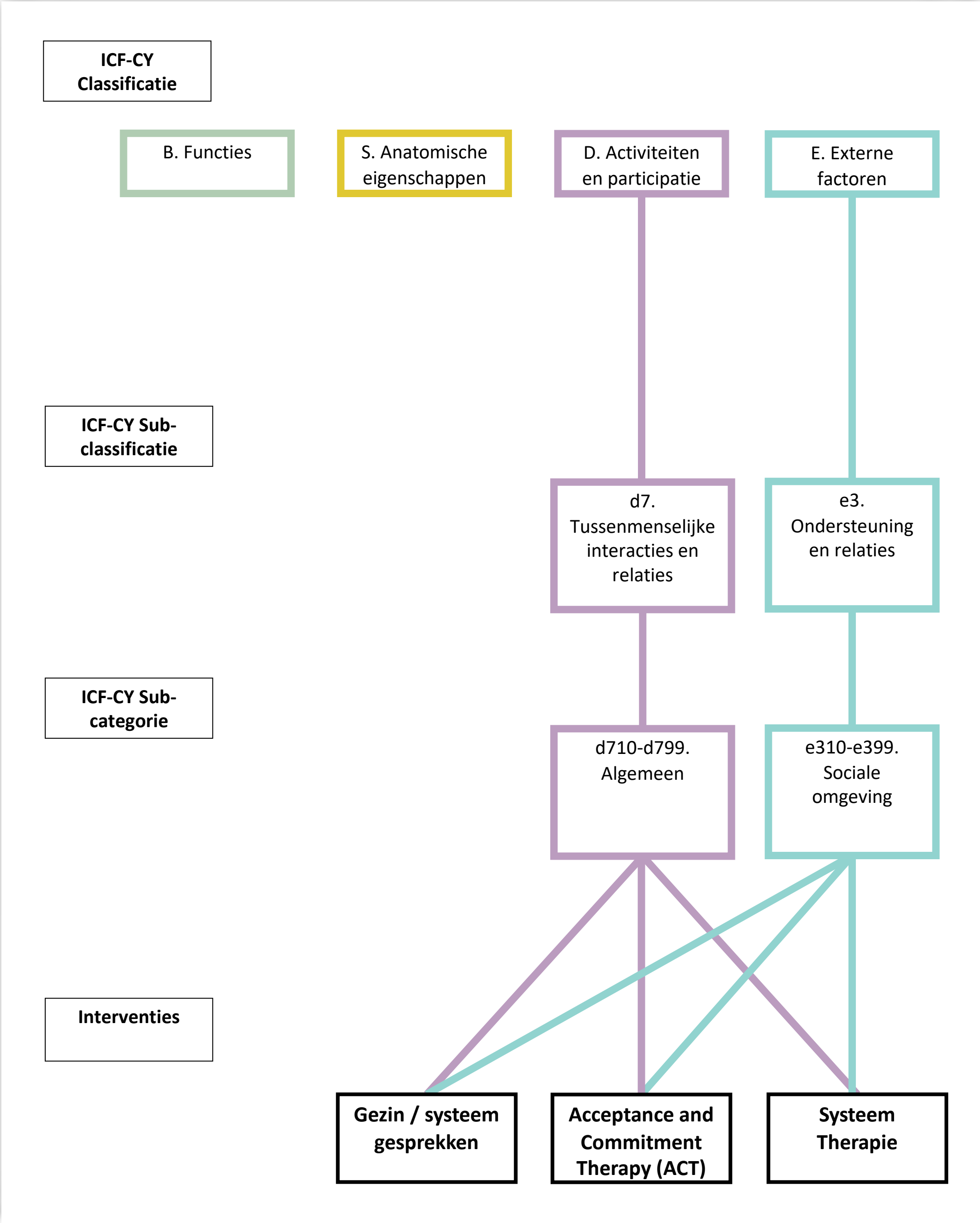

1

INTERVENTIE

GEZINSGERICHT WERKEN: GEZINSGESPREKKEN, OUDERGESPREKSGROEPEN, FAMILIEBIJEENKOMSTEN, BRUSJESGROEP. ZIE OOK INTERVENTIE PSYCHOLOGIE

ICF-niveau

Activiteiten/participatie: tussenmenselijke interacties en relaties

**WAAR IS HET VOOR BEDOELD**	De gezinsgerichte interventies kunnen zich op alle leden van het gezin richten en worden veel ingezet bij de behandeling van jongeren met NAH. Informatie over herstel na hersenletsel, het begrijpen van veranderingen in het gedrag door hersenletsel, erkenning voor het perspectief van alle gezinsleden kan helpen om de ontregeling die hersenletsel in gezinnen met zich meebrengt weer te reguleren. De gezinsgerichte interventies geven ouders/gezin input in hoe zij vanuit de gezinscontext het kind in zijn of haar herstel kunnen stimuleren.<br>In gezinsgericht werken zitten gradaties in de mate waarin ouders en gezin een rol en verantwoordelijkheid krijgen. Dit hangt af van de visie (b.v. klasgenoten/vrienden betrekken, aanbod broers en zussen, contextueel werken) en samenstelling (b.v. systeem- of speltherapeut) van het team.<br>In het team worden ook afspraken gemaakt over wie de contacten met school onderhoudt en of en hoe andere belangrijke personen (b.v. klasgenoten, vrienden) betrokken worden.
**TIMING**	Bij start en tijdens MSR.
**LEEFTIJDSGROEP**	Kinderen 4-12 jaar / adolescenten 13-17 jaar / jongvolwassenen 18-25 jaar.
**TYPE LETSEL**	Traumatisch: licht, matig, ernstig, Niet-traumatisch.
**BESCHRIJVING INTERVENTIE**	Eigen weg is een praktische en volledige ondersteuningsroute voor het opvoeden van jongeren met hersenletsel. Het is gebaseerd op 'Hooi op je Vork, het ondersteuningsmodel voor volwassenen met niet-aangeboren hersenletsel.<br>De ondersteuningsroute voor jongeren is ontwikkeld in nauwe samenwerking met ouders en deskundigen op het gebied van niet-aangeboren hersenletsel bij jongeren. De werkwijze omvat vijf stappen, langs vijf stations op de routekaart. Bij elk station sta je met elkaar stil bij bepaalde vragen. Daarna vervolg je de route weer.

**PROTOCOL/ HANDLEIDING**	Te vinden via: Patty van Belle en Judith Zadoks, Eigen Weg www.hooiopjevork.nl<br>Cursus: Scholing voor het gebruiken van dit programma via AXON leertrajecten.
**LITERATUUR EN PUBLICATIES**	Evidence-based systematic review of cognitive rehabilitation, emotional, and family treatment studies for children with acquired brain injury literature: From 2006 to 2017.<br>https://pubmed.ncbi.nlm.nih.gov/31671014/<br>Putting the pieces together: Prelimanary efficacy of a family problem solving intervention for children with traumatic brain injury.<br>https://pubmed.ncbi.nlm.nih.gov/16456392/<br>Direct clinician-delivered versus indirect family-supported rehabilitation of children with traumatic brain injury: a randomized controlled trial.<br>https://pubmed.ncbi.nlm.nih.gov/16175842/
**CONCLUSIES**	Evidentie gevonden, bruikbaar voor de doelgroep jongeren (4-25 jaar) met NAH.

2

INTERVENTIE

ACCEPTANCE AND COMMITMENT THERAPY (ACT)

ZIE OOK INTERVENTIE ERGOTHERAPIE

ICF-niveau

ICF Functie/anatomie: activiteiten/participatie

**WAAR IS HET VOOR BEDOELD**	Vorm van gedragstherapie die cliënten helpt om op een constructieve manier om te gaan met de obstakels die ze tegenkomen (Acceptance), zodat zij kunnen blijven investeren in de dingen die ze écht belangrijk vinden (Commitment). ACT bestaat uit zes verschillende processen/ vaardigheden. Doel van ACT is niet zozeer het reduceren van klachten, maar het ontwikkelen van persoonlijke veerkracht. ACT-vaardigheden in lijn met 'positieve gezondheid'. Kan aanvullend zijn opEMDR traumaverwerking/acceptatie.
**TIMING**	Tijdens MSR.
**LEEFTIJDSGROEP**	Kinderen 4-12 jaar / adolescenten 13-17 jaar / jongvolwassenen 18-25 jaar.
**TYPE LETSEL**	Traumatisch: licht, matig, ernstig, Niet-traumatisch.
**BESCHRIJVING INTERVENTIE**	Met ACT leert men jongeren gebruik te maken van zes procesgerichte vaardigheden die allemaal sterk met elkaar verbonden zijn. 6 stappen van de ACT; naar gedachten en gevoelens te leren kijken; acceptatie; zelf-als-context; defusie; waarden; toegewijde actie.
**PROTOCOL/ HANDLEIDING**	Boek: Acceptance and commitment therapy: The process and practice of mindful change, 2nd ed.<br>Cursus: www.act4kids.nl<br>Basiscursus ACT: www.actinactie.nl
**LITERATUUR EN PUBLICATIES**	Acceptance and Commitment Therapy bij kinderen en jongeren. G.M.Samsen& de Heus J.L. (2017) Houten, Bohn Stafleu van Loghum.<br>Hoe ACT kinderen en jongeren kan helpen.<br>Inez Buijck, in Gedragstherapie, jaargang 2021, nr. 3.<br>ACT voor het brein. Hersenproblematiek: verworven of ontwikkelingsvariant.<br>Francis Pascal- Claes, In: Signaal digitaal 2021 nr. 2 (digitale tijdschrift van Sig.vzw).
**CONCLUSIES**	Evidentie gevonden, bij jongvolwassenen. Bruikbaar voor de doelgroep jongeren (4-25 jaar) met NAH echter, practice-based.

3

INTERVENTIE

SYSTEEMTHERAPIE

ICF-niveau

ICF Functie/Anatomie: activiteiten/participatie

**WAAR IS HET VOOR BEDOELD**	Een systeemtherapeut kan werken aan rouw- en verwerkingsvragen, psycho-educatie, vragen over relaties, gedrag en communicatie in het gezin, maar ook vragen rond onderwijs, omgang in de klas etc.
**TIMING**	Tijdens MSR.
**LEEFTIJDSGROEP**	Kinderen 4-12 jaar / adolescenten 13-17 jaar / jongvolwassenen 18-25 jaar.
**TYPE LETSEL**	Traumatisch: licht, matig, ernstig, Niet-traumatisch.
**BESCHRIJVING INTERVENTIE**	Systeemtherapie heeft als uitgangspunt dat problemen of uitdagingen veelal ontstaan door de manier waarop wij met elkaar in relatie staan. Oftewel: het probleem van één kind in het gezin zegt iets over het gehele gezin. De therapeut gaat dan met het gehele systeem (hier gezin) aan de slag. Systeemtherapie is een van de vier grote stromingen in de psychotherapie (naast psychoanalyse, gedragstherapie, ervaringsgerichte therapie). Binnen systeemtheoretisch werken bestaan verschillende werkvormen (b.v. familieopstelling) en verbijzonderingen.
**PROTOCOL/ HANDLEIDING**	Scholing: onderscheid wordt gemaakt in systeemgericht werken (na MBO of HBO opleiding) en registratie als systeemtherapeut NVRG (post HBO/WO) De post-hbo-opleiding Contextuele Hulpverlening Basis duurt 2 jaar.
**LITERATUUR EN PUBLICATIES**	Qualitative research in family therapy: publication trends from 1980 to 1999.<br>https://pubmed.ncbi.nlm.nih.gov/11813369/<br>Training family therapists to work with children and families: a modified Delphi study.<br>https://pubmed.ncbi.nlm.nih.gov/15532255/<br>Contextual errors and failures in individualizing patient care. A multicentre study.<br>https://pubmed.ncbi.nlm.nih.gov/20643988/<br>Contextual errors in medical decision making: overlooked and understudied.<br>https://pubmed.ncbi.nlm.nih.gov/26630603/
**CONCLUSIES**	Evidentie gevonden, bij volwassenen.<br>Bruikbaar voor de doelgroep jongeren (4-25 jaar) met NAH echter, practice based.

4.6. ANDERE DISCIPLINES

Voor fysiotherapeuten, ergotherapeuten, logopedisten, psychologen en maatschappelijk werkers is per discipline het behandelprogramma vormgegeven waarbij de inhoud voor meerdere disciplines bruikbaar is. Er kunnen ook andere disciplines betrokken zijn bij de zorg voor de doelgroep, zoals:

- Diëtetiek
- Revalidatietechniek
- Bewegingstechnologie
- Bewegingsagogiek (sport, hydrotherapie) en psychomotore therapie
- Creatieve therapie
- Muziektherapie
- Verpleging en verzorging
- Speltherapie
- Haptotherapie
- Psychomotore therapie.

Deze disciplines hebben geen eigen beschrijving, niet omdat deze disciplines minder belangrijk zijn, maar omdat zij niet in alle centra voor MSR ingezet worden en er te weinig specifieke materialen (psycho-educatie) en methodes (diagnostiek en interventies) beschikbaar zijn waarover consensus met betrekking tot toepassing bestaat.

4.7. MATERIALEN VOOR PSYCHO-EDUCATIE

Voor jongeren, het gezin en de sociale omgeving is voorlichting een essentieel onderdeel gedurende de hele behandeling. Psycho-educatie betreft voorlichting over allerlei onderwerpen die met NAH te maken hebben en veelvuldig gebruikt worden tijdens de behandeling. In onderstaand schema staan de meest gebruikte vormen van psycho-educatie. Er staat bij elk voorbeeld gespecificeerd voor welke doelgroep het materiaal geschikt is. Ook staat er beschreven waar het materiaal te vinden is en wat de eventuele kosten zijn. Dit overzicht wordt periodiek geüpdatet door de werkgroep 'Hersenletsel en Jeugd' (HeJ).

MATERIAAL	SPECIFICATIE: GESCHIKT VOOR	BRON EN BESCHIKBAARHEID
Boek:<br>"Ik hou nog steeds van appeltaart"	Ouders<br>Adolescenten (13-17 jaar)<br>Jongvolwassenen (18-25 jaar)	Bron: Uitgeverij Pica<br>Beschikbaarheid: te koop: 24,95
Folder:<br>"Hoe verder na traumatisch hersenletsel bij kinderen en jongeren"	Ouders<br>Adolescenten (13-17 jaar)<br>Jongvolwassenen (18-25 jaar)	Bron: Hersenletsel en Jeugd (HeJ), Vereniging van Revalidatieartsen (VRA), Hersenstichting<br>Beschikbaarheid: Gratis Folder (PDF)
Website:<br>hersenletseluitleg.nl	Ouders<br>Jongvolwassenen (18-25 jaar)	Bron: Hersenletseluitleg.nl 2013-2021 (i.s.m. project 'overprikkeling')<br>Beschikbaarheid: Gratis site + zoekfuncties
App:<br>Energie/activiteitenweger	Adolescenten (13-17 jaar)<br>Jongvolwassenen (18-25 jaar)	Bron: Verder met hersenletsel<br>Beschikbaarheid: App: Te koop 5,99 in app-store (iPhone) of PlayStore (Android)
Site:<br>breinstraat nl	Adolescenten (13-17 jaar<br>Jongvolwassenen (18-25 jaar)<br>Ouders en gezin, professionals	Bron: Breinstraat<br>Beschikbaarheid: Gratis account aan te maken voor jongeren en jongvolwassen.
Boek:<br>Speels brein	Ouders	Bron: 'Speels brein'<br>Beschikbaarheid: Boekje: te koop voor 19,75. Handleiding voor behandelaren: 3,99-4,99
Site:<br>Kinderneurologie.eu	Ouders<br>Professionals	Bron: Kinderneurologie<br>Beschikbaarheid: Gratis info op te zoeken.
Folder:<br>"Het NAH boekje voor onderwijs"	Professionals	Bron: Breinsupport<br>Beschikbaarheid: Gratis Folder (PDF)
Website:<br>Overprikkeling.com	Professionals	Bron: project overprikkeling 2013-2021 (ism Hersenletseluitleg.nl)<br>Beschikbaarheid: Gratis site+zoekfuncties
Folder:<br>Zorgstandaard Traumatisch Hersenletsel Kinderen & Jongeren	Professionals	Bron: Hersenstichting<br>Beschikbaarheid: Prijs: folder gratis te downloaden (Hardcopy: verzendkosten post € 1,50)
Boek:<br>"Brainstars "	Kinderen (4-12 jaar)<br>Adolescenten (13-17 jaar)<br>Ouders<br>Professionals<br>Algemene informatie	Bron: Vilans<br>Beschikbaarheid: boek te koop: €74,50

Boek:<br>“Mag ik ook ff”	Broers en zussen	Bron: Hersenstichting<br>Beschikbaarheid: Prijs: gratis (verzendkosten post € 2,95)
Boek:<br>“NAH niet altijd handig”	Kinderen (4-12 jaar)<br>Adolescenten (13-17 jaar)<br>Broers en zussen	Bron: Wegwijzer hersenletsel<br>Beschikbaarheid: te koop via syntaxmedia
Boek:<br>“Waarom heeft een krokodil zo’n platte kop”	Kinderen	Bron: Waarom heeft een krokodil zo’n platte kop<br>Beschikbaarheid: bibliotheek
Site:<br>“Afasienet.com”	Jongvolwassenen<br>(18-25 jaar)<br>Ouders<br>Professionals	Bron: Afasienet<br>Beschikbaarheid: gratis beschikbaar
Folder:<br>”Slaaptips voor kinderen en pubers”	Kinderen (4-12 jaar)<br>Adolescenten (13-17 jaar)<br>Ouders<br>Professionals	Bron:<br>Voor kinderen<br>Voor pubers
Boek:<br>“Elvin het vergeetachtige olifantje”	Kinderen (4-12 jaar)	Bron: Boek<br>Beschikbaarheid: te koop/te huur bibliotheek
PDF/Boek:<br>“Er lijkt niets met ons aan de hand maar dat is niet zo. Ons hoofd moet heel hard werken”	Ouders<br>Professionals<br>Algemene informatie	Bron: Zorg op maat<br>Beschikbaarheid: gratis beschikbaar
“Brain Blocks”	Kinderen (4-12 jaar)<br>Adolescenten (13-17 jaar)<br>Jongvolwassenen<br>(18-25 jaar)	Bron: Brain Blocks<br>Beschikbaarheid: basisset te koop voor: €125,-
Onderzoek/artikel: “Brains ahead! psycho-educatie voor licht hersenletsel”	Ouders<br>Professionals<br>Algemene informatie	Bron: Artikel<br>Beschikbaarheid: gratis beschikbaar
Boek:<br>“De puzzel van nah”	Ouders<br>Professionals	Bron: ‘De puzzel van NAH’<br>Beschikbaarheid: gratis beschikbaar
Boek:<br>“Bordje vol”	Ouders	Bron: Bordje Vol<br>Beschikbaarheid: te koop voor: €85,- tot €4050,- (voor de hele set)
Boek:<br>“Omgaan met hersenletsel”	Ouders<br>Professionals	Bron: Omgaan met hersenletsel<br>Beschikbaarheid: te koop voor: €36,-
Site/folder:<br>“Methode RIK (Revalidatie En Ik)”	Adolescenten (13-17 jaar)<br>Jongvolwassenen<br>(18-25 jaar)	Bron: Revalidatie en ik<br>Beschikbaarheid: CD-rom: €49,-
Boek:<br>De Zorgzame Giraffe, autobiografisch verhaal over Niet Aangeboren Hersenletsel	Ouders	Bron: De Zorgzame Giraffe<br>Beschikbaarheid: via uitgever/webshops
Film: “Ze zeggen dat ik zo veranderd ben”	Jongvolwassenen<br>(18-25 jaar)<br>Ouders<br>Professionals 5/56	Bron: Ze zeggen dat ik zo veranderd ben<br>Beschikbaarheid: te leen bij verschillende bibliotheken
Boek: “Volle Hoofden Boek (werkboek voor kinderen/ jongeren)”	Kinderen (4-12 jaar)<br>Adolescenten (13-17 jaar)	Bron: wij-spelen.nl<br>Beschikbaarheid: op verschillende webshops (prijs tussen de €29,- en €79,-)

5

5. BEHANDELPROGRAMMA - TRANSITIEFASE

De transitiefase naar volwassenheid (gekenmerkt door autonomie en zelfredzaamheid) wordt voor jongvolwassenen (14/18-25 jaar) met een beperking gezien als een risicofase.

De landelijke Kwaliteitsstandaard 'Jongeren in transitie van kinderzorg naar volwassenenzorg' geeft een belangrijke leidraad voor de zorg voor deze leeftijdsgroep. Een jongvolwassene bepaalt in vergelijking met kinderen meer zelf wat er gedaan moet worden, maar doet dit samen met zijn behandelaar als coach en krijgt instrumenten aangereikt om zelfmanagement en eigen regie te faciliteren. Dit kan en mag volgens de wet ook zonder inspraak van ouders. Een jongvolwassene maakt enkele, belangrijke overgangen door in de ontwikkeling van kind naar volwassenheid. Juist door NAH kunnen deze overgangen meer moeite kosten en kan specifieke ondersteuning nodig zijn.

In vijf centra voor MSR (Basalt, De Hoogstraat revalidatie, Libra Revalidatie & Audiologie, Reade, en Rijndam) is daarom een specifiek aanbod voor deze leeftijdsgroep (14 of 18-25 jaar) ontwikkeld. Hierin werken dezelfde disciplines als bij de kinderrevalidatie samen, maar worden andere accenten gelegd:

- Aanbod kan heel praktisch en direct op een ontwikkelingsstap gericht zijn, b.v. uit huis gaan, omgaan met geld, relaties en seksualiteit, start met werken.
- Indien van toepassing wordt met de opleidingsinstelling (VO, MBO, HBO, WO) gezocht naar faciliteiten op maat (o.a. aanwezigheid, tempo, toetsen, begeleiding) om het programma succesvol te kunnen volgen. Voor arbeidstoeleiding of re-integratie naar werk is in verschillende regio's specifiek aanbod ontwikkeld.

Aandachtspunten voor het vormgeven van de behandeling zijn:

- Betrekken en inzetten van leeftijdgenoten kan van groot belang zijn. In enkele centra (de Hoogstraat, Basalt) is hier een specifiek programma voor ontwikkeld.
- Leren van en delen met ervaringsdeskundigen is vaak een sterke behoefte;
- Zorg op afstand (eHealth, blended care) kan een oplossing zijn om in het volle dagprogramma ruimte voor behandeling en begeleiding te vinden.
- Het stimuleren van/ontwikkelen van de zelfstandigheid en eigen regie van jongeren waar mogelijk en indien nodig het bevorderen van het passend delen van verantwoordelijkheden tussen ouder/jongere.
- Inrichting en sfeer behandelomgeving, werkvormen en attitude professionals sluiten zoveel mogelijk aan bij belevingswereld van deze leeftijdsgroep.

6

6. REGIO-OVERSTIJGENDE, SPECIALISTISCHE CENTRA

In Nederland bestaan, naast het reguliere aanbod in de MSR zoals in dit gezamenlijk behandelprogramma uitgewerkt, enkele mogelijkheden om jongeren met NAH op indicatie te behandelen. Dit specifieke, regio-overstijgende aanbod is aanvullend op, en sluit aan bij het reguliere MSR-aanbod:

Daan Theeuwes Centrum (DTC)

Het Daan Theeuwes centrum is een focus kliniek voor intensieve neurorevalidatie aan jongvolwassenen in leeftijd tussen 16 en 35 jaar. Kernwoorden in de revalidatiebehandeling zijn: specialistische, intensieve en op functieherstel gerichte therapie in een interdisciplinaire setting.

Het DTC onderscheidt zich van andere volwassenrevalidatie afdelingen door enerzijds de focus te leggen alleen op revalidanten met NAH, op een jongvolwassen doelgroep en door zeer intensieve therapie te bieden, van gemiddeld 25 uur therapie per week. De therapie richt zich daarbij in eerste instantie op functie- en activiteitenniveau en pas later gericht op participatieniveau, waarbij compensatiestrategieën benut kunnen worden. Het DTC heeft als visie dat jongvolwassenen hun leven nog voor zich hebben, waardoor het langer en intensiever wil investeren in deze fase van de revalidatie, met de verwachting dat dit profijt oplevert in de rest van het leven.

De revalidatie is interdisciplinair georganiseerd en heeft zowel mogelijkheden voor klinische als poliklinische behandeling. Poliklinische revalidanten kunnen, mits zij ADL zelfstandig zijn en er geen verpleegkundige zorg meer noodzakelijk is, eventueel gebruik maken van "hotelbedden", om meerdere dagen achtereen per week te kunnen revalideren, zonder op en neer te hoeven reizen.

Vanwege de unieke clustering van jongvolwassen revalidanten met ernstig NAH komen er verwijzingen vanuit het gehele land, met name van de traumacentra met neurochirurgische mogelijkheden. Daarnaast worden regelmatig revalidanten uit andere revalidatiecentra doorverwezen, voor vervolgrevalidatie, vanwege de centrum specifieke mogelijkheden en/of wensen van revalidant zelf.

Vroege Intensieve Neurorevalidatie (VIN)

Bij Libra Revalidatie & Audiologie, locatie Leijpark te Tilburg krijgen onder andere jongeren die in een toestand van verlaagd bewustzijn verkeren het behandelprogramma Vroege Intensieve Neurorevalidatie aangeboden. De basis van dit VIN-programma is het actief aanbieden, structuren en coördineren van uiteenlopende multidisciplinaire behandelingen met een zo gangbaar mogelijk dagritme. De medische, paramedische en verpleegkundige behandelingen worden gecombineerd met systematische zintuiglijke en/of cognitieve stimulering en een intensieve begeleiding van het gezinssysteem.

Inclusiecriteria voor het VIN-programma zijn:

1. Een verlaagd bewustzijn langer dan 1 maand bestaand.
2. Medisch stabiel, dat wil zeggen geen (onbegrepen) koorts; niet zuurstofafhankelijk en geen infuus.
3. Minstens drie momenten in de week moet een naaste aanwezig kunnen zijn.
4. Moeten in staat zijn om het intensieve programma te volgen.

Exclusiecriteria zijn:

1. Progressief hersenletsel.
2. Beperkte levensverwachting bij co-morbiditeit.
3. Taalbarrière waarbij er geen tolk aanwezig kan zijn.

Het VIN-programma duurt 14 weken, waarvan twee weken observatie en 2x zes weken behandeling. Na 14 weken wordt iemand naar een passende plek uitgeplaatst. Als er nog sprake is van een verlaagd bewustzijn dan is een verpleeghuis voor langdurige intensieve neurorevalidatie (LIN) een passende plek. Er zijn drie verpleeghuizen die LIN bieden, te weten: Crabbenhoven te Dordrecht; Accolade te Bosch en Duin en WZH, Nieuw-Berkendael te Den Haag. Als iemand bij bewustzijn is gekomen kan iemand uitgeplaatst worden naar de MSR, een verpleeghuis, in een woonvorm (met specialisme NAH) of naar huis met langdurige zorg (WLZ).

Prinses Maxima Centrum (PMC)

Het PMC is een ziekenhuis in Utrecht met specialistische behandeling voor hersenaandoeningen ten gevolge van oncologische problematiek die ontstaan zijn voor de leeftijd van 18 jaar. Binnen het PMC ligt de focus op de oncologische behandeling en indien nodig verwijzen zij door naar, of werken zij samen met de MSR.

Netwerk Kind & NAH+

In opdracht van het ministerie van VWS is het Netwerk Kind & NAH+ opgericht. Dit netwerk heeft als doel om een landelijk zorglandschap te ontwikkelen voor 'jongeren met een complexe, intensieve en langdurige zorgvraag'. Gespecialiseerde zorg en behandeling met specifieke kennis en kunde is nodig om goede zorg te kunnen bieden die bijdraagt aan een optimale kwaliteit van leven van het kind en zijn gezin.

Het netwerk Kind&NAH+ heeft zes Regionale Expertise Centra (REC's) die ingebed zijn in een netwerk van ketenpartners in de regio, 2 Doelgroep Expertise Centra (DEC's) revalidatie, 1 DEC GGZ en een KC (Kennis Centrum Kind&NAH+) opgericht met daaraan gekoppeld een kennisnetwerk van andere organisaties ter versterking van wetenschap en kennisdeling.

De REC's bieden passende diagnostiek en aanvullende zorg en ondersteuning nabij de woonplek van het kind met NAH+ in afstemming met de NAH-coach die de zorg rondom het kind met NAH+ en zijn gezin (mee) coördineert.
De DEC's revalidatie bieden vanuit de medisch specialistische revalidatie extra expertise op het gebied van diagnostiek en behandeladvies.
Op basis van alle ontwikkelingen in de vakgebieden van revalidatie, neurologie en psychiatrie is een geïntegreerde aanpak voor deze doelgroep met NAH+ van groot belang.

7

7. REFERENTIES

ALGEMEEN

1. Hersenstichting. Zorgstandaard traumatisch hersenletsel kinderen & jongeren (herziening 2022). Hersenalliantie; 2016 / 2022.
2. Greenwald BD, Burnett DM, Miller MA. Congenital and acquired brain injury. 1. Brain injury: epidemiology and pathophysiology. *Arch Phys Med Rehabil* 2003; **84**(3 Suppl 1): S3-7.
3. A. Hijdra PK, R. Roos. Neurologie. Houten: Bohn Stafleu van Loghum; 2016.
4. Eilander HH, J. Klinische kinderneuropsychologie. 2011.
5. Jain S, Iverson LM. Glasgow Coma Scale. StatPearls. Treasure Island (FL); 2020.
6. Rakers SE, Timmerman ME, Scheenen ME, et al. Trajectories of Fatigue, Psychological Distress, and Coping Styles After Mild Traumatic Brain Injury: A 6-Month Prospective Cohort Study. *Arch Phys Med Rehabil* 2021; **102**(10): 1965-71 e2.
7. Allonsius F, de Kloet A, Bedell G, et al. Participation Restrictions among Children and Young Adults with Acquired Brain Injury in a Pediatric Outpatient Rehabilitation Cohort: The Patients' and Parents' Perspective. *Int J Environ Res Public Health* 2021; **18**(4).
8. Allonsius F, De Kloet AJ, Van Markus-Doornbosch F, et al. Parent-reported family impact in children and young adults with acquired brain injury in the outpatient rehabilitation setting. *Brain Inj* 2021: 1-11.
9. Allonsius F, van Markus-Doornbosch F, de Kloet AJ, Lambregts S, Vliet Vlieland T, van der Holst M. Fatigue in young patients with acquired brain injury in the rehabilitation setting: Categorizing and interpreting fatigue severity levels. *Dev Neurorehabil* 2022: 1-12.
10. van Markus-Doornbosch F, van der Holst M, de Kloet AJ, Vliet Vlieland TPM, Meesters JJL. Fatigue, Participation and Quality of Life in Adolescents and Young Adults with Acquired Brain Injury in an Outpatient Rehabilitation Cohort. *Dev Neurorehabil* 2020; **23**(5): 328-35.
11. Kleim JA, Jones TA. Principles of experience-dependent neural plasticity: implications for rehabilitation after brain damage. *J Speech Lang Hear Res* 2008; **51**(1): S225-39.
12. Maier M, Ballester BR, Verschure P. Principles of Neurorehabilitation After Stroke Based on Motor Learning and Brain Plasticity Mechanisms. *Front Syst Neurosci* 2019; **13**: 74.
13. Forsyth R. The difference rehabilitation can make after acquired brain injury. *Dev Med Child Neurol* 2022; **64**(1): 7.
14. Anaby D, Pozniak K. Participation-based intervention in childhood disability: a family-centred approach. *Dev Med Child Neurol* 2019; **61**(5): 502.
15. Braga LW, Da Paz AC, Ylvisaker M. Direct clinician-delivered versus indirect family-supported rehabilitation of children with traumatic brain injury: a randomized controlled trial. *Brain Inj* 2005; **19**(10): 819-31.
16. Neurologie NVv. Richtlijn opvang patiënten met licht traumatisch hoofd/hersenletsel. 2010.
17. Roelofsen EE, The BA, Beckerman H, Lankhorst GJ, Bouter LM. Development and implementation of the Rehabilitation Activities Profile for children: impact on the rehabilitation team. *Clin Rehabil* 2002; **16**(4): 441-53.
18. Rothstein JM, Echternach JL, Riddle DL. The Hypothesis-Oriented Algorithm for Clinicians II (HOAC II): a guide for patient management. *Phys Ther* 2003; **83**(5): 455-70.

REFERENTIES

DIAGNOSTIEK EN INTERVENTIES

1. Swaab HB, A. Hendriksen, J. König, C Klinische kinderneuropsychologie; 2011.
2. Spikman J. Handboek Traumatisch hersenletsel. 2021: p 177-91.
3. Laatsch L, Dodd J, Brown T, et al. Evidence-based systematic review of cognitive rehabilitation, emotional, and family treatment studies for children with acquired brain injury literature: From 2006 to 2017. Neuropsychol Rehabil 2020; 30(1): 130-61.
4. Laatsch L, Harrington D, Hotz G, et al. An evidence-based review of cognitive and behavioral rehabilitation treatment studies in children with acquired brain injury. J Head Trauma Rehabil 2007; 22(4): 248-56.
5. Manzoni M, Fernandez I, Bertella S, et al. Eye movement desensitization and reprocessing: The state of the art of efficacy in children and adolescent with post traumatic stress disorder. J Affect Disord 2021; 282: 340-7.
6. Wade SL, Michaud L, Brown TM. Putting the pieces together: preliminary efficacy of a family problem-solving intervention for children with traumatic brain injury. J Head Trauma Rehabil 2006; 21(1): 57-67.
7. Braga LW, Da Paz AC, Ylvisaker M. Direct clinician-delivered versus indirect family-supported rehabilitation of children with traumatic brain injury: a randomized controlled trial. Brain Inj 2005; 19(10): 819-31.
8. Samsen GMdH, J.L. Acceptance and Commitment Therapy bij kinderen en jongeren. Houten: Bohn Stafleu van Loghum; 2017.
9. Buijck I. Hoe ACT kinderen en jongeren kan helpen Gedragstherapie; 2021.
10. Claes FP. ACT voor het brein. Hersenproblematiek: verworven of ontwikkelingsvariant. Signaal digitaal 2021.
11. Dua K, Lancaster TP, Abzug JM. Age-dependent Reliability of Semmes-Weinstein and 2-Point Discrimination Tests in Children. J Pediatr Orthop 2019; 39(2): 98-103.
12. Heriseanu R, Baguley IJ, Slewa-Younan S. Two-point discrimination following traumatic brain injury. J Clin Neurosci 2005; 12(2): 156-60.
13. Williamson A, Hoggart B. Pain: a review of three commonly used pain rating scales. J Clin Nurs 2005; 14(7): 798-804.
14. Birnie KA, Hundert AS, Lalloo C, Nguyen C, Stinson JN. Recommendations for selection of self-report pain intensity measures in children and adolescents: a systematic review and quality assessment of measurement properties. Pain 2019; 160(1): 5-18.
15. Bieri D, Reeve RA, Champion DG, Addicoat L, Ziegler JB. The Faces Pain Scale for the self-assessment of the severity of pain experienced by children: development, initial validation, and preliminary investigation for ratio scale properties. Pain 1990; 41(2): 139-50.
16. Beyer JE, Denyes MJ, Villarruel AM. The creation, validation, and continuing development of the Oucher: a measure of pain intensity in children. J Pediatr Nurs 1992; 7(5): 335-46.
17. Li AM, Yin J, Yu CC, et al. The six-minute walk test in healthy children: reliability and validity. Eur Respir J 2005; 25(6): 1057-60.
18. Baque E, Barber L, Sakzewski L, Boyd RN. Test-re-test reproducibility of activity capacity measures for children with an acquired brain injury. Brain Inj 2016; 30(9): 1143-9.
19. Fadida Y, Shapira-Vadler O, Spasser R, Frenkel-Toledo S. Reproducibility and smallest real differences of walking and Energy Expenditure Index in children and adolescents with an acquired brain injury. NeuroRehabilitation 2019; 45(1): 19-24.
20. Mayorga-Vega D, Aguilar-Soto P, Viciana J. Criterion-Related Validity of the 20-M Shuttle Run Test for Estimating Cardiorespiratory Fitness: A Meta-Analysis. J Sports Sci Med 2015; 14(3): 536-47.
21. Tomkinson GR, Lang JJ, Tremblay MS, et al. International normative 20 m shuttle run values from 1 142 026 children and youth representing 50 countries. Br J Sports Med 2017; 51(21): 1545-54.
22. Rossi C, Sullivan SJ. Motor fitness in children and adolescents with traumatic brain injury. Arch Phys Med Rehabil 1996; 77(10): 1062-5.

23. Becher JD, C. Folmer, K. Scholtes, V. Voorman, J. Wolterbeek, N. Handleiding Standaard Lichamelijk Onderzoek bij kinderen met een Centraal Motorische Parese: Bohn Stafleu van Loghum; 2019.
24. Katz-Leurer M, Rottem H, Meyer S. Hand-held dynamometry in children with traumatic brain injury: within-session reliability. Pediatr Phys Ther 2008; 20(3): 259-63.
25. van den Beld WA, van der Sanden GA, Sengers RC, Verbeek AL, Gabreels FJ. Validity and reproducibility of hand-held dynamometry in children aged 4-11 years. J Rehabil Med 2006; 38(1): 57-64.
26. Williams G, Morris ME, Schache A, McCrory P. Observational gait analysis in traumatic brain injury: accuracy of clinical judgment. Gait Posture 2009; 29(3): 454-9.
27. Aertssen WF, Ferguson GD, Smits-Engelsman BC. Reliability and Structural and Construct Validity of the Functional Strength Measurement in Children Aged 4 to 10 Years. Phys Ther 2016; 96(6): 888-97.
28. Ibey RJ, Chung R, Benjamin N, et al. Development of a challenge assessment tool for high-functioning children with an acquired brain injury. Pediatr Phys Ther 2010; 22(3): 268-76.
29. McArthur C, Venkatesh S, Warren D, et al. Further development of the response scales of the Acquired Brain Injury Challenge Assessment (ABI-CA). Brain Inj 2013; 27(11): 1271-80.
30. Wong RK, McEwan J, Finlayson D, et al. Reliability and validity of the acquired brain injury challenge assessment (ABI-CA) in children. Brain Inj 2014; 28(13-14): 1734-43.
31. van der Ploeg RJ, Oosterhuis HJ. [Physical examination--measurement of muscle strength]. Ned Tijdschr Geneeskd 2001; 145(1): 19-23.
32. Verschuren O, Ketelaar M, Takken T, Van Brussel M, Helders PJ, Gorter JW. Reliability of hand-held dynamometry and functional strength tests for the lower extremity in children with Cerebral Palsy. Disabil Rehabil 2008; 30(18): 1358-66.
33. Bongers BC, SI DEV, Helders PJ, Takken T. The steep ramp test in healthy children and adolescents: reliability and validity. Med Sci Sports Exerc 2013; 45(2): 366-71.
34. Braam KI, Van Dulmen-Den Broeder E, Veening MA, et al. Application of the steep ramp test for aerobic fitness testing in children with cancer. Eur J Phys Rehabil Med 2015; 51(5): 547-55.
35. Bruce RA, Kusumi F, Hosmer D. Maximal oxygen intake and nomographic assessment of functional aerobic impairment in cardiovascular disease. Am Heart J 1973; 85(4): 546-62.
36. Schulz J, Henderson SE, Sugden DA, Barnett AL. Structural validity of the Movement ABC-2 test: factor structure comparisons across three age groups. Res Dev Disabil 2011; 32(4): 1361-9.
37. Ko J, Kim M. Reliability and responsiveness of the gross motor function measure-88 in children with cerebral palsy. Phys Ther 2013; 93(3): 393-400.
38. Shepherd M Pt DOF, Louw A Pt P, Podolak J Pt DPT. The clinical application of pain neuroscience, graded motor imagery, and graded activity with complex regional pain syndrome-A case report. Physiother Theory Pract 2020; 36(9): 1043-55.
39. Hassett L, Moseley AM, Harmer AR. Fitness training for cardiorespiratory conditioning after traumatic brain injury. Cochrane Database Syst Rev 2017; 12(12): CD006123.
40. Mossberg KA, Amonette WE, Masel BE. Endurance training and cardiorespiratory conditioning after traumatic brain injury. J Head Trauma Rehabil 2010; 25(3): 173-83.
41. Hellweg S, Johannes S. Physiotherapy after traumatic brain injury: a systematic review of the literature. Brain Inj 2008; 22(5): 365-73.
42. Iaccarino MA, Bhatnagar S, Zafonte R. Rehabilitation after traumatic brain injury. Handb Clin Neurol 2015; 127: 411-22.
43. Heus I, Weezenberg D, Severijnen S, Vliet Vlieland T, van der Holst M. Measuring treatment outcome in children with developmental coordination disorder; responsiveness of six outcome measures. Disabil Rehabil 2022; 44(7): 1023-34.
44. Van Waelvelde H, Hellinckx T, Peersman W, Smits-Engelsman BC. SOS: a screening instrument to identify children with handwriting impairments. Phys Occup Ther Pediatr 2012; 32(3): 306-19.
45. Mathiowetz V, Kashman N, Volland G, Weber K, Dowe M, Rogers S. Grip and pinch strength: normative data for adults. Arch Phys Med Rehabil 1985; 66(2): 69-74.

46. Oxford Grice K, Vogel KA, Le V, Mitchell A, Muniz S, Vollmer MA. Adult norms for a commercially available Nine Hole Peg Test for finger dexterity. Am J Occup Ther 2003; 57(5): 570-3.
47. Smith YA, Hong E, Presson C. Normative and validation studies of the Nine-hole Peg Test with children. Percept Mot Skills 2000; 90(3 Pt 1): 823-43.
48. Krumlinde-Sundholm L, Holmefur M, Kottorp A, Eliasson AC. The Assisting Hand Assessment: current evidence of validity, reliability, and responsiveness to change. Dev Med Child Neurol 2007; 49(4): 259-64.
49. Holmefur MM, Krumlinde-Sundholm L. Psychometric properties of a revised version of the Assisting Hand Assessment (Kids-AHA 5.0). Dev Med Child Neurol 2016; 58(6): 618-24.
50. Louwers A, Krumlinde-Sundholm L, Boeschoten K, Beelen A. Reliability of the Assisting Hand Assessment in adolescents. Dev Med Child Neurol 2017; 59(9): 926-32.
51. Louwers A, Beelen A, Holmefur M, Krumlinde-Sundholm L. Development of the Assisting Hand Assessment for adolescents (Ad-AHA) and validation of the AHA from 18 months to 18 years. Dev Med Child Neurol 2016; 58(12): 1303-9.
52. Ermer J, Dunn W. The sensory profile: a discriminant analysis of children with and without disabilities. Am J Occup Ther 1998; 52(4): 283-90.
53. Carswell A, McColl MA, Baptiste S, Law M, Polatajko H, Pollock N. The Canadian Occupational Performance Measure: a research and clinical literature review. Can J Occup Ther 2004; 71(4): 210-22.
54. Ohno K, Tomori K, Sawada T, Seike Y, Yaguchi A, Kobayashi R. Measurement Properties of the Canadian Occupational Performance Measure: A Systematic Review. Am J Occup Ther 2021; 75(6).
55. Nott MT, Chapparo C. Cognitive strategy use in adults with acquired brain injury. Brain Inj 2020; 34(4): 508-14.
56. Juntorn S, Sriphetcharawut S, Munkhetvit P. Effectiveness of Information Processing Strategy Training on Academic Task Performance in Children with Learning Disabilities: A Pilot Study. Occup Ther Int 2017; 2017: 6237689.
57. Erp SS, E. . Impaired awareness of deficits and cognitive strategy use in occupational performance of persons with acquired brain injury (ABI). Irish Journal of Occupational Therapy 2020; Vol. 48 No. 2, 2020: pp. 101-15.
58. Resch C, Rosema S, Hurks P, de Kloet A, van Heugten C. Searching for effective components of cognitive rehabilitation for children and adolescents with acquired brain injury: A systematic review. Brain Inj 2018; 32(6): 679-92.
59. Sol ME, Verschuren O, Horemans H, et al. The effects of wheelchair mobility skills and exercise training on physical activity, fitness, skills and confidence in youth using a manual wheelchair. Disabil Rehabil 2022; 44(16): 4398-407.
60. Kwakkel G, Veerbeek JM, van Wegen EE, Wolf SL. Constraint-induced movement therapy after stroke. Lancet Neurol 2015; 14(2): 224-34.
61. Haslam C, Bazen-Peters C, Wright I. Errorless learning improves memory performance in children with acquired brain injury: a controlled comparison of standard and self-generation techniques. Neuropsychol Rehabil 2012; 22(5): 697-715.
62. Bertens D, Kessels RP, Fiorenzato E, Boelen DH, Fasotti L. Do Old Errors Always Lead to New Truths? A Randomized Controlled Trial of Errorless Goal Management Training in Brain-Injured Patients. J Int Neuropsychol Soc 2015; 21(8): 639-49.
63. Knuijt S, Kalf JG, van Engelen BGM, de Swart BJM, Geurts ACH. The Radboud Dysarthria Assessment: Development and Clinimetric Evaluation. Folia Phoniatr Logop 2017; 69(4): 143-53.
64. Paquier PFvM, M. van Dongen, H. R. Catsman-Berrevoets, C. Creten W. L. van Borsel, J. Normative data of 300 Dutch-speaking children on the Token Test. Aphasiology 2009.
65. Castellino SM, Tooze JA, Flowers L, Parsons SK. The peabody picture vocabulary test as a prescreening tool for global cognitive functioning in childhood brain tumor survivors. J Neurooncol 2011; 104(2): 559-63.
66. Waite MC, Theodoros DG, Russell TG, Cahill LM. Internet-based telehealth assessment of language using the CELF-4. Lang Speech Hear Serv Sch 2010; 41(4): 445-58.

67. Schlichting JS, L. Schlichting Test voor Taalbegrip: handleiding. . Houten: Bohn Stafleu van Loghum; 2010.
68. Geytenbeek JJ, Mokkink LB, Knol DL, Vermeulen RJ, Oostrom KJ. Reliability and validity of the C-BiLLT: a new instrument to assess comprehension of spoken language in young children with cerebral palsy and complex communication needs. Augment Altern Commun 2014; 30(3): 252-66.
69. Neils J, Baris JM, Carter C, et al. Effects of age, education, and living environment on Boston Naming Test performance. J Speech Hear Res 1995; 38(5): 1143-9.
70. del Toro CM, Bislick LP, Comer M, et al. Development of a short form of the Boston naming test for individuals with aphasia. J Speech Lang Hear Res 2011; 54(4): 1089-100.
71. Pankratz ME, Plante E, Vance R, Insalaco DM. The diagnostic and predictive validity of the Renfrew Bus Story. Lang Speech Hear Serv Sch 2007; 38(4): 390-9.
72. Suiter DM, Leder SB. Clinical utility of the 3-ounce water swallow test. Dysphagia 2008; 23(3): 244-50.
73. Neely JG, Cherian NG, Dickerson CB, Nedzelski JM. Sunnybrook facial grading system: reliability and criteria for grading. Laryngoscope 2010; 120(5): 1038-45.
74. Namasivayam AK, Huynh A, Granata F, Law V, van Lieshout P. PROMPT intervention for children with severe speech motor delay: a randomized control trial. Pediatr Res 2021; 89(3): 613-21.
75. Hayden D, Namasivayam AK, Ward R. The assessment of fidelity in a motor speech-treatment approach. Speech Lang Hear 2015; 18(1): 30-8.
76. Faulkner RA, Klock K, Gale JE. Qualitative research in family therapy: publication trends from 1980 to 1999. J Marital Fam Ther 2002; 28(1): 69-74.
77. Sori CF, Sprenkle DH. Training family therapists to work with children and families: a modified Delphi study. J Marital Fam Ther 2004; 30(4): 479-95.
78. Weiner SJ, Schwartz A, Weaver F, et al. Contextual errors and failures in individualizing patient care: a multicenter study. Ann Intern Med 2010; 153(2): 69-75.
79. Weiner SJ, Schwartz A. Contextual Errors in Medical Decision Making: Overlooked and Understudied. Acad Med 2016; 91(5): 657-62.

BIJLAGE: PROCESBESCHRIJVING

Procesbeschrijving - *Project 'Meedoen Next Step':*

1. Totstandkoming van het landelijk behandelprogramma
2. Relatie met Zorgstandaard en behandelkader
3. Implementatie en evaluatie
4. Landelijke inbedding en borging behandelprogramma
5. Regionale samenwerking
6. Ontwikkeling en Onderzoek - ambities in Nederland
7. Referenties

1. TOTSTANDKOMING VAN HET LANDELIJK BEHANDELPROGRAMMA

In 2008 is Hersenletsel en Jeugd (HeJ) opgericht: een landelijk samenwerkingsverband om zorg en onderwijs voor kinderen en jongvolwassenen met NAH te verbeteren. HeJ is aangesloten bij De Hersenletsel Alliantie. In elke regio is een centrum voor MSR en/of ziekenhuis met een gespecialiseerd, multidisciplinair team aanwezig om jongeren met NAH en hun gezin te behandelen/begeleiden/coachen, of snel en gericht door te verwijzen. Velen van hen hebben zich aangesloten bij HeJ. Vanuit HeJ wordt het belang gezien van de MSR als centrale speler om de juiste zorg-op het juiste moment-op de juiste plek voor jongeren met NAH en hun gezin te bieden.

Met het landelijk onderzoeksproject 'Meedoen?!' (2015 tot 2020) is een eerste stap gezet in het verkrijgen van inzicht in de 'minder zichtbare gevolgen' van niet-aangeboren hersenletsel (NAH): vermoeidheid, participatieproblemen, vermindering van kwaliteit van leven en impact op het gezin. Verschillende revalidatiecentra die aangesloten zijn bij HeJ, hebben meegewerkt aan dit project dat uitgevoerd werd door onderzoekers van Basalt, door bij een cohort kinderen en jongvolwassenen (4-25 jaar) met NAH systematisch gegevens te verzamelen over bovengenoemde thema's. 'Meedoen?!' toonde aan dat dit cohort op alle vier de uitkomsten beduidend meer problemen liet zien dan leeftijdgenoten.[1-4]

Door onderzoekers van de afdeling Innovation, Quality & Research (IQ+R) van Basalt Revalidatie is het projectplan voor 'Meedoen Next Step' succesvol ingediend voor financiering bij de Hersenstichting. Na honorering van het project zijn alle (16) centra voor MSR gevraagd deel te nemen: invoeren landelijke meetset, opstellen landelijk behandelprogramma, verbeteren netwerkzorg. Er sloten in totaal 13 centra aan als actieve partner en 1 als passieve partner. Een actieve partner stelde voor de projectduur een (of twee) 'lokale kartrekker(s)' aan, die in alle fasen van het project participeerde(n) in nauwe samenwerking met het team van het eigen centrum. De passieve partners waren bij het vormen van het behandelprogramma niet inhoudelijk betrokken, maar hebben zich wel gecommitteerd aan het gebruik van de meetset van 'Meedoen Next Step' en het gebruik van het landelijk behandelprogramma zodra deze klaar is.

In het project is er eerst ingezet op consensus over een vernieuwde 'Next Step meetset aan de poort' met gevalideerde vragenlijsten (Patient Reported Outcome Measures: PROMs). Deze vernieuwde meetset is sinds april 2021 digitaal beschikbaar en wordt sindsdien binnen de deelnemende centra voor MSR aan alle nieuwe poliklinische patiënten aangeboden. Vervolgens is de inhoud van het beoogde, gezamenlijk behandelprogramma stapsgewijs geïnventariseerd, geprioriteerd en vastgesteld middels een Delphi-studie. Hieraan werkten 82 professionals vanuit 7 verschillende disciplines mee. De inhoud van het behandelprogramma en de bijbehorende diagnostiek is verder gezamenlijk uitgewerkt door alle deelnemende partijen tijdens een symposium en consensusbijeenkomst. Het in dit document beschreven behandelprogramma is een afspiegeling en beschrijving van de 'current practice' in Nederland waarbij er gezamenlijk consensus bereikt is over wat 'best practice' zou moeten zijn, waar mogelijk onderbouwd vanuit de wetenschappelijk literatuur.

Via de kartrekkers werden ook de interne werkwijze (logistiek) per centrum voor MSR beschreven en werden wensen en behoeften met betrekking tot regionale samenwerking geïnventariseerd. Elk centrum maakte plannen t.b.v. de verbetering van de regionale samenwerking, gebruikmakend van beschikbare materialen en methodes uit andere regios.

Na de consensusbijeenkomst is het behandelprogramma verder uitgeschreven, is een implementatieplan opgesteld en hebben de kartrekkers zich gericht op de implementatie in het eigen centrum en de inbedding van het behandelprogramma in de regionale netwerkzorg.

2. RELATIE MET ZORGSTANDAARD EN BEHANDELKADER

Het behandelprogramma is opgesteld in lijn met de Zorgstandaard Traumatisch Hersenletsel (THL) kinderen & jongeren (in 2016 gepubliceerd en in 2022-2023 geüpdatet).[1] In deze zorgstandaard wordt beschreven wat er verwacht mag worden van de zorg voor kinderen en jongeren met traumatisch hersenletsel in Nederland. Zowel de acute fase (eerste uren tot weken na ontstaan letsel), als de subacute of herstelfase (vanaf medische stabilisatie tot maanden/jaren later) en de participatiefase (focus op dagelijks leven en re-integratie, tot jaren na ontstaan letsel) komen hierin aan bod. In de zorgstandaard is een ontwikkelagenda opgenomen met onder andere de volgende aanbevelingen, die mede de basis vormen voor dit behandelprogramma:[1]

a) Ontwikkeling van een landelijke visie op revalidatie behandeling voor kinderen en jongeren met NAH, die aansluit bij wensen en behoeften van de doelgroep en gezin en voldoet aan de huidige kennis vanuit de wetenschap.
b) Ontwikkeling van een behandelprogramma waarin is uitgewerkt wanneer en voor wie interventies zinvol zijn. Ook is eenduidigheid gewenst in richtlijnen voor (door)verwijzen, timing, intensiteit, duur, vorm en inhoud van de behandeling.
c) Er is behoefte aan onderzoek naar de effectiviteit van verschillende revalidatiebehandelingen, zowel op fysiek, cognitief en sociaal-emotioneel vlak
d) Ontwikkeling van voorlichting aan kinderen en jongeren met THL.
e) Ontwikkeling van een systeem voor registratie en monitoring van de kinderen en jongeren met THL, ook ten behoeve van risico- en voorspellende factoren en besluitvorming in het behandelproces.

f) Programma's en versterking van de ketenzorg waarin ook aandacht is voor een goede aansluiting van jeugdzorg naar volwassenzorg.

De Zorgstandaard THL kinderen en jongeren is een algemeen, doelgroep breed document.[1] Naast de Zorgstandaard beschrijft het 'Behandelkader Revalidatie van jongeren met NAH', opgesteld door de Nederlandse Vereniging van Revalidatieartsen (VRA) de minimale eisen waaraan een behandelteam voor MSR in een revalidatiecentrum of ziekenhuis moet voldoen om jongeren met NAH en hun gezin te kunnen behandelen of snel en gericht door te kunnen verwijzen.

3. IMPLEMENTATIE EN EVALUATIE

De implementatie van het behandelprogramma omvat:

- Uitvoering van een basis implementatieplan, dat door elk centrum op maat wordt aangepast.
- Doel is dat elke professional in de MSR zich houdt aan de afspraken over werkwijze en aanbod op basis van de gezamenlijke visie. Voor alle centra geldt dat er ruimte is voor centrum specifieke of regionale inkleuring waar nodig.
- De regionale inbedding van het behandelprogramma zal per regio verschillend zijn, met voor elke regio eigen verantwoordelijkheid in aanpak, tempo en beoogde opbrengst.

De implementatie van het behandelprogramma wordt geëvalueerd op gebruik, gebruiksvriendelijkheid, gebruikstevredenheid en volledigheid. Dit wordt gedaan middels vragenlijsten en interviews met de behandelteams waarbij gevraagd zal worden naar barrières en facilitatoren voor gebruik. De uitkomsten van deze vragenlijsten en interviews zullen worden beschreven en gedeeld met alle centra zodat men van elkaar kan leren.

De samenwerking binnen het onderzoek 'Meedoen Next Step' maakt het mogelijk om:

a) Voor de landelijke evaluatie van het behandelprogramma de resultaten van de PROMS, anoniem verzameld op T0, T1 en T2 (12 maanden na afronding van behandeling of 9 maanden na het eerste spreekuur in geval van geen behandeling) te gebruiken om de verandering over de tijd te kunnen monitoren. T.b.v. zorgevaluatie worden aan de T1 vragenlijst enkele vragen toegevoegd over tevredenheid (o.a. ankervraag). De basisvragen uit T0 (niet veranderende kenmerken) vervallen in de T1 en T2 meetset.

b) Op centrumniveau de effectiviteit op individueel niveau te bepalen: verschil tussen begin- en eindmeting klinimetrie. De resultaten van de begin- en eindmeting door zorgprofessionals (klinimetrie) worden ook op centrumniveau anoniem verzameld worden om de effecten van een revalidatiebehandeling te inventariseren.

c) Als het behandelaanbod geharmoniseerd is en goed geregistreerd wordt (specifieke interventies, frequentie, duur), tevredenheid en effecten te kunnen koppelen om zodoende het behandelprogramma doorlopend te evalueren.

Hierover worden na publicatie van dit behandelprogramma afspraken gemaakt. Op basis van (inter)nationale ontwikkelingen kan het behandelprogramma bijgesteld worden indien nodig. De Hersenletsel Alliantie is eigenaar van het landelijk behandelprogramma, met HeJ als mede-eigenaar, en is verantwoordelijk voor de borging.

4. LANDELIJKE INBEDDING EN BORGING BEHANDELPROGRAMMA

De basis voor de inbedding en borging van het behandelprogramma MSR ligt in het MSR-consortium van "Meedoen?!" (2015-2020), dat in 2020 is verduurzaamd en uitgebreid om deze "Next Step" gezamenlijk te zetten. Veertien centra hebben actief samengewerkt en hebben een gezamenlijk behandelprogramma opgezet. Een aantal centra hebben op de achtergrond meegedacht met het behandelprogramma.

De Hersenletsel Alliantie en de werkgroep revalidatie van HeJ worden eigenaar van het landelijk behandelprogramma en beschikken na "Meedoen Next Step" over de tools om het behandelprogramma doorlopend te monitoren en bij te stellen. Bij de actualisatie van het behandelprogramma zijn verschillende werkgroepen van HeJ betrokken: werkgroep Revalidatie (revalidatieartsen, psychologen), Ontwikkeling en Onderzoek (projectleiders, onderzoekers), Paramedisch (vanaf 2023 actief: fysiotherapeuten, ergotherapeuten, logopedisten, maatschappelijk werk), Onderwijs (leerkrachten, ambulant begeleiders) en Chronische fase (ambulant begeleiders).

De landelijke stuurgroep van HeJ zorgt voor verbinding met de Hersenletsel Alliantie en landelijke koepels. De evaluatie van verbeteringen in de netwerkzorg wordt in de regio zelf bepaald, afhankelijk van de gekozen werkwijze. Hiervoor zal wel een draaiboek op basis van 'good practice' worden gemaakt. Waar nodig kan i.s.m. de stuurgroep of HeJ initiatief worden genomen om regionale of landelijke verbeterpunten te adresseren. De Zorgstandaard THL fungeert hierbij als onderlegger. De uitkomsten van "Meedoen Next Step" worden door de HeJ stuurgroep via de sectie kinderrevalidatie bij de VRA gedocumenteerd en daarmee geborgd.

Verder is van belang, dat de werkgroep Ontwikkeling en Onderzoek van HeJ (O&O) het overzicht behoudt over lopende projecten en onderzoek voor jongeren met NAH. Door periodieke uitwisseling hierover tussen alle sleutelfiguren in innovatie en onderzoek in Nederland wordt ook getracht overlap te voorkomen, elkaar te ondersteunen in planvorming en uitvoering en gezamenlijk tot aanvragen te komen. Een overzicht van lopende projecten en onderzoek kan worden opgevraagd bij de werkgroep HeJ O&O.

5. REGIONALE SAMENWERKING

Een nevendoel in Next Step is het inbedden van het landelijk MSR-behandelprogramma in de regionale netwerkzorg en het bevorderen van samenwerking tussen regionale partners (o.a. huisartsen, ziekenhuizen, onderwijs, welzijn).

In elke regio bestaan afspraken over de samenwerking tussen netwerkpartners. Het verschilt per regio of en hoe deze afspraken zijn vastgelegd en welke wensen voor verbetering bestaan. Omdat de verschillen per regio aanzienlijk zijn, is voor de inbedding van dit MSR- behandelprogramma gekozen voor een regionale aanpak met de kartrekkers als spil. De kartrekkers waren de eerste aanspreekpunten en vertegenwoordigers van de deelnemende centra en werkten aan:

- Een inventarisatie van wensen en behoeften met betrekking tot regionale samenwerking (gemaakt met het eigen team). Deze resultaten hebben de kartrekkers onderling gedeeld en verbetervoorstellen besproken (bijvoorbeeld met betrekking tot het voortraject MSR en het proces van verwijzing, het

aanbieden van psycho-educatieve materialen en het beschikken over aanmeldingsgegevens voor de eerste poli te stroomlijnen), die verder regionaal worden uitgewerkt. Voor het natraject MSR zijn een goede overdracht naar netwerkpartners en een follow-upafspraak benoemd als belangrijke wens/aandachtspunt.

- Een plan om met de regionale partners in gesprek te gaan: een regionale expertmeeting, waarin elk centrum voor MSR met regionale netwerkpartners oplossingsgericht afspraken maakt om gezamenlijke wensen en behoeften te realiseren. Er wordt gewerkt aan interne kennis, samenwerking en uitwisseling met de eerstelijnszorg, ontwikkelen, versterken en behouden van netwerkzorg.

Nadere informatie met betrekking tot bovenstaande punten kan opgevraagd worden bij de betreffende kartrekkers (zie namen op voorblad).

DE CENTRA VOOR MSR BESTRIJKEN ELK EEN REGIO IN NEDERLAND

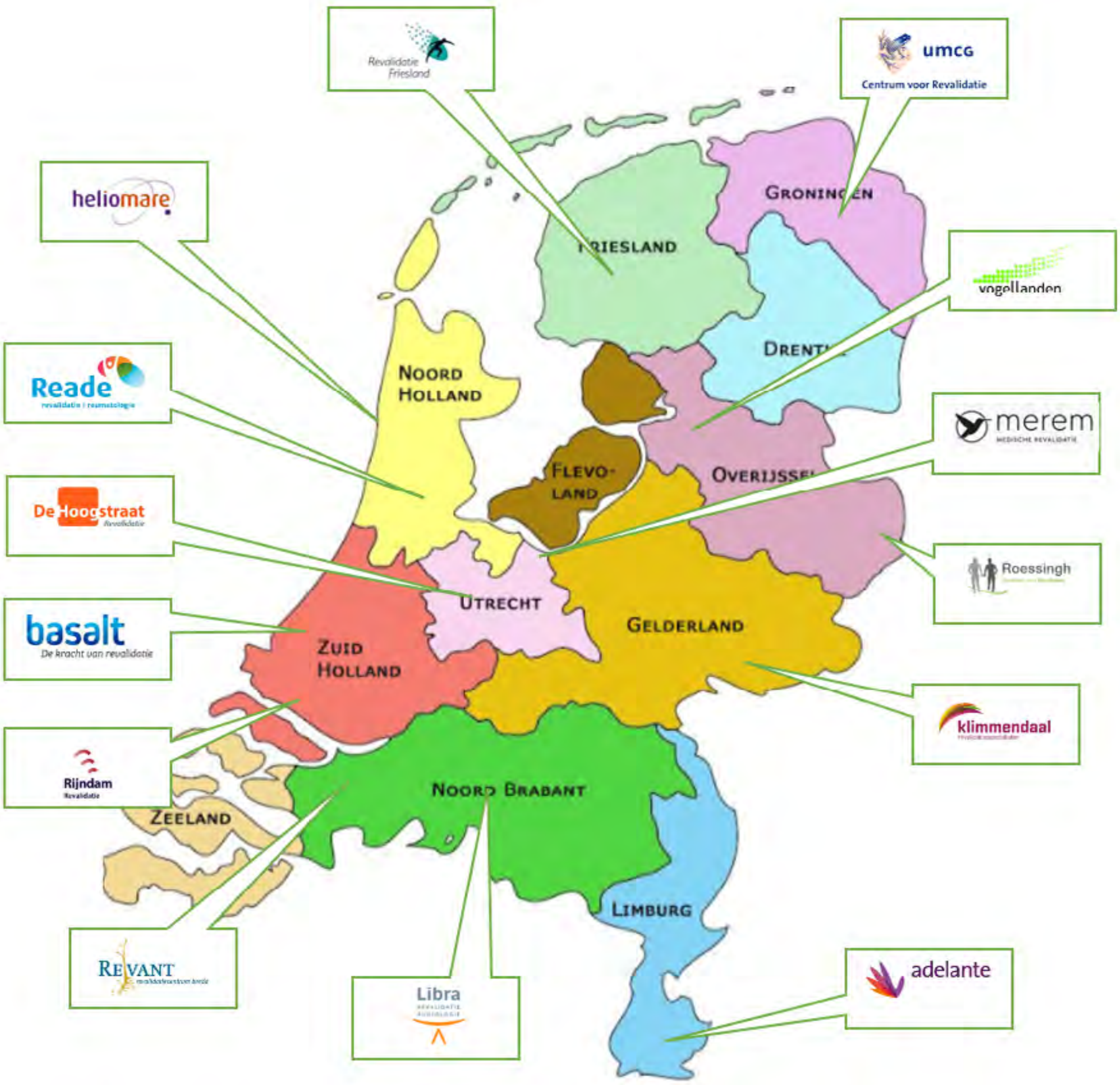

Om regionale afspraken en ambities vast te leggen kan een regionaal convenant tussen de netwerkpartners helpen commitment te waarborgen en het proces en de beoogde opbrengsten te faciliteren. Bijvoorbeeld afspraken over aanbod, aansluiting, overdracht, vindbaarheid en toegankelijkheid, maar ook gezamenlijk inzicht in aantallen en routing. In het convenant worden hiertoe afspraken gemaakt over tijds- en financiële investering en verantwoordelijkheden. Zo vergt regionale netwerkzorg regie van een netwerkpartner, b.v. om initiatief te nemen tot (jaarlijks) bijeenbrengen van het regionale netwerk, het monitoren van gemaakte afspraken en acties t.a.v. uitdagingen en hiaten binnen het netwerk.

In het convenant wordt ook duidelijk gemaakt waarom en hoe de netwerkpartners hier baat bij hebben, bijvoorbeeld omdat hierdoor de instroom en uitstroom in eigen organisatie bevorderd wordt en kwaliteit en resultaten bevorderd worden. Het uiteindelijke doel van het convenant is, dat In elke regio wordt een actueel navigatiesysteem gebruikt wordt om 'de beste zorg-op het juiste moment-op de juiste plek' te bewerkstelligen. Professionals van verschillende organisaties werken hierin samen en over de eigen grenzen van de organisatie heen.

De centra voor MSR wisselen de regionale informatie jaarlijks uit, de stuurgroep HeJ ontvangst deze informatie ook om overzicht op hoofdlijnen te bewaren.

Op bestuurlijk niveau is per centrum en liefst ook landelijk commitment nodig voor het implementeren en borgen van het behandelprogramma en de samenwerking met regionale netwerkpartners. In elk centrum voor MSR worden bestuurders via kartrekker/revalidatieartsen uitgebreid geïnformeerd over vervolgstappen en worden afspraken gemaakt over wat nodig is voor commitment (intern en extern), scholing medewerkers, implementatie en borging behandelprogramma, samenwerking met netwerkpartners en onderzoek. Het helpt om met onderbouwing vanuit landelijk onderzoek en goede voorbeelden van andere centra voor MSR aanvragen te doen voor benodigde tijd, scholing en andere voorwaarden. Ook is het van belang, dat bestuurders zich in regionale en landelijke gremia ambassadeurs voelen voor dit behandelprogramma.

6. ONTWIKKELING EN ONDERZOEK

AMBITIES IN NEDERLAND

De HeJ werkgroep Onderzoek en Ontwikkeling (O&O) startte in april 2010, samengesteld uit projectleiders en onderzoekers NAH jongeren in zorg/onderwijs/welzijn/onderzoeksinstellingen, met als ambities:

- Coördinatie en samenwerking: elkaar informeren over lopende en voorgenomen projecten en onderzoeken, expertise ontsluiten en uitwisselen, samenwerking zoeken, overlap voorkomen, efficiëntie vergroten, gezamenlijk aanvragen doen. In circa vier onlinebijeenkomsten per jaar een dynamisch, landelijk netwerk onderhouden. De voorzitter van O&O maakt deel uit van de landelijke stuurgroep HeJ.
- Overzicht: bijhouden van lopende projecten en onderzoeken, met korte beschrijving van doelstelling, werkwijze en contactpersonen. Dit document wordt zo actueel en volledig mogelijk bijgehouden door de voorzitter van de werkgroep O&O.

- Kennisdisseminatie: in samenwerking wordt kennis gedeeld met de collega's in zorg/onderwijs/welzijn/onderzoeksinstellingen, b.v. jaarlijks in de Brain Awareness Week. Ook vertegenwoordigt de werkgroep HeJ tijdens (inter)nationale symposia.
- Awareness vergroten: bestaan en impact van NAH bij jongeren op een goed onderbouwde manier onder de aandacht brengen, zowel breed maatschappelijk als bij professionals in zorg/onderwijs/welzijn/onderzoeksinstellingen.
- Ondersteuning HeJ: andere werkgroepen van HeJ indien gewenst ondersteunen.

Ambities

- Samenwerking: De revalidatiecentra verduurzamen de onderlinge samenwerking en de samenwerking met de acute zorg, onderwijs en welzijn en waarborgen zodoende 'de juiste zorg, op de juiste plek op het juiste moment' in het meerjarenbeleid van HeJ. Naast de bestaande HeJ-werkgroepen worden ook voor paramedische professionals (FT, ET, LO) en MW een werkgroep nagestreefd.
- Diagnose overstijgend: onderdelen van het behandelprogramma lijken goed geschikt voor andere doelgroepen. Afstemming met relevante samenwerkingsverbanden, b.v. jongeren met Cerebrale Parese, wordt geïntensiveerd.
- Awareness: Er wordt een HeJ 'toolbox voorlichting en scholing' samengesteld met materialen en presentaties, die gebruikt kunnen worden in de verschillende regio's.
- Disseminatie: Jaarlijks wordt in de Brain Awareness Week in maart een congres georganiseerd door de stuurgroep van HeJ. Een samenvatting hiervan wordt via Revalidatie Magazine en Revalidatie Kennisnet gedeeld en staat bij elk centrum voor MSR op de site.
- Behandelprogramma MSR: De huidige meetset (PROMS) wordt periodiek geüpdatet, evenals het behandelprogramma. De Hersenletsel Alliantie en de HeJ werkgroep Revalidatie voeren hierover de regie. Hierbij wordt gebruik gemaakt van de 'toolbox' van de International Pediatric Brain Injury Society (IPBIS).
- Gezamenlijk onderzoek: de huidige samenwerking en onderzoek structuur wordt benut om het behandelprogramma doorlopend te evalueren en ook (nieuwe) interventies of andere onderzoeksvragen te kunnen toetsen. Hiervoor wordt voor meerdere jaren financiële ondersteuning gevonden en o.a. geanticipeerd op het kunnen aantonen van de effectiviteit van het behandelprogramma richting zorgverzekeraar. Zodra het Kenniscentrum NAH+ is gestart, wordt structureel vanuit O&O afgestemd over een landelijke onderzoek agenda NAH.

7. REFERENTIES

1. Allonsius F, de Kloet A, Bedell G, et al. Participation Restrictions among Children and Young Adults with Acquired Brain Injury in a Pediatric Outpatient Rehabilitation Cohort: The Patients' and Parents' Perspective. Int J Environ Res Public Health 2021; 18(4).
2. Allonsius F, De Kloet AJ, Van Markus-Doornbosch F, et al. Parent-reported family impact in children and young adults with acquired brain injury in the outpatient rehabilitation setting. Brain Inj 2021: 1-11.
3. Allonsius F, van Markus-Doornbosch F, de Kloet AJ, Lambregts S, Vliet Vlieland T, van der Holst M. Fatigue in young patients with acquired brain injury in the rehabilitation setting: Categorizing and interpreting fatigue severity levels. Dev Neurorehabil 2022: 1-12.
4. van Markus-Doornbosch F, van der Holst M, de Kloet AJ, Vliet Vlieland TPM, Meesters JJL. Fatigue, Participation and Quality of Life in Adolescents and Young Adults with Acquired Brain Injury in an Outpatient Rehabilitation Cohort. Dev Neurorehabil 2020; 23(5): 328-35.
