# Supplementary material for: A national consensus-based framework on preferred assessments and interventions in current treatment for young people with acquired brain injury in Dutch rehabilitation centers
Source: J Pediatr Rehabil Med. 2025 Apr 23:18758894251337581. Online ahead of print. doi: 10.1177/18758894251337581 (PMC13292738; doi:10.1177/18758894251337581)
Supplement: Supplementary file 2 [file sj-docx-1-prm-10.1177_18758894251337581.docx]

**Supplementary information.** Fourteen participating rehabilitation centers specialized in providing outpatient rehabilitation for young patients with acquired brain injury on the map of the Netherlands.

Participating Rehabilitation Centers: 1. Heliomare, Wijk aan Zee; 2. Reade, Amsterdam; 3. Merem, Hilversuim; 4. de Hoogstraat, Utrecht; 5. Basalt, The Hague; 6. Rijndam, Rotterdam; 7. Revant, Breda; 8. UMCG/Beatrixoord, Groningen; 9. Revalidatie Friesland, Beetsterzwaag; 10. Vogellanden, Zwolle; 11. Roessingh, Enschede; 12. Klimmendaal, Arnhem; 13. Libra, Eindhoven; 14. Adelante, Valkenburg.

* For centers with multiple locations, only the location of the primary/largest is shown.
